# Environment of Solvent-Controlled Chemoselective Asymmetric Hydroperoxidation and Hydroxylation of 5-Pyrazolone Ketimines Catalyzed by Bifunctional Organocatalysts

**DOI:** 10.1021/acsomega.4c10608

**Published:** 2025-03-11

**Authors:** Xiangfeng Lin, Bo Long, Hanhui Lei, Terence Xiaoteng Liu, Zhanhui Yuan

**Affiliations:** 1 College of Materials Engineering, Fujian Agriculture and Forestry University, Fuzhou 350108, China; 2 College of Mechanical and Electrical Engineering, Fujian Agriculture and Forestry University, Fuzhou 350108, China; 3 Department of Mechanical and Construction Engineering, Northumbria University, Newcastle upon Tyne NE1 8ST, United Kingdom

## Abstract

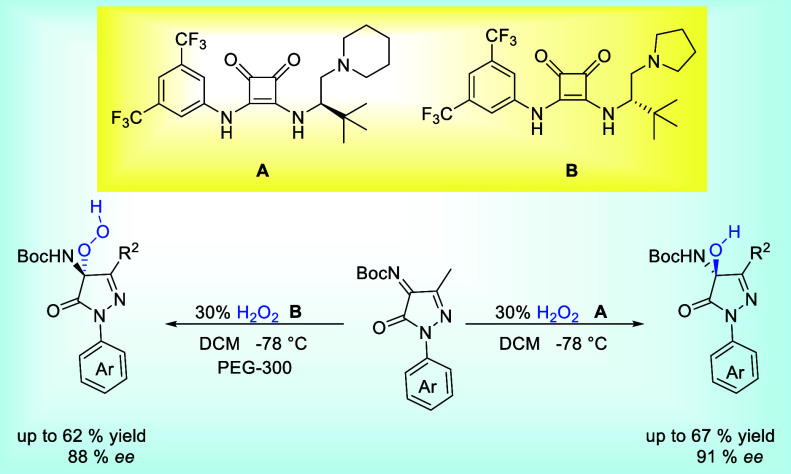

Chemoselectivity often garners significant attention in organic synthesis, serving as a primary strategy for producing a variety of functionalized products from the same substrate. The first environment of solvent-controlled chemoselective asymmetric hydroperoxidation and hydroxylation of 5-pyrazolone ketimines has been achieved, using an acid–base bifunctional chiral squaramide as the organocatalyst, affording a range of enantioenriched products of hydroperoxidation and hydroxylation with high stereoselectivities (up to 90% *ee*).

## Introduction

Chemoselectivity usually attracts much attention in organic synthesis, which has become a main strategy for getting different functional products with the same substrate and is meaningful to improve molecular diversity.^1−8^ In addition, chemoselective oxidation is an important way to get different oxidation products such as alcohol, aldehyde, and ester.^9−12^ For the reason that chemoselective oxidation by oxygen is difficult to control the selectivity of chemoselective oxidation by oxygen, it is getting interest from more and more researchers. The number of biologically interesting natural products with peroxide or epoxide structure motifs that possess anticancer, antitumor, and antimalarial activities is substantial and still growing.^13−19^ Asymmetric epoxidation or peroxidation was processed through the addition of substrate with hydroperoxides catalyzed by bifunctional organocatalysts,^20−28^ phosphoric acid,^29^ or metal Lewis acid.^30^ However, although much research about epoxidation or peroxidation has been reported, chemoselective asymmetric reactions with hydroperoxides are still rare. One example is reported by Deng's group in 2008;^20^ significant progress has been achieved in catalytic enantioselective peroxidation and epoxidation of unsaturated ketones with ROOH. The other is reported by List's group;^22^ they conducted cinchona primary amine-catalyzed asymmetric epoxidation and hydroperoxidation of unsaturated carbonyl compounds with hydrogen peroxide.

**Scheme 1:**
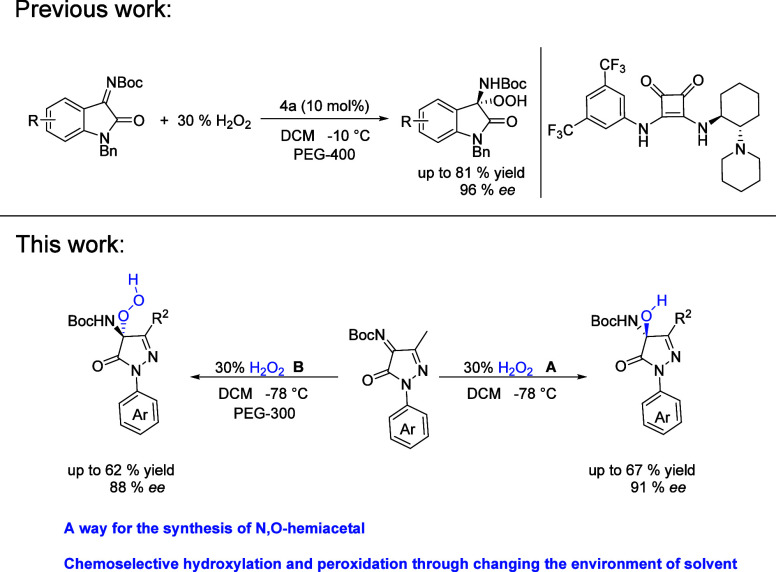
Environment of Solvent-Controlled Chemoselective Asymmetric Hydroperoxidation and Hydroxylation of 5-Pyrazolone Ketimines Catalyzed by Organic Base

Chiral α-amino peroxide moieties, obtained by the reaction of imine and hydroperoxides, are usually found in natural products. Since the first successful formation of α-amino peroxides through catalytic peroxidation of adamines using a chiral phosphoric acid was reported in 2010 by Antilla's group,^31^ significant progress has been achieved in developing enantioselective catalytic methodologies for this purpose.^32,33^ In contrast, chiral hydroperoxides (ROOH) are rare for the reason that they are highly reactive and transfer to the products of the Baeyer–Villiger reaction^34,35^ or epoxidation.^20−28^ In their recent work, Li's group found in the presence of PEG isatin-derived hydroperoxides that were isolated with excellent stereoselectivities catalyzed by an organic base through 1,2-hydroperoxidation of isatin-derived ketimines.^36^ The intermolecular HB between the PEG and −OOH groups has an influence on the control of chemoselectivities like supramolecular catalysis^37^ (Scheme 1). Pyrazolinone ketimines are similar to isatin-derived ketimines in structure.^38−42^ Therefore, we envision that they may have a similar chemical property. On the other hand, *N*,*O*-acetals are often obtained by the addition of oxygen-based nucleophiles to imine substrates, to produce numerous biologically significant compounds.^43−46^ However, *N*,*O*-hemiacetals, whose structure can be found in zampanolide^47^ and verruculogen,^48^ are rarely isolated because of the deprotection of *N*,*O*-hemiacetals. In this work, we developed an environment of solvent-controlled chemoselective asymmetric hydroperoxidation and hydroxylation of 5-pyrazolone ketimines^49−51^ catalyzed by an organic base (Scheme 1).

## Results and Discussion

First, we envisioned that hydroperoxidation would take place via the reaction of 5-pyrazolone ketimine **1a** and commercial hydrogen peroxide (30%) catalyzed by bifunctional organocatalyst **A**, of which **3a** was the target mode. However, **2a** was isolated in moderate yield (65%) and 90% *ee* (Table 1, entry 1). We then extended the catalyst screening by using other bifunctional organocatalysts **B**–**E** (Table 1, entries 2–5). In the corresponding reaction, moderate results were achieved in terms of yield and enantioselectivity. Bifunctional organocatalyst **A** was chosen for further optimization studies. Further investigations were carried out by screening other solvents. When the reaction was conducted in CHCl_3_, enantioselectivity was lower than the reaction in DCM (Table 1, entry 11). The optimized condition of product **2a** was carried out with 10% catalyst **A** in solvent DCM (Table 1, entry 1).

**Table 1:**
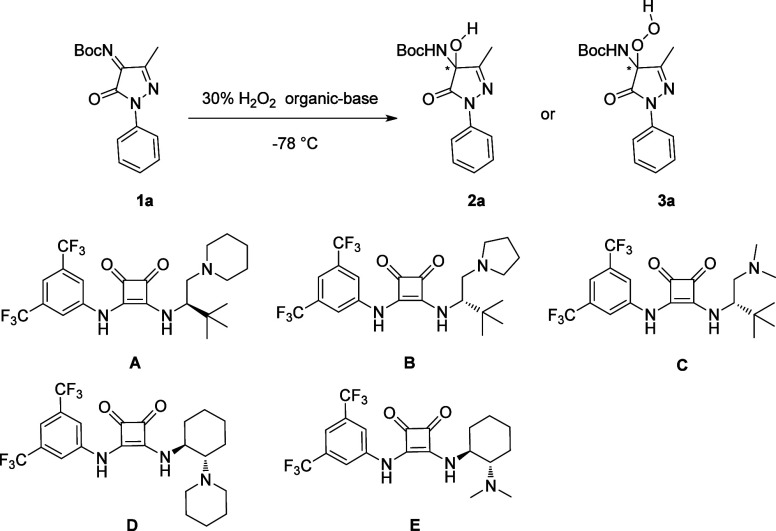
Optimization of the Hydroxylation of Pyrazolinone Ketimines^a^

entry	catalyst	solvent	additive	2a:3a^b^	yield (%)^c^	Ee (%)^d^
1	A	DCM		100:0	65	90
2	B	DCM		100:0	65	–77
3	C	DCM		100:0	59	–83
4	D	DCM		100:0	61	70
5	E	DCM		100:0	59	50
6	A	THF		0:100	73	65
7	B	THF		0:100	77	–75
8	C	THF		0:100	70	–71
9	D	THF		0:100	76	30
10	E	THF		0:100	75	53
11^e^	A	CHCl_3_		100:0	70	60
12	B	Et_2_O		0:100	71	–75
13	B	EA		0:100	55	–55
14^f^	B	DCM	PEG-300	1:3	62^f^	–86^f^
15^f^	B	DCM	PEG-400	1:2	58^f^	–83^f^
16^f^	B	DCM	PEG-600	1:3	63^f^	–80^f^
17^g^	B	DCM	THF	1:3	59	–76
18^h^	B	DCM	PEG-300	1:4	64^h^	–73^h^

a All reactions were carried out on a 0.1 mmol scale with 5 equiv of H_2_O_2_, and 10 mol % of catalyst in DCM (1 mL).

b Tested by ^1^HNMR.

c Isolated yield.

d *ee* was determined by chiral HPLC (column ADH-039).

e The temperature of the reaction is −60 °C.

f 30 mg of PEG was added.

g 30 μL of THF was added.

h 60 mg of PEG-300 was added. Yield contains **2a** and **3a**, and *ee* of **3a** was tested.

Interestingly, when the reaction was conducted in THF, **3a** was the major product (Table 1, entry 6). Other squaramide catalysts **B**–**E** were screened for optimized yield and enantioselectivity (Table 1, entries 7–10). Bifunctional organocatalyst **B** was the best catalyst for further investigation. Similarly, when the reaction was conducted in EA or Et_2_O, **3a** was the major product (Table 1, entries 12 and 13), which possibly suggests that the hydrogen-bonding (HB) donor solvent can induce the formation of **3a**. In order to verify the idea, several PEGs were used as additives and DCM was used as solvent (Table 1, entries 14–16). The product of hydroperoxidation was isolated. There are no obvious gaps in conversion and enantioselectivity when different PEGs were used as additives. **3a** was also isolated in 59% yield and 76% *ee* when THF was used as additive (Table 1, entry 17). Enantioselectivity decreased when the amount of PEG was overused (Table 1, entry 18). To further explore the efficiency of this catalytic system, the model reaction of formation of **3a** was carried out with 10% catalyst **B**, PEG-300 as additive, and DCM as solvent (Table 1, entry 14).

**Scheme 2:**
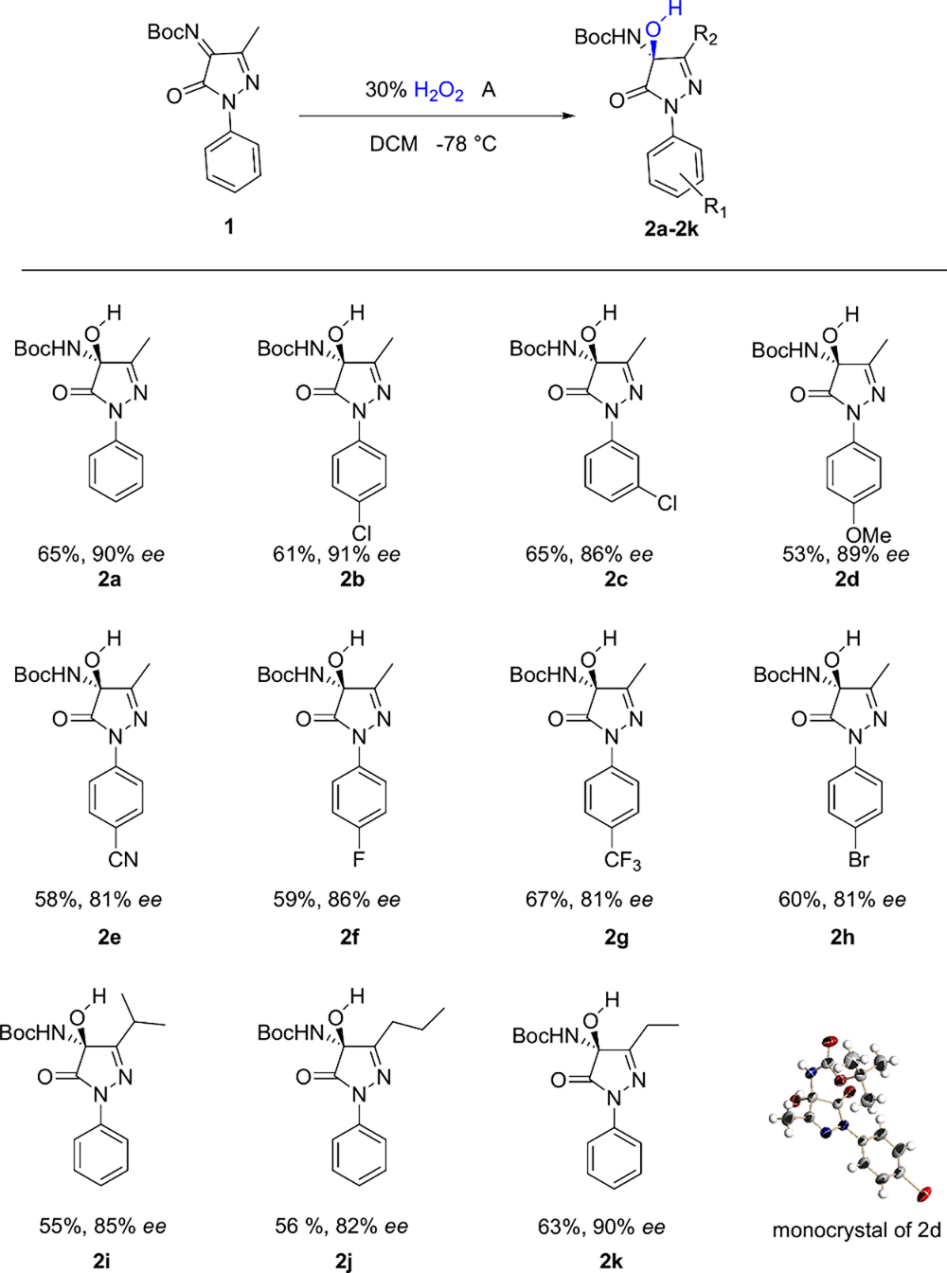
Substrate Scopes of Hydroxylation of Pyrazolinone Ketimines with Catalyst A All reactions were carried out on a 0.2 mmol scale with 10 equiv of H_2_O_2_, and 10 mol % of catalyst in DCM (2 mL).

With optimized conditions (Table 1, entry 1), substrate scopes of hydroxylation of pyrazolinone ketimines were investigated. The reaction of different pyrazolinone ketimines **1** with H_2_O_2_ provided the desired products **2a**–**2k** in moderate yields and high enantioselectivity (Scheme 2). Notably, the reaction tolerated a variety of functional groups on the aryl ring (**2a**–**2h**). In general, the products with electron-withdrawing groups in the aryl ring exhibited a little lower ee than electron-donating groups in the aryl ring. When the 3′ position of pyrazolinone ketimines was hinder, the product exhibited lower ee and yield (**2i**–**2k**). Notably, products **2** are unstable due to retro-reactions.

**Scheme 3:**
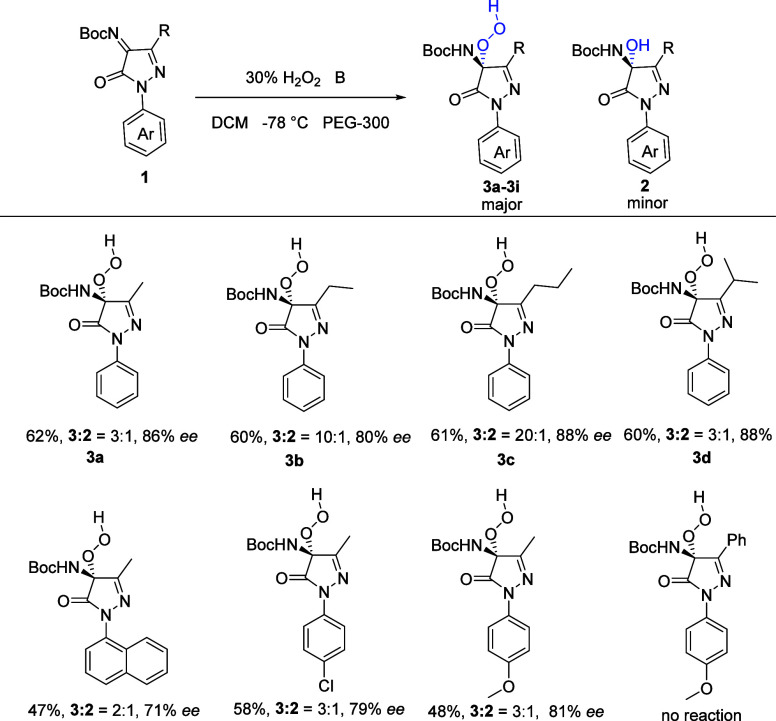
Substrate Scope of Hydroperoxidation of Pyrazolinone Ketimines with Catalyst B All reactions were carried out on a 0.2 mmol scale with 5 equiv of H_2_O_2_, 60 mg of PEG-300, and 10 mol % of catalyst in DCM (2 mL). *^a^*Yield contains **2** and corresponding **3**, and *ee* of 3 was tested.

With optimized conditions, substrate scopes of hydroperoxidation of pyrazolinone ketimines were also investigated, employing various pyrazolinone ketimines **1**, providing the desired products **3a**–**3k** in moderate yields and high enantioselectivity (Scheme 3). When the 3′ position of pyrazolinone ketimines was more hindered, the product exhibited similar ee and yield (**3b**–**3c**). Similarly with the results of hydroxylation, the products with electron-withdrawing groups in the aryl ring exhibited a little lower *ee* than electron-donating groups in the aryl ring. The peroxides are also unstable because of the oxidizing nature of the products. We also attempted to scale up the reaction to 1 mmol. However, the reactions became messy, primarily due to the instability of the products and the exothermic nature of the reaction.

**Scheme 4:**
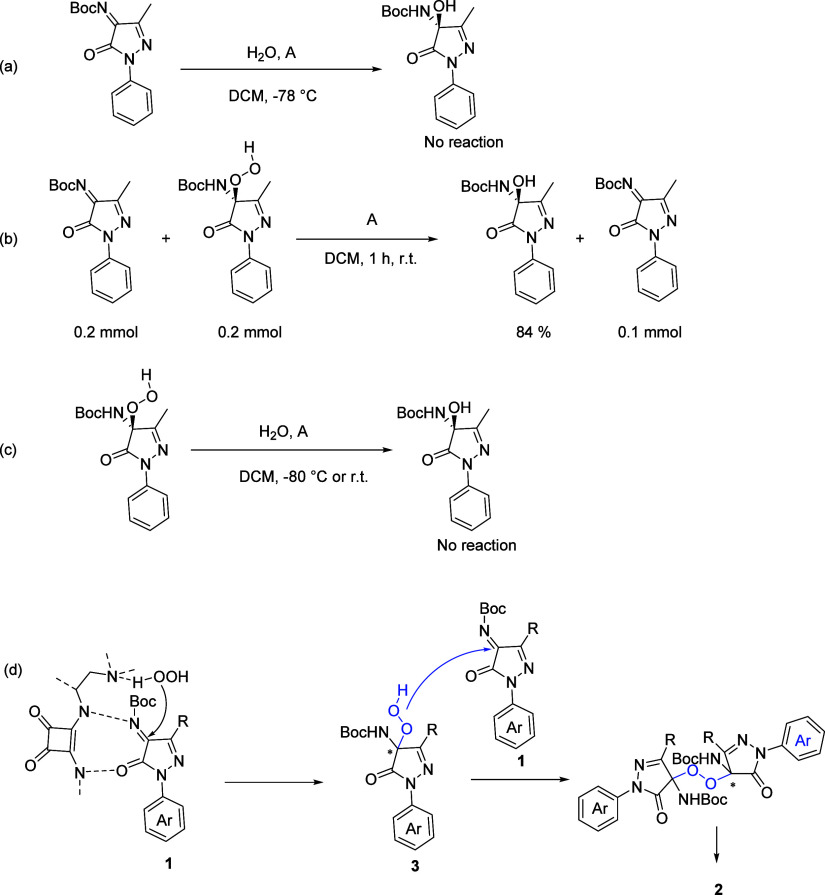
Controlled Experiment and Proposed Mechanism

To gain insights into the mechanism of this methodology, particularly the generation of the product of hydroxylation, some controlled experiments were carried out. First, when using water as a nucleophilic reagent instead of H_2_O_2_ in the same catalytic condition as hydroxylation, the reaction did not proceed at all. This result suggested that the water in H_2_O_2_ was not involved in the catalytic system (Scheme 4a). When the product of hydroperoxidation **3a** was used as oxidant, **1a** as substrate, the product of hydroxylation **2a** was isolated in 84% yield. Meanwhile, 50% substrate was recycled (Scheme 4b). However, the product of hydroperoxidation **3a** did not transfer to **2a** without **1a** (Scheme 4c). These two results indicated that the product of hydroxylation was generated from the product of hydroperoxidation, whose substrate played a role of reductant in this catalytic system. On the substrate, there may be two or more reductive sites, consuming two or more hydroperoxide products, which explained that the yield was more than 50%. In addition, from the results of LC-MS, we also captured the peak of [m+16], [m+32], and [m+48] (see the Supporting Information). However, we did not separate the oxidation products, which suggests it might be unstable. According to the controlled experiments, we proposed the possible mechanism (Scheme 4d). Initially, the nucleophilic addition occurred between H_2_O_2_ and substrates **1** catalyzed by the bifunctional organocatalyst, delivering the hydroperoxidation products **3**. Subsequently, products **3** attacked substrates **1** to give the dimers, followed by the hydrolysis of dimers to format the hydroxylation products **2**.

## Conclusions

In summary, we developed a bifunctional organocatalyzed chemoselective asymmetric 1,2-hydroperoxidation and hydroxylation of pyrazolinone ketimines, thus providing a direct and highly efficient approach for the synthesis of α-*N*-substituted hydroperoxides and alcohols. In all cases, a mediated yield and high enantioselectivities were achieved. The water in the catalytic system might not benefit the yield. Mechanism studies revealed that two products were generated from the same path, which could be controlled through monitoring the environment of solvent. The hydrogen-bonding solvent as an additive is beneficial to the generation of hydroperoxides. This strategy offers a potential opportunity for the discovery of solvent-controlled chemoselective asymmetric oxidation. Further work will be devoted to the development of new chemoselective asymmetric reactions.

## Experimental Section

### General Experimental Procedure of Asymmetric Hydroxylation

To a 10 mL test tube were sequentially added catalyst A (0.02 mmol, 9.8 mg), CH_2_Cl_2_ (2.0 mL), and the pyrazolinone ketimine **1** (0.2 mmol). The mixture was cooled to −80 °C and stirred for 10 min. 30% H_2_O_2_ (1.0 mmol, 5 equiv) was then added. The reaction mixture was stirred at −80 °C and monitored by TLC. Upon completion (12–24h), the residual was purified by silica gel flash chromatography (petroleum ether:ethyl acetate, 5:1) to afford the desired product **2**. The racemic examples were prepared by the catalysis of DABCO at r.t.

### General Experimental Procedure of Asymmetric Hydroperoxidation

To a 10 mL test tube were sequentially added catalyst B (0.02 mmol, 9.5 mg), CH_2_Cl_2_ (2.0 mL), PEG-300 (50 mg), and the pyrazolinone ketimine **1** (0.2 mmol). The mixture was cooled to −80 °C and stirred for 10 min. 30% H_2_O_2_ (1.0 mmol, 5 equiv) was then added. The reaction mixture was stirred at −80 °C and monitored by TLC. Upon completion (12–24 h), the residual was purified by silica gel flash chromatography (petroleum ether:ethyl acetate, 5:1) to afford the desired product **2**. The racemic examples were prepared by the catalysis of DABCO with the environment of PEG at −80 °C.

## Supporting Information Available

Crystallographic data (CIF)
Experimental details and procedures, characterization and crystallographic data, and spectra of all unknown compounds (PDF)

## Supplementary Material

ao4c10608_si_001.cif
