# Supplementary material for: Environment of Solvent-Controlled Chemoselective Asymmetric Hydroperoxidation and Hydroxylation of 5-Pyrazolone Ketimines Catalyzed by Bifunctional Organocatalysts
Source: ACS Omega. 2025 Mar 11;10(11):11225–30. doi: 10.1021/acsomega.4c10608 (PMC11947773; doi:10.1021/acsomega.4c10608)
Supplement: Supplementary file 2 — ao4c10608_si_002.pdf [file ao4c10608_si_002.pdf]

Environment of Solvents Controlled Chemoselective Asymmetric Hydroperoxidation and Hydroxylation of 5-Pyrazolone Ketimines Catalyzed by Bifunctional Organocatalysts

Xiangfeng Lin[1#], Bo Long[2#], Hanhui Lei[3*], Terence Xiaoteng Liu[3*], Zhanhui Yuan[1*]

[1]College of Materials Engineering, Fujian Agriculture and Forestry University, Fuzhou 350108, P. R. China

[2]College of Mechanical and Electrical Engineering, Fujian Agriculture and Forestry University, Fuzhou 350108, P. R. China

[3]Department of Mechanical and Construction Engineering, Northumbria University, Newcastle upon Tyne NE1 8ST, United Kingdom

E-mail address: Terence.liu@northumbria.ac.uk zhanhuiyuan@fafu.edu.cn ray.lei@northumbria.ac.uk

Contents

1. General information……………………………………………………………………………………..S3
2. Experimental procedure and spectral data of products……………………………………………………….S3
3. X-ray crystallographic data of 4e……………………………………………………………...………....S9
4. 1H NMR and 13C NMR spectra……………………………………………………………………….S22
5. HPLC spectra…………………………………………………………………………………………..S40
6. LC-MS of [m+16], [m+32], [m+48]……………………………………………………………………...S58

1. General Information

Reactions were monitored by thin layer chromatography (TLC), and column chromatography purifications were carried out using silica gel. 1H and 13C spectra were recorded on a 400 MHz spectrometer (100 MHz for 13C). The following abbreviations were used to designate chemical shift multiplicities: s = singlet, d = doublet, t = triplet, q = quartert, m = multiplet, br = broad. All first-order splitting patterns were assigned on the basis of the appearance of the multiplet. Splitting patterns that could not be easily interpreted are designated as multiplet (m) or broad (br). Column chromatography was performed on silica gel (300-400 mesh). HPLC analysis was performed on Agilent HPLC 1100 equipped with Daicel chiral AD-H column. High resolution mass spectra for all the new compounds were done by an LTQ-Orbitrap instrument (ESI) (Thermo Fisher Scientific, USA). Catalysts were purchased from Daicel Chiral Technologies (China) Co., LTD. Substrates 1 were synthesized by following the published procedures[1]. 30 % H_2O_2 and PEG was purchased from Sinopharm Chemical Reagent Co.,Ltd.

2. Experimental Procedure and Spectral Data of Products

General Experimental Procedure of Aymmetric Hydroxylation

To a 10-mL test-tube were sequentially added catalyst A (0.02 mmol, 9.8 mg), CH_2Cl_2 (2.0 mL), and the pyrazolinone ketimine 1 (0.2 mmol). The mixture was cooled to -80℃ and stirred for 10 min. 30% H_2O_2 (1.0 mmol, 5 eq) was then added. The reaction mixture was stirred at -80℃ and monitored by TLC. Upon completion (12-24h), the residual was purified by silica gel flash chromatography (petroleum ether:ethyl acetate, 5:1) to afford the desired product 2. The racemic examples were prepared by the catalysis of DABCO in r.t..

General Experimental Procedure of Aymmetric Hydroperoxidation

To a 10-mL test-tube were sequentially added catalyst B (0.02 mmol, 9.5 mg), CH_2Cl_2 (2.0 mL), PEG-300(50 mg) and the pyrazolinone ketimine 1 (0.2 mmol). The mixture was cooled to -80℃ and stirred for 10 min. 30% H_2O_2 (1.0 mmol, 5 eq) was then added. The reaction mixture was stirred at -80℃ and monitored by TLC. Upon completion (12-24h), the residual was purified by silica gel flash chromatography (petroleum ether:ethyl acetate, 5:1) to afford the desired product 2. The racemic examples were prepared by the catalysis of DABCO with the environment of PEG in -80℃.

Spectral Data of Products

***tert*-butyl (*S*)-(4-hydroxy-3-methyl-5-oxo-1-phenyl-4,5-dihydro-1H-pyrazol-4-yl)carbamate 2a**

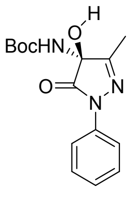

White soild, yield 65 %, ^{1}H NMR (400 MHz, $CDCl_3$) δ = 7.88 (d, *J*=8.0, 2H), 7.38 (t, *J*=7.9, 2H), 7.19 (t, *J*=7.4, 1H), 6.13 (s, 1H), 2.20 (s, 3H), 1.33 (s, 9H); ^{13}C NMR (101 MHz, $CDCl_3$) δ = 169.8, 159.5, 153.4, 137.5, 128.9, 125.4, 118.8, 82.8, 28.0, 12.4.

Enantiometric excess of the product was determined by chiral stationary phase HPLC analysis using Daicel AD-H column (*n*-Hexane/*i*-PrOH 80:20 at 1.0 ml/min, λ = 230 nm, t_{minor} = 5.5 min, t_{majorr} = 8.0 min, 90% *ee*, [α]20 D = 97.49 (*c* = 0.2, in MeOH).; HRMS (ESI) m/z calcd for $C_{15}H_{19}N_3O_4$, $[M+Na]^+$: 328.1273, found: 328.1283.

***tert*-butyl (*S*)-(1-(4-chlorophenyl)-4-hydroxy-3-methyl-5-oxo-4,5-dihydro-1H-pyrazol-4-yl)carbamate 2b**

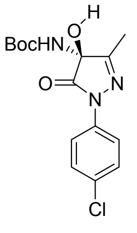

White soild, yield 61 %, ^{1}H NMR (400 MHz, $CDCl_3$) δ = 7.86 (d, *J*=9.0, 2H), 7.35 (d, *J*=9.0, 2H), 5.79 (s, 2H), 2.20 (s, 3H), 1.34 (s, 9H); ^{13}C NMR (101 MHz, $CDCl_3$) δ = 169.9, 159.8, 153.3, 136.1, 130.6, 129.0, 119.8, 82.8, 28.0, 12.3. Enantiometric excess of the product was determined by chiral stationary phase HPLC analysis using Daicel AD-H column (*n*-Hexane/*i*-PrOH 80:20 at 1.0 ml/min, λ = 230 nm, t_{minor} = 5.5 min, t_{major} = 8.9 min, 86% *ee*, [α]20 D = 81.49 (*c* = 0.2, in MeOH).; HRMS (ESI) m/z calcd for $C_{15}H_{18}N_3O_4Cl$, $[M+Na]^+$: 362.0884, found: 362.0879.

***tert*-butyl (*S*)-(1-(3-chlorophenyl)-4-hydroxy-3-methyl-5-oxo-4,5-dihydro-1H-pyrazol-4-yl)carbamate 2c**

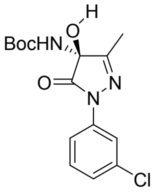

White soild, yield 65 %, ^{1}H NMR (400 MHz, $CDCl_3$) δ = 7.94 (t, *J*=2.0, 1H), 7.83 (dd, *J*=8.3, 1.1, 1H), 7.31 (t, *J*=8.2, 1H), 7.16 (ddd, *J*=8.0, 1.9, 0.8, 1H), 6.03 (s, 1H), 2.21 (s, 3H), 1.33 (s, 9H); ^{13}C NMR (101 MHz, $CDCl_3$) δ = 169.9, 159.7, 153.3, 138.5, 134.7, 130.0, 125.4, 118.6, 116.5, 82.8, 77.2, 28.0, 12.4.

Enantiometric excess of the product was determined by chiral stationary phase HPLC analysis using Daicel AD-H column (*n*-Hexane/*i*-PrOH 80:20 at 1.0 ml/min, λ = 230 nm, t_{minor} = 4.8 min, t_{major} = 7.1 min, 91% *ee*, [α]20 D = 6 (*c* = 0.2, in acetone).; HRMS (ESI) m/z calcd for $C_{15}H_{18}N_3O_4Cl$, $[M+Na]^+$: 362.0884, found: 362.0878.

***tert*-butyl (*S*)-(4-hydroxy-1-(4-methoxyphenyl)-3-methyl-5-oxo-4,5-dihydro-1H-pyrazol-4-yl)carbamate 2d**

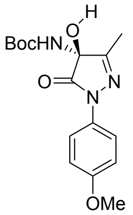

White soild, yield 53 %, ^{1}H NMR (400 MHz, $CDCl_3$) δ = 7.76 (d, *J*=9.0, 2H), 6.90 (d, *J*=9.1, 2H), 6.00 (s, 1H), 3.80 (s, 3H), 2.18 (s, 3H), 1.34 (s, 9H); ^{13}C NMR (101 MHz, $CDCl_3$) δ = 169.3, 159.5, 157.2, 153.3, 130.9, 120.6, 114.0, 82.7, 55.5, 28.1, 12.4.

Enantiometric excess of the product was determined by chiral stationary phase HPLC analysis using Daicel AD-H column (n-Hexane/i-PrOH 80:20 at 1.0 ml/min, λ = 230 nm, t_{minor} = 4.7 min, t_{major} = 7.1 min, 89% *ee*, [α]20 D = 3 (c = 0.2, in acetone).; HRMS (ESI) m/z calcd for $C_{16}H_{21}N_3O_5$, $[M+Na]^+$: 358.1379, found: 358.1359.

***tert*-butyl (*S*)-(1-(4-cyanophenyl)-4-hydroxy-3-methyl-5-oxo-4,5-dihydro-1H-pyrazol-4-yl)carbamate 2e**

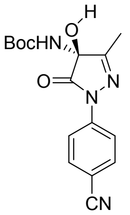

White soild, yield 58 %, ^{1}H NMR (700 MHz, $CDCl_3$) δ = 8.08 (d, *J*=8.3, 2H), 7.67 (d, *J*=8.3, 2H), 5.72 (s, 1H), 2.22 (s, 3H), 1.33 (s, 9H); ^{13}C NMR (101 MHz, $CDCl_3$) δ = 170.0, 160.0, 153.2, 141.0, 133.2, 118.7, 118.3, 108.2, 82.5, 28.0, 12.4.

Enantiometric excess of the product was determined by chiral stationary phase HPLC analysis using Daicel AD-H column (*n*-Hexane/*i*-PrOH 80:20 at 1.0 ml/min, λ = 230 nm, t_{minor} = 7.51 min, t_{major} = 10.8 min, 81% *ee*, [α]20 D = 15 (*c* = 0.2, in acetone).; HRMS (ESI) m/z calcd for $C_{16}H_{18}N_4O_4$, $[M+Na]^+$: 353.1226, found:353.1224.

***tert*-butyl (*S*)-(1-(4-fluorophenyl)-4-hydroxy-3-methyl-5-oxo-4,5-dihydro-1H-pyrazol-4-yl)carbamate 2f**

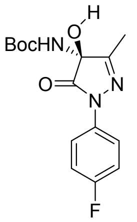

White soild, yield 59 %, ^{1}H NMR (400 MHz, $CDCl_3$) δ = 7.85 (dd, *J*=9.0, 4.7, 2H), 7.07 (dd, *J*=8.7, 8.7, 2H), 5.96 (s, 1H), 2.19 (s, 3H), 1.34 (s, 9H); ^{13}C NMR (101 MHz, $CDCl_3$) δ = 169.5, 159.9 (d, J_1 = 245.8 Hz), 159.5, 153.3, 133.6, 120.5, 115.7 (d, J_2 = 30.3), 82.7, 28.0, 12.4.

Enantiometric excess of the product was determined by chiral stationary phase HPLC analysis using Daicel AD-H column (*n*-Hexane/*i*-PrOH 80:20 at 1.0 ml/min, λ = 230 nm, t_{minor} = 5.3 min, t_{major} = 7.7 min, 86% *ee*, [α]20 D = -24.5 (*c* = 0.2, in MeOH).; HRMS (ESI) m/z calcd for $C_{15}H_{18}N_3O_4F$, $[M+H]^+$: 346.1179, found:346.1185.

***tert*-butyl (*S*)-(4-hydroxy-3-methyl-5-oxo-1-(4-(trifluoromethyl)phenyl)-4,5-dihydro-1H-pyrazol-4-yl) carbamate 2g**

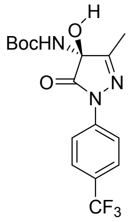

White soild, yield 67 %, ^{1}H NMR (400 MHz, $CDCl_3$) δ = 8.05 (d, *J*=8.6, 2H), 7.64 (d, *J*=8.7, 2H), 5.70 (s, 1H), 2.22 (s, 3H), 1.33 (s, 9H); ^{13}C NMR (101 MHz, $CDCl_3$) δ = 170.0, 160.0, 153.3, 140.2, 127.1 (q, J_2 = 30.3), 126.2 (q, J_3 = 4.0), 124.5 (q, J_1 = 272.7), 118.2, 82.6, 28.0, 12.4.

Enantiometric excess of the product was determined by chiral stationary phase HPLC analysis using Daicel AD-H column (*n*-Hexane/*i*-PrOH 80:20 at 1.0 ml/min, λ = 230 nm, t_{minor} = 4.9 min, t_{major} = 7.0 min, 81% *ee*, [α]20 D = 16.5 (*c* = 0.2, in MeOH).; HRMS (ESI) m/z calcd for $C_{16}H_{18}N_3O_4F_3$, $[M+Na]^+$: 396.1147, found:396.1265.

***tert*-butyl (S)-(1-(4-bromophenyl)-4-hydroxy-3-methyl-5-oxo-4,5-dihydro-1H-pyrazol-4-yl)carbamate 2h**

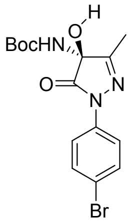

White soild, yield 60 %, ^{1}H NMR (400 MHz, $CDCl_3$) δ = 7.80 (d, *J*=8.9, 2H), 7.50 (d, *J*=8.9, 2H), 5.78 (s, 1H), 2.20 (s, 3H), 1.33 (s, 9H); ^{13}C NMR (101 MHz, $CDCl_3$) δ = 169.7, 159.6, 153.3, 136.6, 131.9, 120.1, 118.4, 82.7, 28.0, 12.4.

Enantiometric excess of the product was determined by chiral stationary phase HPLC analysis using Daicel AD-H column (*n*-Hexane/*i*-PrOH 90:10 at 0.8 ml/min, λ = 230 nm, t_{minor} = 12.3 min, t_{major} = 23.6 min, 81% *ee*, [α]20 D = -24 (*c* = 0.2, in MeOH).; HRMS (ESI) m/z calcd for $C_{15}H_{18}N_3O_4Br$, $[M+Na]^+$:406.0378, found:406.0375.

***tert*-butyl (*S*)-(4-hydroxy-3-isopropyl-5-oxo-1-phenyl-4,5-dihydro-1H-pyrazol-4-yl)carbamate 2i**

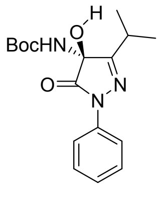

White soild, yield 55 %, ^{1}H NMR (400 MHz, $CDCl_3$) δ = 7.92 (d, *J*=8.1, 2H), 7.39 (t, *J*=7.8, 2H), 7.19 (t, *J*=7.3, 1H), 6.11 (s, 1H), 2.92 (dt, *J*=13.7, 6.9, 1H), 1.55-1.05 (m, 15H); ^{13}C NMR (101 MHz, $CDCl_3$) δ = 170.3, 165.6, 153.3, 137.8, 128.9, 125.4, 118.8, 83.6, 28.1, 27.9, 20.3, 20.0.

Enantiometric excess of the product was determined by chiral stationary phase HPLC analysis using Daicel AD-H column (*n*-Hexane/*i*-PrOH 93:7 at 0.8 ml/min, λ = 230 nm, t_{minor} = 14.7 min, t_{major} = 19.0 min, 85% *ee*, [α]20 D = 29.50 (*c* = 0.2, in acetone).; HRMS (ESI) *m/z* calcd for $C_{17}H_{23}N_3O_4$, $[M+Na]^+$: 356.1586, found:356.1603.

***tert*-butyl (*S*)-(4-hydroxy-5-oxo-1-phenyl-3-propyl-4,5-dihydro-1H-pyrazol-4-yl)carbamate 2j**

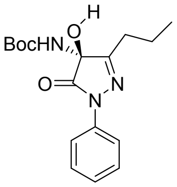

White soild, yield 56 %, ^{1}H NMR (400 MHz, $CDCl_3$) δ = 7.90 (d, *J*=7.8, 2H), 7.38 (t, *J*=7.7, 2H), 7.18 (t, *J*=7.2, 1H), 6.14 (s, 1H), 2.62-2.39 (m, 2H), 1.82 (dd, *J*=14.4, 7.2, 2H), 1.32 (s, 9H), 1.06 (t, *J*=7.3, 3H); ^{13}C NMR (101 MHz, $CDCl_3$) δ = 170.1, 162.0, 153.3, 137.7, 128.9, 125.3, 118.7, 83.2, 28.8, 28.0, 18.7, 14.0.

Enantiometric excess of the product was determined by chiral stationary phase HPLC analysis using Daicel AD-H column (*n*-Hexane/*i*-PrOH 80:20 at 1.0 ml/min, λ = 230 nm, t_{minor} = 8.9 min, t_{major} = 12.5 min, 82% *ee*, [α]20 D = 56.60 (*c* = 0.2, in MeOH).; HRMS (ESI) *m/z* calcd for $C_{17}H_{23}N_3O_4$, $[M+Na]^+$: 356.1586, found:356.1604.

***tert*-butyl (*S*)-(3-ethyl-4-hydroxy-5-oxo-1-phenyl-4,5-dihydro-1H-pyrazol-4-yl)carbamate 2k**

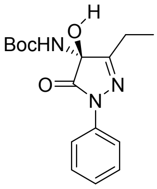

White soild, yield 63 %, ^{1}H NMR (400 MHz, $CDCl_3$) δ = 7.91 (d, *J*=7.7, 2H), 7.39 (d, *J*=7.7, 2H), 7.18 (t, *J*=7.4, 1H), 6.21 (s, 1H), 2.71-2.42 (m, 2H), 1.40-1.15 (m, 12H); ^{13}C NMR (101 MHz, $CDCl_3$) δ = 170.2, 163.1, 153.3, 137.7, 128.9, 125.36, 118.8, 83.1, 28.0, 20.23, 9.4.

Enantiometric excess of the product was determined by chiral stationary phase HPLC analysis using Daicel AD-H column (*n*-Hexane/*i*-PrOH 93:7 at 0.8 ml/min, λ = 230 nm, t_{minor} = 15.2 min, t_{major} = 23.2 min, 91% *ee*, [α]20 D = 16.50 (*c* = 0.2, in MeOH).; HRMS (ESI) m/z calcd for $C_{16}H_{22}N_3O_4$, $[M+Na]^+$: 342.1430, found: 342.1445.

***tert*-butyl (*S*)-(4-hydroperoxy-3-methyl-5-oxo-1-phenyl-4,5-dihydro-1H-pyrazol-4-yl)carbamate 3a**

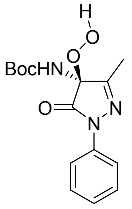

White soild, yield 62 %, ^{1}H NMR (400 MHz, $CDCl_3$) δ = 7.88 (d, *J*=7.8, 2H), 7.39 (t, *J*=7.4, 2H), 7.19 (t, *J*=7.0, 1H), 5.79 (s, 1H), 2.21 (s, 3H), 1.36 (s, 9H); ^{13}C NMR (101 MHz, $CDCl_3$) δ = 167.0, 156.7, 152.9, 137.3, 128.9, 125.7, 118.9, 89.6, 28.0, 14.2.

Enantiometric excess of the product was determined by chiral stationary phase HPLC analysis using Daicel AD-H column (*n*-Hexane/*i*-PrOH 80:20 at 1.0 ml/min, λ = 230 nm, t_{major} = 5.5 min, t_{minor} = 8.7 min, 86% *ee*, [α]20 D = -48.50 (*c* = 0.4, in MeOH).; HRMS (ESI) *m/z* calcd for $C_{15}H_{19}N_3O_5$, $[M+Na]^+$: 344.1222, found:344.1259.

***tert*-butyl (*S*)-(3-ethyl-4-hydroperoxy-5-oxo-1-phenyl-4,5-dihydro-1H-pyrazol-4-yl)carbamate 3b**

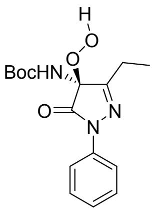

White soild, yield 60 %, ^{1}H NMR (400 MHz, $CDCl_3$) δ = 7.91 (d, *J*=7.8, 2H), 7.39 (t, *J*=8.0, 2H), 7.19 (t, *J*=7.4, 1H), 5.98 (s, 2H), 2.67-2.46 (m, 2H), 1.33 (t, *J*=7.4, 12H); ^{13}C NMR (101 MHz, $CDCl_3$) δ = 167.3, 160.0, 152.8, 137.4, 128.9, 125.6, 118.8, 89.7, 28.0, 21.6, 9.4.

Enantiometric excess of the product was determined by chiral stationary phase HPLC analysis using Daicel AD-H column (*n*-Hexane/*i*-PrOH 80:20 at 1.0 ml/min, λ = 230 nm, t_{major} = 5.2 min, t_{minor} = 8.3 min, 80% *ee*, [α]20 D = -29.75 (*c* = 0.4, in MeOH).; HRMS (ESI) *m/z* calcd for $C_{16}H_{21}N_3O_5$, $[M+Na]^+$: 358.1379, found:358.1374.

***tert*-butyl (*S*)-(4-hydroperoxy-5-oxo-1-phenyl-3-propyl-4,5-dihydro-1H-pyrazol-4-yl)carbamate 3c**

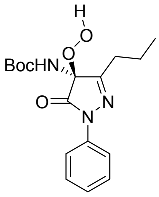

White soild, yield 61 %, ^{1}H NMR (400 MHz, $CDCl_3$) δ = 10.72 (s, 1H), 7.89 (d, *J*=8.0, 2H), 7.39 (t, *J*=7.9, 2H), 7.20 (t, *J*=7.3, 1H), 6.57 (s, 1H), 2.54 (ddd, *J*=16.3, 9.9, 6.2, 2H), 1.90-1.88 (m, 2H), 1.34 (s, 9H), 1.07 (t, *J*=7.4, 3H); ^{13}C NMR (101 MHz, $CDCl_3$) δ = 167.51, 159.35, 153.19, 137.43, 128.90, 125.57, 118.83, 90.21, 29.71, 27.99, 18.64, 13.94.

Enantiometric excess of the product was determined by chiral stationary phase HPLC analysis using Daicel AD-H column (n-Hexane/i-PrOH 95:5 at 0.9 ml/min, λ = 230 nm, t_{major} = 18.1 min, t_{minor} = 35.31 min, 88% *ee*, [α]20 D = -22.50 (c = 0.4, in MeOH).; HRMS (ESI) *m/z* calcd for $C_{17}H_{23}N_3O_5$, [M+Na]+ : 372.1535, found:372.1609.

***tert*-butyl (*S*)-(4-hydroperoxy-3-isopropyl-5-oxo-1-phenyl-4,5-dihydro-1H-pyrazol-4-yl)carbamate 3d**

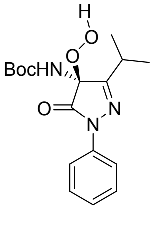

White soild, yield 60 %, ^{1}H NMR (400 MHz, $CDCl_3$) δ = 10.80 (s, 1H), 7.88 (t, *J*=7.7, 2H), 7.37 (t, *J*=8.0, 2H), 7.18 (dd, *J*=9.2, 4.4, 1H), 6.31 (s, 1H), 3.00-2.85 (m, 1H), 1.40-1.20 (m, 15H); ^{13}C NMR (101 MHz, $CDCl_3$) δ = 167.6, 162.3, 152.8, 137.5, 128.9, 125.5, 118.8, 90.3, 28.3, 28.0, 20.0, 19.9.

Enantiometric excess of the product was determined by chiral stationary phase HPLC analysis using Daicel AD-H column (*n*-Hexane/*i*-PrOH 80:20 at 1.0 ml/min, λ = 230 nm, t_{major} = 5.1 min, t_{minor} = 7.5 min, 88% *ee*, [α]20 D = -34.00 (*c* = 0.4, in MeOH).; HRMS (ESI) *m/z* calcd for $C_{17}H_{23}N_3O_5$, $[M+Na]^+$: 372.1535, found:372.1555.

***tert*-butyl (*S*)-(4-hydroperoxy-3-methyl-1-(naphthalen-1-yl)-5-oxo-4,5-dihydro-1H-pyrazol-4-yl)carbamate 3e**

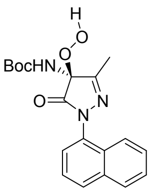

White soild, yield 47 %, ^{1}H NMR (400 MHz, $CDCl_3$) δ = 10.43 (s, 1H), 8.33 (d, *J*=7.9, 1H), 8.06 (td, *J*=9.0, 2.0, 1H), 7.90-7.78 (m, 3H), 7.52-7.40 (m, 2H), 6.24 (s, 1H), 2.32 (s, 3H), 1.38 (s, 9H); ^{13}C NMR (101 MHz, $CDCl_3$) δ = 167.2, 156.9, 152.9, 134.8, 133.4, 131.3, 128.9, 128., 127.6, 126.6, 125.7, 118.1, 116.3, 89.7, 28.0, 14.2.

Enantiometric excess of the product was determined by chiral stationary phase HPLC analysis using Daicel AD-H column (*n*-Hexane/*i*-PrOH 80:20 at 1.0 ml/min, λ = 230 nm, t_{major} = 8.7 min, t_{minor} = 22.0 min, 70% *ee,* [α]20 D = -27.25 (*c* = 0.4, in MeOH).; HRMS (ESI) *m/z* calcd for $C_{16}H_{21}N_3O_5$, $[M+NH_4]^+$: 389.1825, found:389.1819.

***tert*-butyl (*S*)-(1-(4-chlorophenyl)-4-hydroperoxy-3-methyl-5-oxo-4,5-dihydro-1H-pyrazol-4-yl)carbamate 3f**

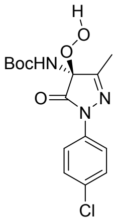

White soild, yield 58 %, ^{1}H NMR (400 MHz, $CDCl_3$) δ = 10.52 (s, 1H), 7.83 (d, *J*=9.0, 2H), 7.35 (d, *J*=8.9, 2H), 6.19 (s, 1H), 2.26 (s, 3H), 1.36 (s, 9H); ^{13}C NMR (101 MHz, $CDCl_3$) δ = 167.0, 156.9, 152.9, 135.9, 130.8, 129.0, 119.9, 89.6, 28.0, 14.1.

Enantiometric excess of the product was determined by chiral stationary phase HPLC analysis using Daicel AD-H column (*n*-Hexane/*i*-PrOH 90:10 at 0.9 ml/min, λ = 230 nm, t_{major} = 3.4 min, t_{minor} = 6.4 min, 80% *ee*, [α]20 D = -19.75 (*c* = 0.4, in MeOH).; HRMS (ESI) *m/z* calcd for $C_{15}H_{18}N_3O_5Cl$, $[M+Na]^+$: 378.0833, found: 378.0825.

***tert*-butyl (*S*)-(4-hydroperoxy-1-(4-methoxyphenyl)-3-methyl-5-oxo-4,5-dihydro-1H-pyrazol-4-yl)carbamate 3g**

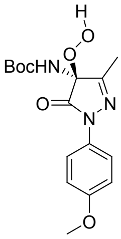

White soild, yield 48 %, ^{1}H NMR (400 MHz, $CDCl_3$) δ = 10.51 (s, 1H), 7.72 (d, *J*=9.1, 2H), 6.90 (d, *J*=9.2, 2H), 6.11 (s, 1H), 3.81 (s, 3H), 2.27 (s, 3H), 1.39 (s, 9H); ^{13}C NMR (101 MHz, $CDCl_3$) δ = 166.7, 157.5, 156.8, 152.8, 130.5, 120.8, 114.1, 89.6, 55.5, 28.0, 14.2.

Enantiometric excess of the product was determined by chiral stationary phase HPLC analysis using Daicel AD-H column (*n*-Hexane/*i*-PrOH 80:20 at 1.0 ml/min, λ = 230 nm, t_{major} = 7.8 min, t_{minor} = 16.0 min, 81% *ee*, [α]20 D = -13.00 (*c* = 0.4, in MeOH).; HRMS (ESI) *m/z* calcd for $C_{16}H_{22}N_3O_6$, $[M+NH_4]^+$: 369.1774, found:369.1770.

3. X-ray crystallographic data of 2h

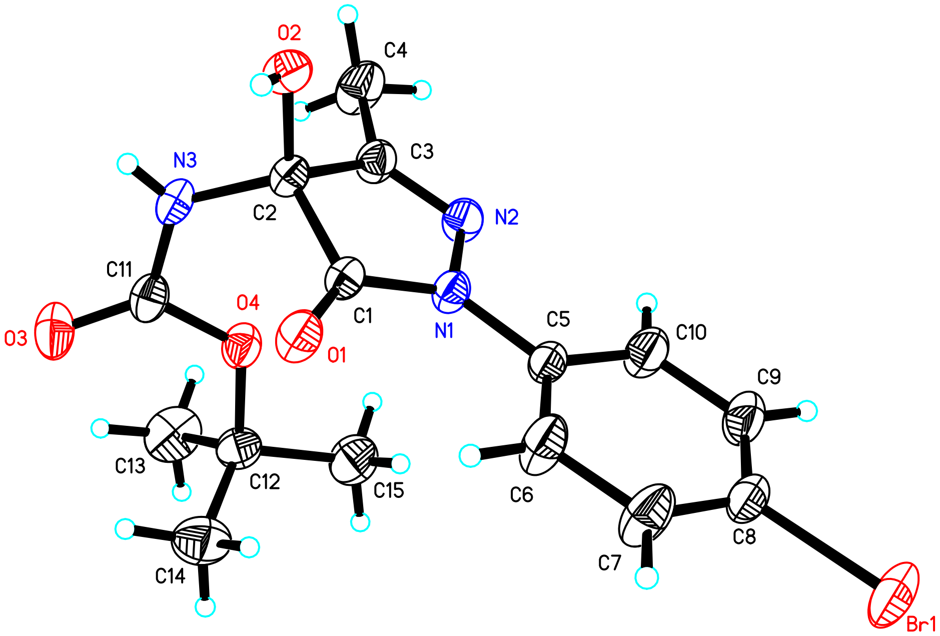

Figure S1. X-ray crystallographic data of 2h

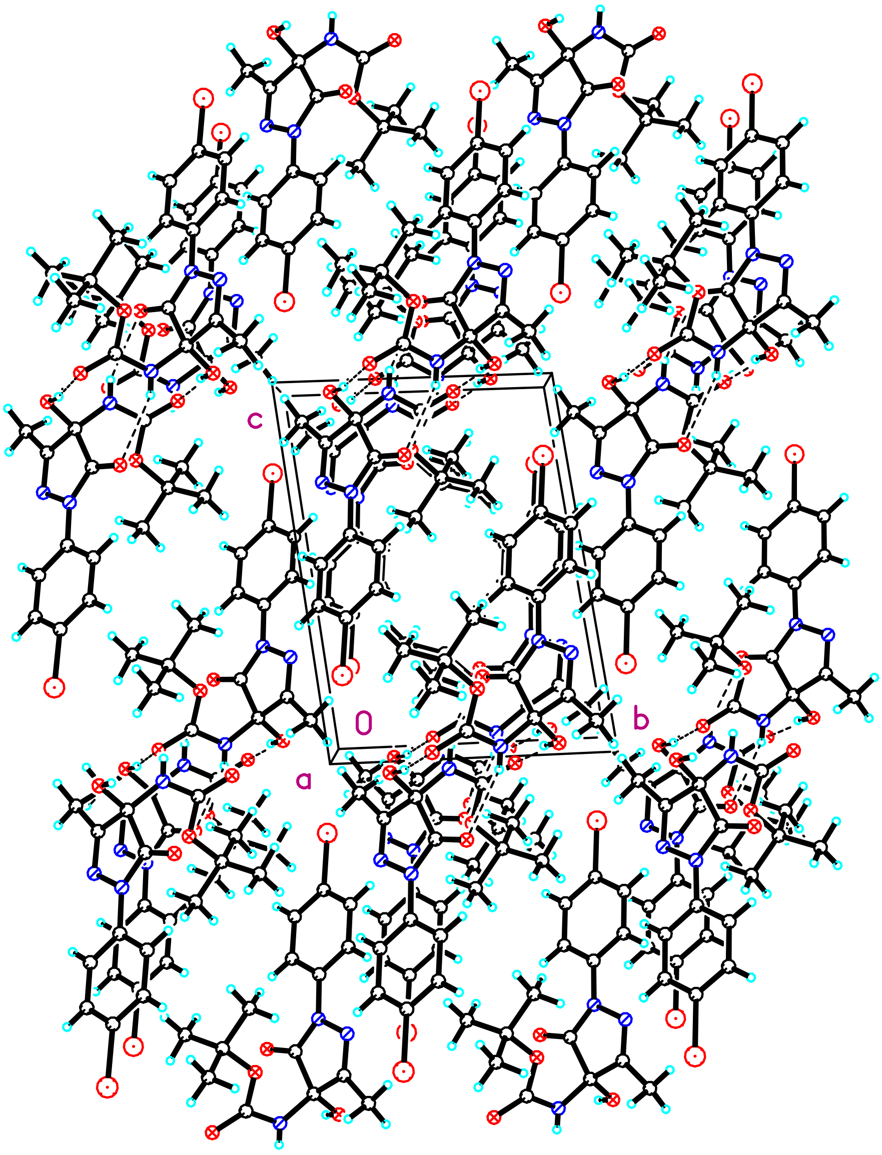

Figure S2. X-ray crystallographic data of 2h in unit cell.

Table S1. Crystal data and structure refinement for mo_d8v18375_0m.

Identification code	mo_d8v18375_0m
Empirical formula	C15 H18 Br N3 O4
Formula weight	384.23
Temperature	296(2) K
Wavelength	0.71073 Å
Crystal system	Triclinic
Space group	P -1
Unit cell dimensions	a = 8.4388(7) Å
a= 95.483(3)°.	
	b = 8.6480(8) Å
b= 104.562(2)°.	
	c = 12.0506(10) Å
g = 95.203(3)°.	
Volume	841.27(13) $Å^3$
Z	2
Density (calculated)	1.517 Mg/m^3
Absorption coefficient	2.466 mm^{-1}
F(000)	392
Crystal size	0.200 x 0.160 x 0.120 mm^3
Theta range for data collection	2.383 to 25.998°.
Index ranges	-10<=h<=10, -10<=k<=10, -14<=l<=14
Reflections collected	14416
Independent reflections	3308 [R(int) = 0.0413]
Completeness to theta = 25.242°	99.7 %
Absorption correction	Semi-empirical from equivalents
Max. and min. transmission	0.7456 and 0.4850
Refinement method	Full-matrix least-squares on F^2
Data / restraints / parameters	3308 / 0 / 217
Goodness-of-fit on F^2	1.036
Final R indices [I>2sigma(I)]	R1 = 0.0362, wR2 = 0.0840
R indices (all data)	R1 = 0.0509, wR2 = 0.0921
Extinction coefficient	n/a
Largest diff. peak and hole	0.700 and -0.647 $e.Å^{-3}$

Table S2. Atomic coordinates (x 10^4) and equivalent isotropic displacement parameters (Å^{2}x 10^3) for mo_d8v18375_0m. U(eq) is defined as one third of the trace of the orthogonalized U^{ij} tensor.

	x	y	z	U(eq)
Br(1)	883(1)	9142(1)	7771(1)	75(1)
O(1)	-77(2)	5736(2)	2057(2)	50(1)
O(2)	277(2)	7997(2)	296(2)	52(1)
O(3)	2873(3)	3601(2)	298(2)	62(1)
O(4)	3730(2)	5468(2)	1853(2)	49(1)
N(1)	1843(2)	7859(2)	2958(2)	39(1)
N(2)	3053(3)	8864(2)	2646(2)	45(1)
N(3)	1884(3)	5950(3)	328(2)	44(1)
C(1)	954(3)	6809(3)	2057(2)	38(1)
C(2)	1502(3)	7277(3)	998(2)	40(1)
C(3)	2917(3)	8523(3)	1576(2)	46(1)
C(4)	4013(4)	9294(4)	949(3)	70(1)
C(5)	1664(3)	8118(3)	4097(2)	39(1)
C(6)	542(4)	7158(4)	4452(2)	62(1)
C(7)	326(4)	7468(4)	5548(3)	68(1)
C(8)	1224(4)	8715(3)	6282(2)	49(1)
C(9)	2366(5)	9645(4)	5946(2)	66(1)
C(10)	2585(4)	9353(4)	4849(2)	60(1)
C(11)	2835(3)	4886(3)	791(2)	46(1)
C(12)	4828(3)	4535(3)	2606(2)	51(1)
C(13)	6112(4)	4019(5)	2031(3)	78(1)
C(14)	3800(5)	3207(4)	2902(3)	82(1)
C(15)	5611(5)	5725(4)	3657(3)	76(1)

Table S3. Bond lengths [Å] and angles [°] for mo_d8v18375_0m.

Br(1)-C(8)	1.895(2)
O(1)-C(1)	1.213(3)
O(2)-C(2)	1.396(3)
O(2)-H(2)	0.82
O(3)-C(11)	1.216(3)
O(4)-C(11)	1.336(3)
O(4)-C(12)	1.478(3)
N(1)-C(1)	1.358(3)
N(1)-C(5)	1.416(3)
N(1)-N(2)	1.427(3)
N(2)-C(3)	1.268(3)
N(3)-C(11)	1.345(4)
N(3)-C(2)	1.447(3)
N(3)-H(3)	0.74(3)
C(1)-C(2)	1.538(3)
C(2)-C(3)	1.509(4)
C(3)-C(4)	1.485(4)
C(4)-H(4A)	0.96
C(4)-H(4B)	0.96
C(4)-H(4C)	0.96
C(5)-C(10)	1.371(4)
C(5)-C(6)	1.378(4)
C(6)-C(7)	1.383(4)
C(6)-H(6)	0.93
C(7)-C(8)	1.361(4)
C(7)-H(7)	0.93
C(8)-C(9)	1.363(4)
C(9)-C(10)	1.384(4)
C(9)-H(9)	0.93

C(10)-H(10)	0.93
C(12)-C(14)	1.501(4)
C(12)-C(13)	1.503(4)
C(12)-C(15)	1.519(4)
C(13)-H(13A)	0.96
C(13)-H(13B)	0.96
C(13)-H(13C)	0.96
C(14)-H(14A)	0.96
C(14)-H(14B)	0.96
C(14)-H(14C)	0.96
C(15)-H(15A)	0.96
C(15)-H(15B)	0.96
C(15)-H(15C)	0.96
C(2)-O(2)-H(2)	109.5
C(11)-O(4)-C(12)	122.2(2)
C(1)-N(1)-C(5)	129.2(2)
C(1)-N(1)-N(2)	112.35(19)
C(5)-N(1)-N(2)	118.31(19)
C(3)-N(2)-N(1)	108.2(2)
C(11)-N(3)-C(2)	123.8(2)
C(11)-N(3)-H(3)	119(2)
C(2)-N(3)-H(3)	115(2)
O(1)-C(1)-N(1)	128.5(2)
O(1)-C(1)-C(2)	126.1(2)
N(1)-C(1)-C(2)	105.4(2)
O(2)-C(2)-N(3)	109.6(2)
O(2)-C(2)-C(3)	107.6(2)
N(3)-C(2)-C(3)	116.5(2)

O(2)-C(2)-C(1)	110.2(2)
N(3)-C(2)-C(1)	112.2(2)
C(3)-C(2)-C(1)	100.35(19)
N(2)-C(3)-C(4)	123.5(2)
N(2)-C(3)-C(2)	113.1(2)
C(4)-C(3)-C(2)	123.4(2)
C(3)-C(4)-H(4A)	109.5
C(3)-C(4)-H(4B)	109.5
H(4A)-C(4)-H(4B)	109.5
C(3)-C(4)-H(4C)	109.5
H(4A)-C(4)-H(4C)	109.5
H(4B)-C(4)-H(4C)	109.5
C(10)-C(5)-C(6)	119.3(2)
C(10)-C(5)-N(1)	120.0(2)
C(6)-C(5)-N(1)	120.7(2)
C(5)-C(6)-C(7)	120.0(3)
C(5)-C(6)-H(6)	120
C(7)-C(6)-H(6)	120
C(8)-C(7)-C(6)	120.2(3)
C(8)-C(7)-H(7)	119.9
C(6)-C(7)-H(7)	119.9
C(7)-C(8)-C(9)	120.0(3)
C(7)-C(8)-Br(1)	119.6(2)
C(9)-C(8)-Br(1)	120.4(2)
C(8)-C(9)-C(10)	120.3(3)
C(8)-C(9)-H(9)	119.9
C(10)-C(9)-H(9)	119.9
C(5)-C(10)-C(9)	120.1(3)
C(5)-C(10)-H(10)	119.9

C(9)-C(10)-H(10)	119.9
O(3)-C(11)-O(4)	126.0(3)
O(3)-C(11)-N(3)	124.3(2)
O(4)-C(11)-N(3)	109.7(2)
O(4)-C(12)-C(14)	109.0(2)
O(4)-C(12)-C(13)	109.8(2)
C(14)-C(12)-C(13)	113.7(3)
O(4)-C(12)-C(15)	101.7(2)
C(14)-C(12)-C(15)	111.4(3)
C(13)-C(12)-C(15)	110.6(3)
C(12)-C(13)-H(13A)	109.5
C(12)-C(13)-H(13B)	109.5
H(13A)-C(13)-H(13B)	109.5
C(12)-C(13)-H(13C)	109.5
H(13A)-C(13)-H(13C)	109.5
H(13B)-C(13)-H(13C)	109.5
C(12)-C(14)-H(14A)	109.5
C(12)-C(14)-H(14B)	109.5
H(14A)-C(14)-H(14B)	109.5
C(12)-C(14)-H(14C)	109.5
H(14A)-C(14)-H(14C)	109.5
H(14B)-C(14)-H(14C)	109.5
C(12)-C(15)-H(15A)	109.5
C(12)-C(15)-H(15B)	109.5
H(15A)-C(15)-H(15B)	109.5
C(12)-C(15)-H(15C)	109.5
H(15A)-C(15)-H(15C)	109.5
H(15B)-C(15)-H(15C)	109.5

Symmetry transformations used to generate equivalent atoms:

Table S4. Anisotropic displacement parameters (Å^{2}x 10^3) for mo_d8v18375_0m. The anisotropic displacement factor exponent takes the form: -2p^2[h^2 a*^{2}U^{11} + ... + 2 h k a* b* U^{12}]

	U11	U22	U33	U23	U13	U12
Br(1)	79(1)	114(1)	36(1)	1(1)	21(1)	31(1)
O(1)	51(1)	56(1)	40(1)	-1(1)	15(1)	-14(1)
O(2)	57(1)	56(1)	42(1)	11(1)	9(1)	10(1)
O(3)	61(1)	61(1)	55(1)	-15(1)	8(1)	13(1)
O(4)	48(1)	53(1)	40(1)	-2(1)	4(1)	9(1)
N(1)	42(1)	42(1)	32(1)	0(1)	13(1)	-4(1)
N(2)	49(1)	44(1)	42(1)	2(1)	17(1)	-7(1)
N(3)	45(1)	56(1)	29(1)	-3(1)	7(1)	5(1)
C(1)	39(1)	44(1)	32(1)	2(1)	11(1)	5(1)
C(2)	42(1)	45(1)	32(1)	4(1)	10(1)	3(1)
C(3)	52(2)	46(2)	40(1)	3(1)	17(1)	-4(1)
C(4)	81(2)	77(2)	52(2)	5(2)	30(2)	-25(2)
C(5)	42(1)	44(1)	32(1)	3(1)	10(1)	8(1)
C(6)	64(2)	75(2)	43(2)	-10(1)	23(1)	-20(2)
C(7)	63(2)	95(3)	46(2)	-1(2)	26(2)	-12(2)
C(8)	55(2)	64(2)	32(1)	4(1)	13(1)	21(1)
C(9)	91(2)	59(2)	40(2)	-10(1)	14(2)	-6(2)
C(10)	77(2)	58(2)	41(2)	-3(1)	19(1)	-14(2)
C(11)	41(2)	55(2)	40(1)	-4(1)	14(1)	2(1)
C(12)	47(2)	56(2)	48(2)	10(1)	9(1)	8(1)
C(13)	50(2)	109(3)	81(2)	15(2)	19(2)	26(2)
C(14)	94(3)	77(2)	80(2)	24(2)	34(2)	-6(2)
C(15)	80(2)	78(2)	55(2)	4(2)	-7(2)	11(2)

Table S5. Hydrogen coordinates (x 10^4) and isotropic displacement parameters (Å^{2}x 10^3) for mo_d8v18375_0m.

	x	y	z	U(eq)
H(2)	-622	7483	206	78
H(3)	1360(30)	5770(30)	-280(30)	43(8)
H(4A)	4784	10089	1471	105
H(4B)	3362	9760	325	105
H(4C)	4603	8530	647	105
H(6)	-71	6301	3954	74
H(7)	-436	6821	5785	82
H(9)	3000	10480	6456	79
H(10)	3360	9997	4621	72
H(13A)	5600	3254	1378	117
H(13B)	6929	3568	2571	117
H(13C)	6628	4905	1777	117
H(14A)	2990	3613	3247	122
H(14B)	4496	2642	3438	122
H(14C)	3257	2516	2212	122
H(15A)	6214	6584	3433	114
H(15B)	6350	5239	4225	114
H(15C)	4766	6104	3977	114

Table S6. Torsion angles [°] for mo_d8v18375_0m.

C(1)-N(1)-N(2)-C(3)	2.4(3)
C(5)-N(1)-N(2)-C(3)	-174.5(2)
C(5)-N(1)-C(1)-O(1)	-9.1(4)
N(2)-N(1)-C(1)-O(1)	174.5(2)
C(5)-N(1)-C(1)-C(2)	169.8(2)
N(2)-N(1)-C(1)-C(2)	-6.7(3)
C(11)-N(3)-C(2)-O(2)	-170.0(2)
C(11)-N(3)-C(2)-C(3)	67.6(3)
C(11)-N(3)-C(2)-C(1)	-47.3(3)
O(1)-C(1)-C(2)-O(2)	73.3(3)
N(1)-C(1)-C(2)-O(2)	-105.6(2)
O(1)-C(1)-C(2)-N(3)	-49.0(3)
N(1)-C(1)-C(2)-N(3)	132.1(2)
O(1)-C(1)-C(2)-C(3)	-173.4(2)
N(1)-C(1)-C(2)-C(3)	7.7(3)
N(1)-N(2)-C(3)-C(4)	-177.9(3)
N(1)-N(2)-C(3)-C(2)	3.3(3)
O(2)-C(2)-C(3)-N(2)	108.3(3)
N(3)-C(2)-C(3)-N(2)	-128.2(3)
C(1)-C(2)-C(3)-N(2)	-6.9(3)
O(2)-C(2)-C(3)-C(4)	-70.5(3)
N(3)-C(2)-C(3)-C(4)	53.0(4)
C(1)-C(2)-C(3)-C(4)	174.3(3)
C(1)-N(1)-C(5)-C(10)	-173.2(3)
N(2)-N(1)-C(5)-C(10)	3.0(4)
C(1)-N(1)-C(5)-C(6)	5.3(4)
N(2)-N(1)-C(5)-C(6)	-178.4(3)
C(10)-C(5)-C(6)-C(7)	1.6(5)
N(1)-C(5)-C(6)-C(7)	-177.0(3)

C(5)-C(6)-C(7)-C(8)	-0.3(5)
C(6)-C(7)-C(8)-C(9)	-1.4(5)
C(6)-C(7)-C(8)-Br(1)	178.9(3)
C(7)-C(8)-C(9)-C(10)	1.8(5)
Br(1)-C(8)-C(9)-C(10)	-178.5(3)
C(6)-C(5)-C(10)-C(9)	-1.1(5)
N(1)-C(5)-C(10)-C(9)	177.4(3)
C(8)-C(9)-C(10)-C(5)	-0.5(5)
C(12)-O(4)-C(11)-O(3)	-4.3(4)
C(12)-O(4)-C(11)-N(3)	176.5(2)
C(2)-N(3)-C(11)-O(3)	162.7(3)
C(2)-N(3)-C(11)-O(4)	-18.2(4)
C(11)-O(4)-C(12)-C(14)	-65.3(3)
C(11)-O(4)-C(12)-C(13)	59.8(3)
C(11)-O(4)-C(12)-C(15)	176.9(3)

Symmetry transformations used to generate equivalent atoms:

Table S7. Hydrogen bonds for mo_d8v18375_0m [Å and °].

D-H...A	d(D-H)	d(H...A)	d(D...A)	<(DHA)
C(14)-H(14C)...O(3)	0.96	2.53	3.098(4)	117.9
C(13)-H(13A)...O(3)	0.96	2.4	2.961(4)	116.7
C(6)-H(6)...O(1)	0.93	2.29	2.926(3)	125.1
N(3)-H(3)...O(1)#1	0.74(3)	2.35(3)	3.050(3)	158(3)
O(2)-H(2)...O(3)#1	0.82	1.96	2.770(3)	169.1

Symmetry transformations used to generate equivalent atoms:

#1 -x,-y+1,-z

4. ^{1}H NMR and ^{13}C NMR spectra

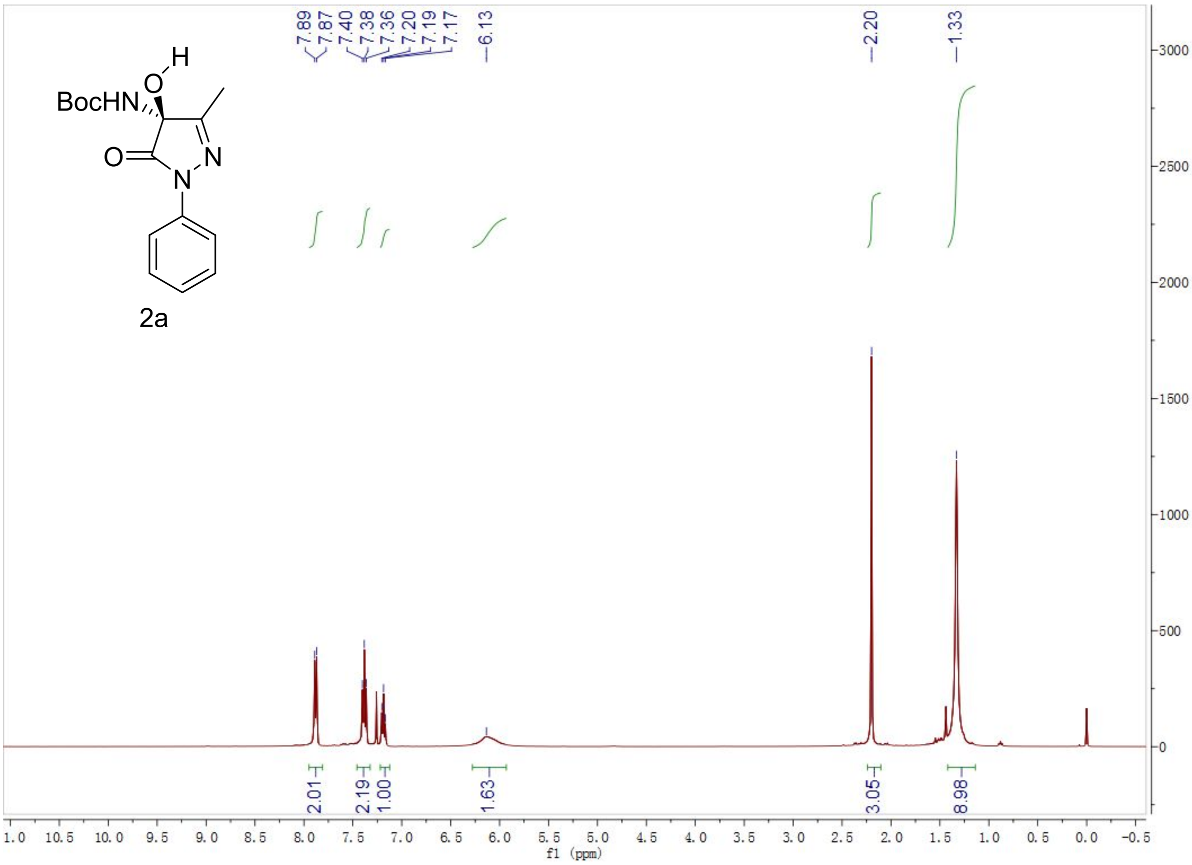

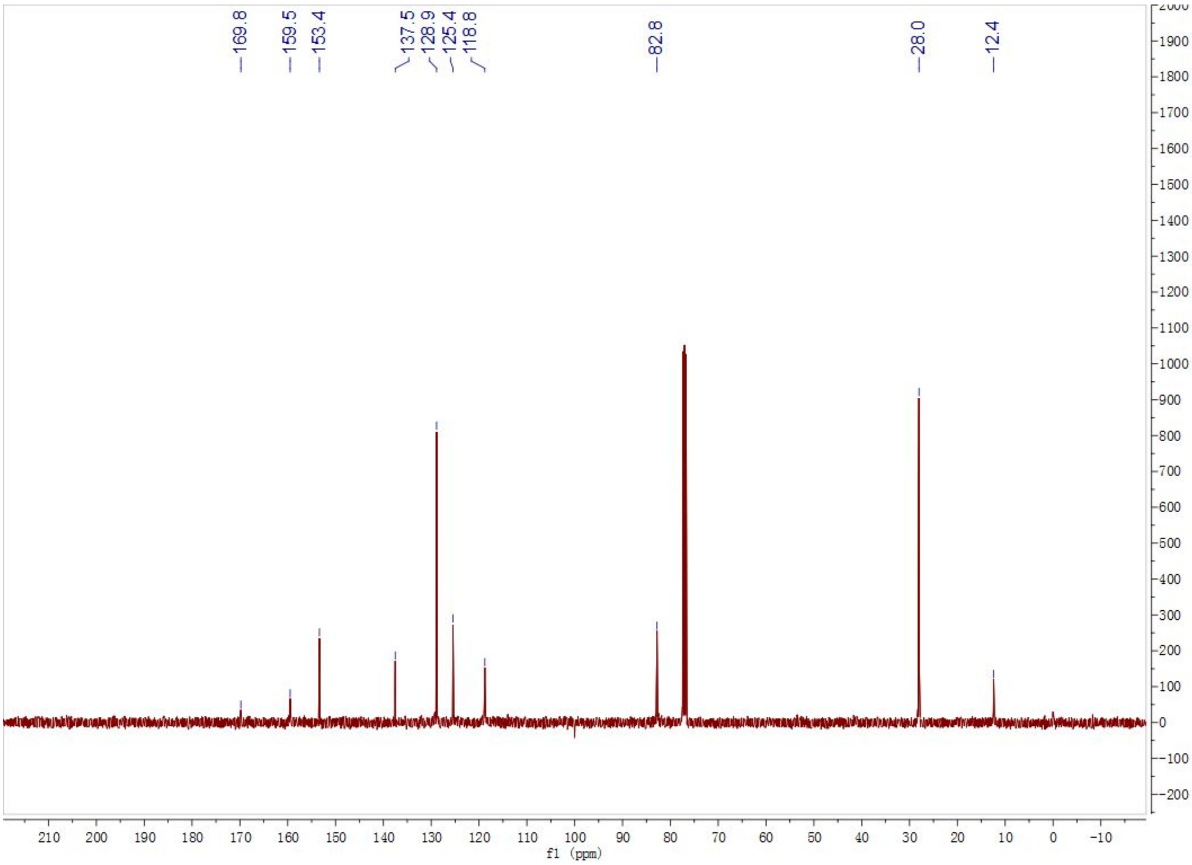

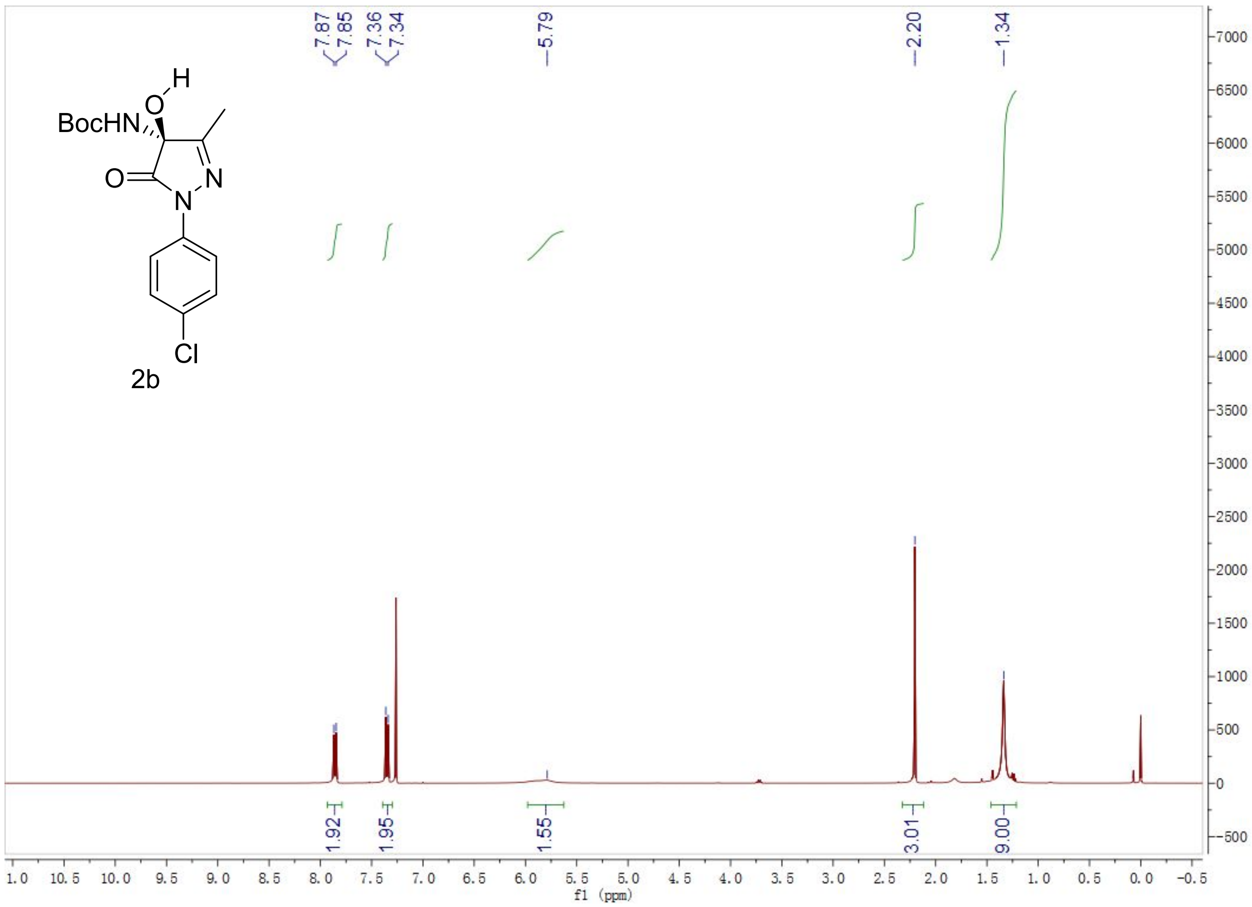
7.87
7.85
7.36
7.34
5.79
2.20
1.34
BocHN
O
H
N
N
Cl
2b
1.92
1.95
1.55
3.01
9.00
f1 (ppm)

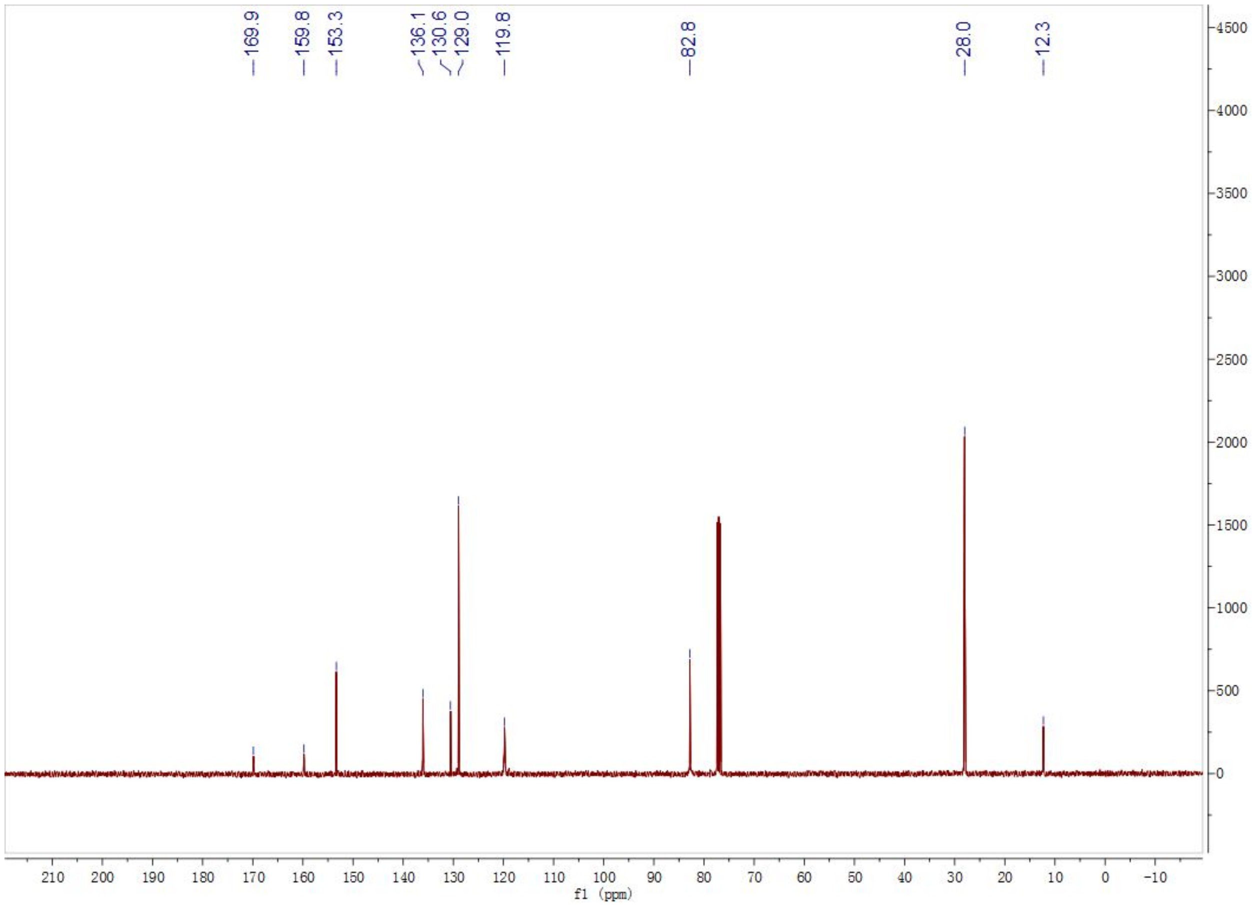
169.9
159.8
153.3
136.1
130.6
129.0
119.8
82.8
28.0
12.3
f1 (ppm)

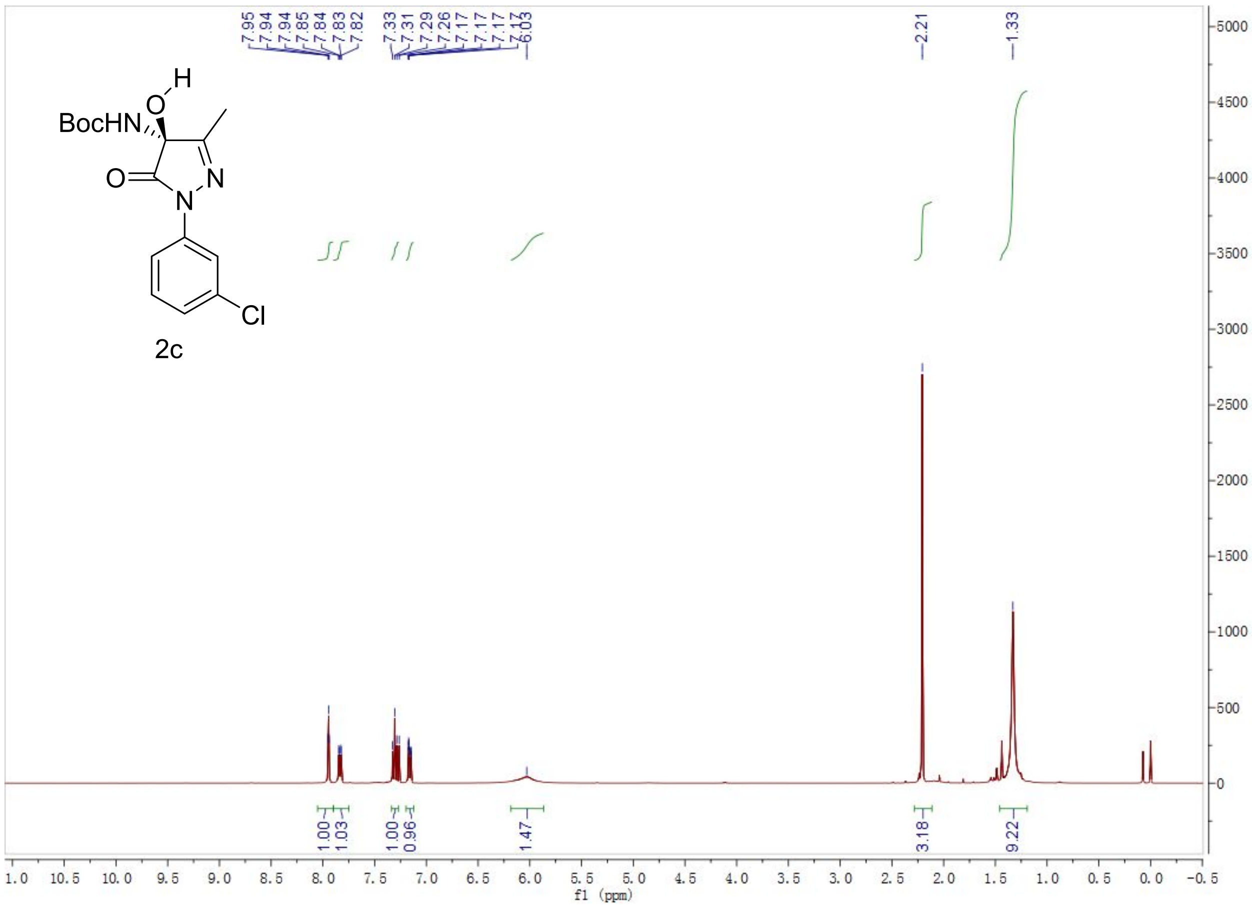
BocHN
O
H
N
N
O
Cl
2c
7.95
7.94
7.94
7.85
7.84
7.83
7.82
7.33
7.31
7.29
7.26
7.17
7.17
7.17
7.17
6.03
2.21
1.33
1.00
1.03
1.00
0.96
1.47
3.18
9.22
f1 (ppm)

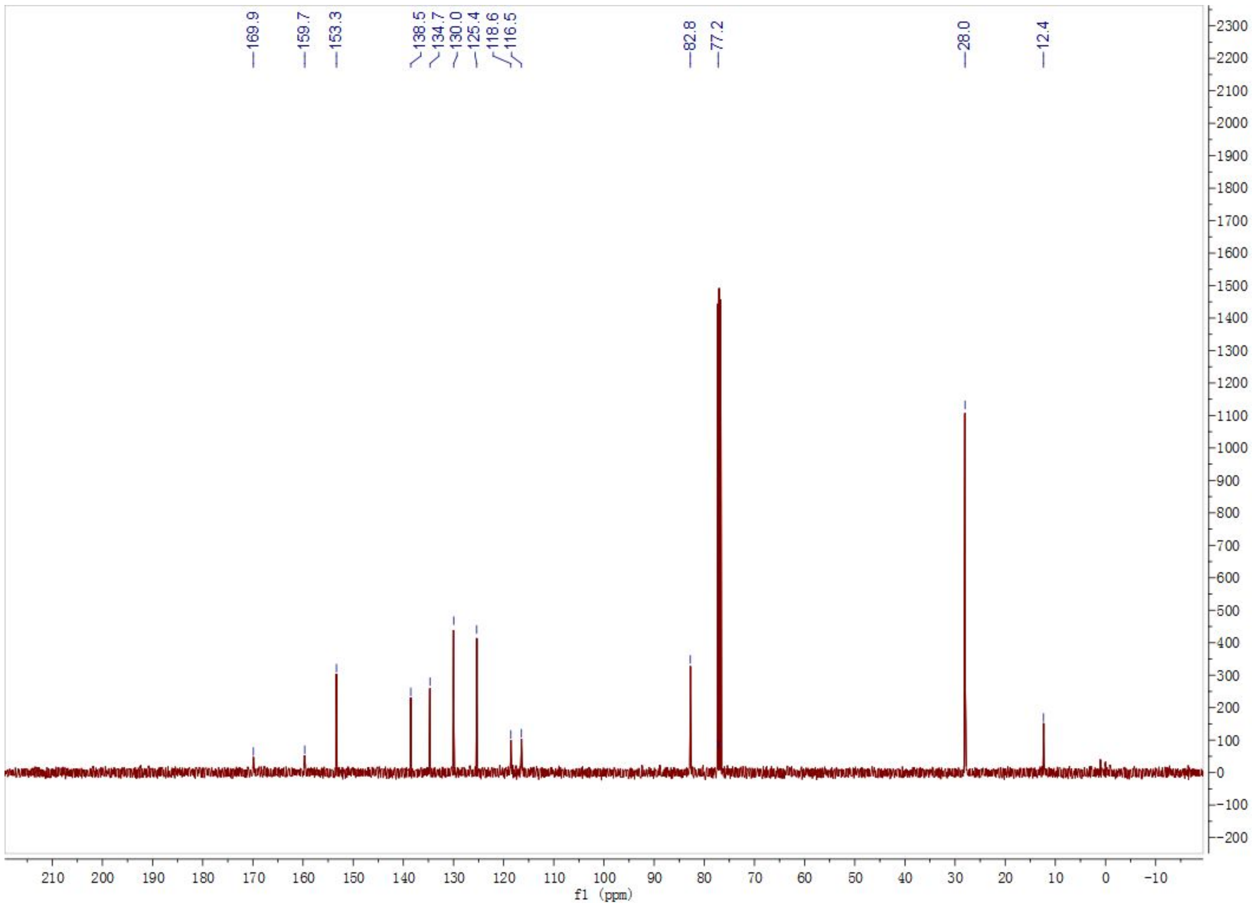
169.9
159.7
153.3
138.5
134.7
130.0
125.4
118.6
116.5
82.8
77.2
28.0
12.4
f1 (ppm)

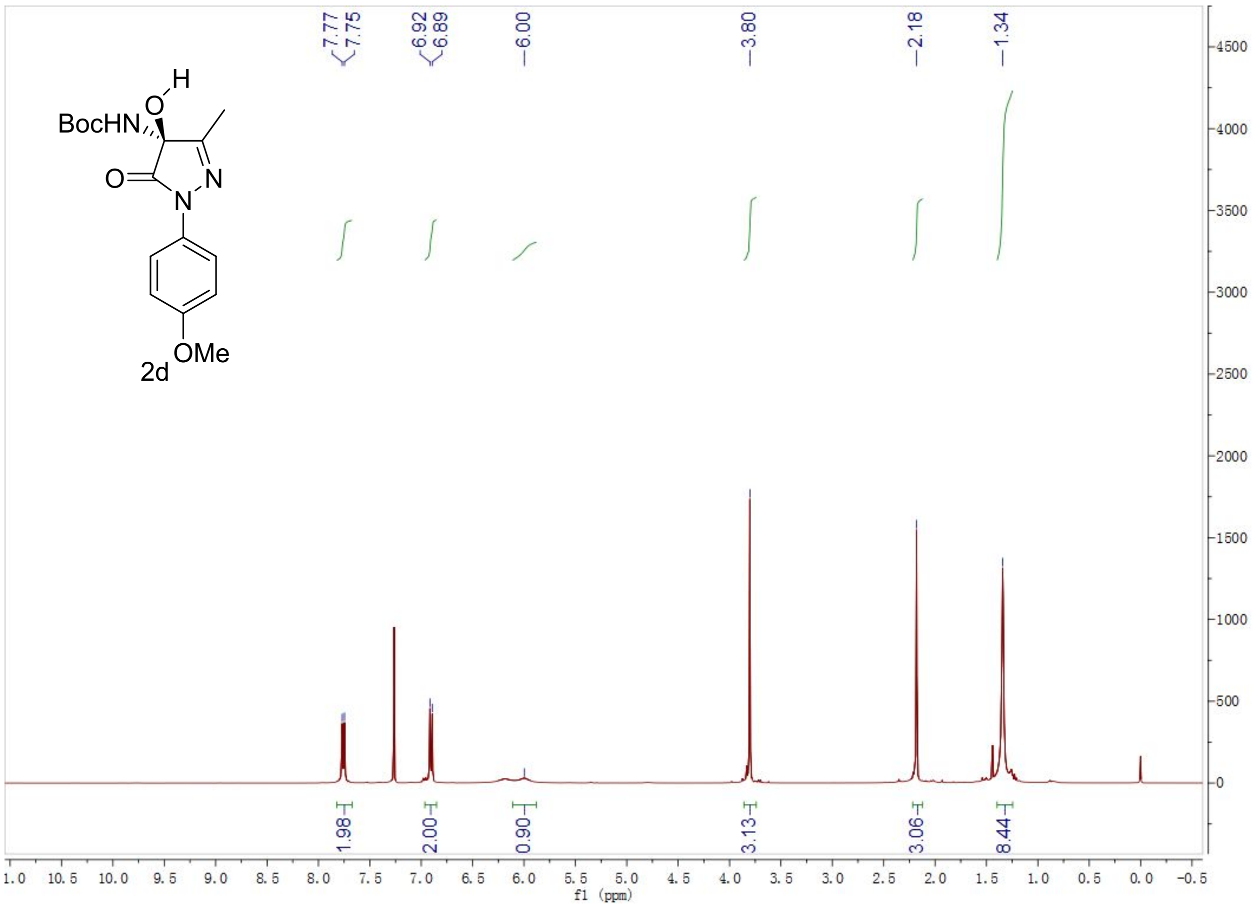
BocHN
O
H
O
N
N
OMe
2d
7.77
7.75
6.92
6.89
6.00
3.80
2.18
1.34
1.98
2.00
0.90
3.13
3.06
8.44
f1 (ppm)

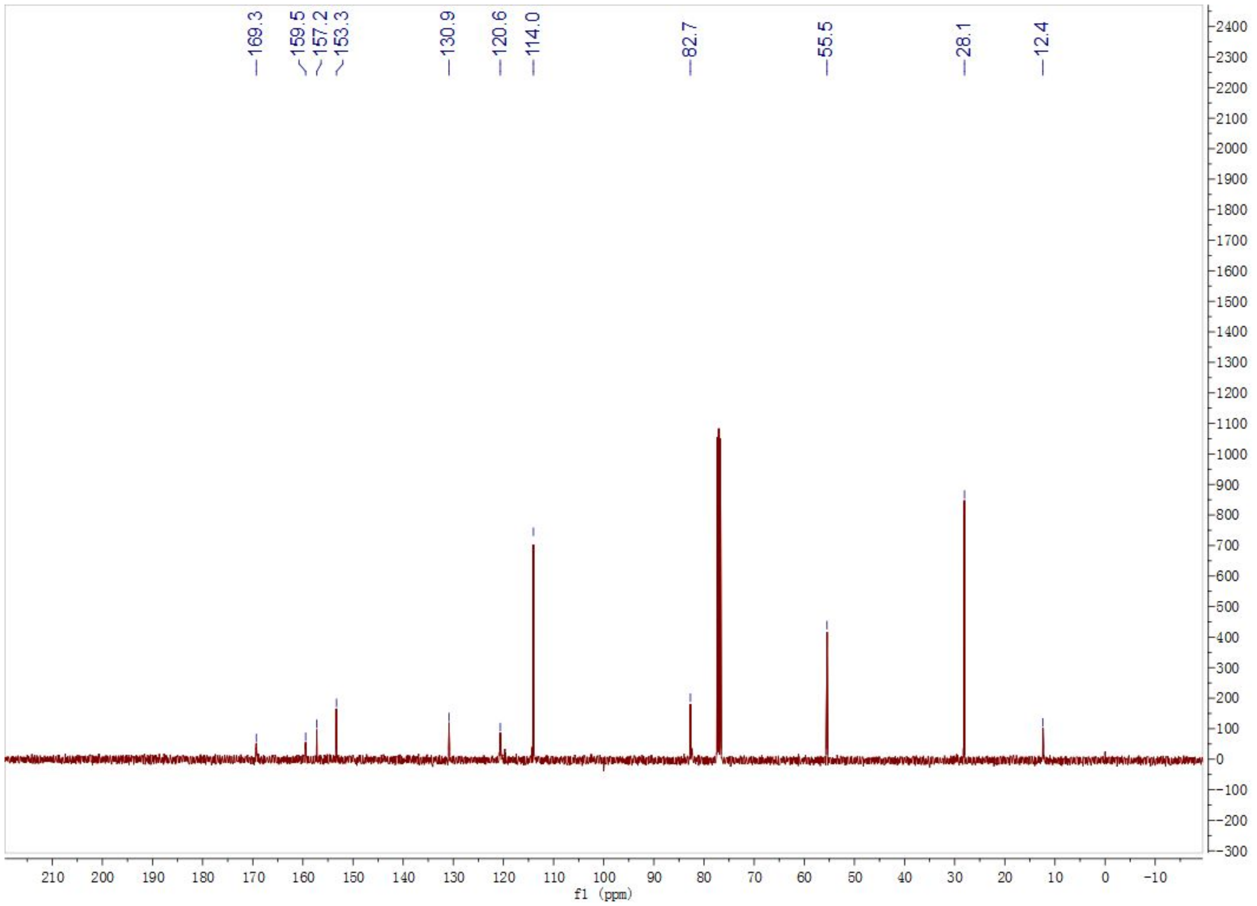
169.3
159.5
157.2
153.3
130.9
120.6
114.0
82.7
55.5
28.1
12.4
f1 (ppm)

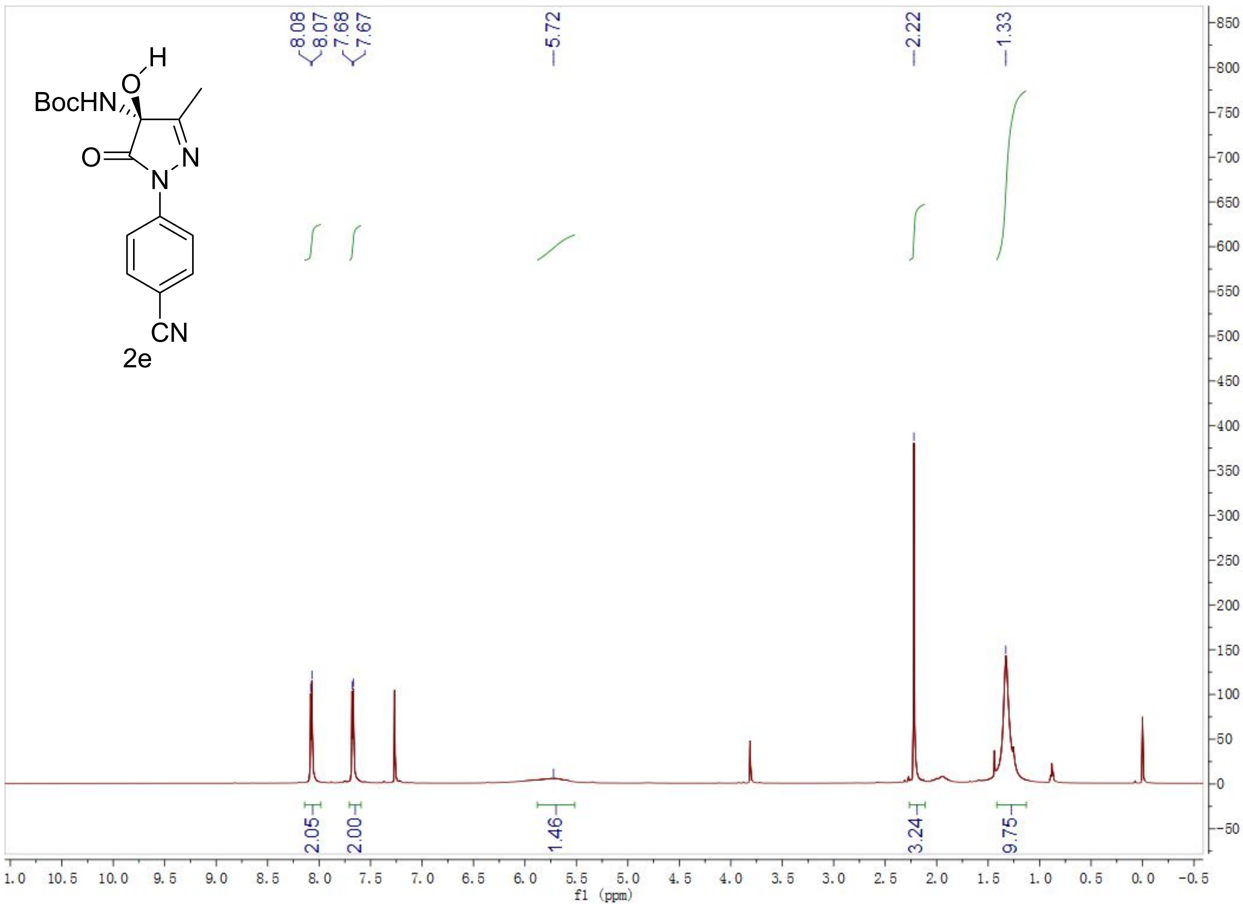
BocHN
O
H
O
N
N
CN
2e
8.08
8.07
7.68
7.67
5.72
2.22
1.33
2.05
2.00
1.46
3.24
9.75
f1 (ppm)

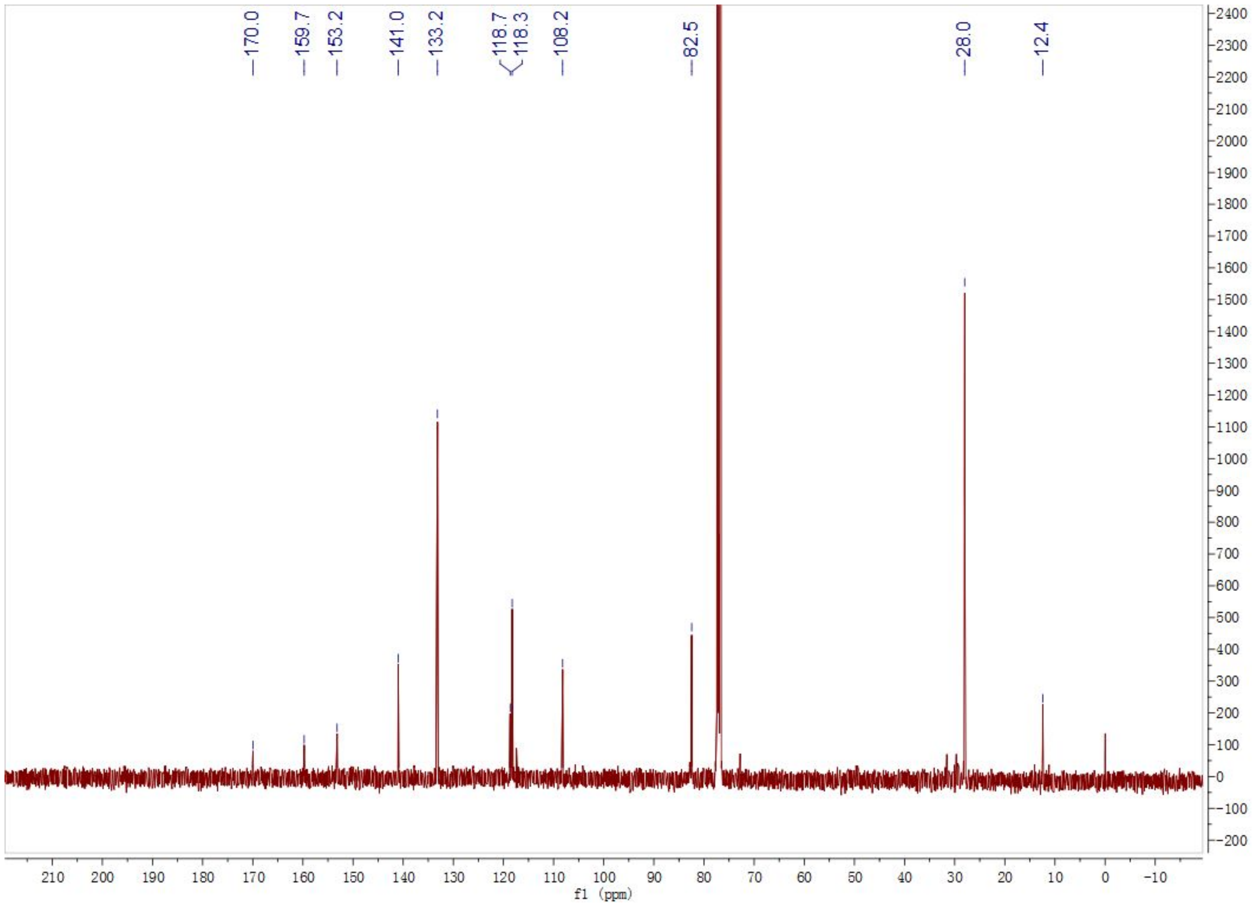
170.0
159.7
153.2
141.0
133.2
118.7
118.3
108.2
82.5
28.0
12.4
f1 (ppm)

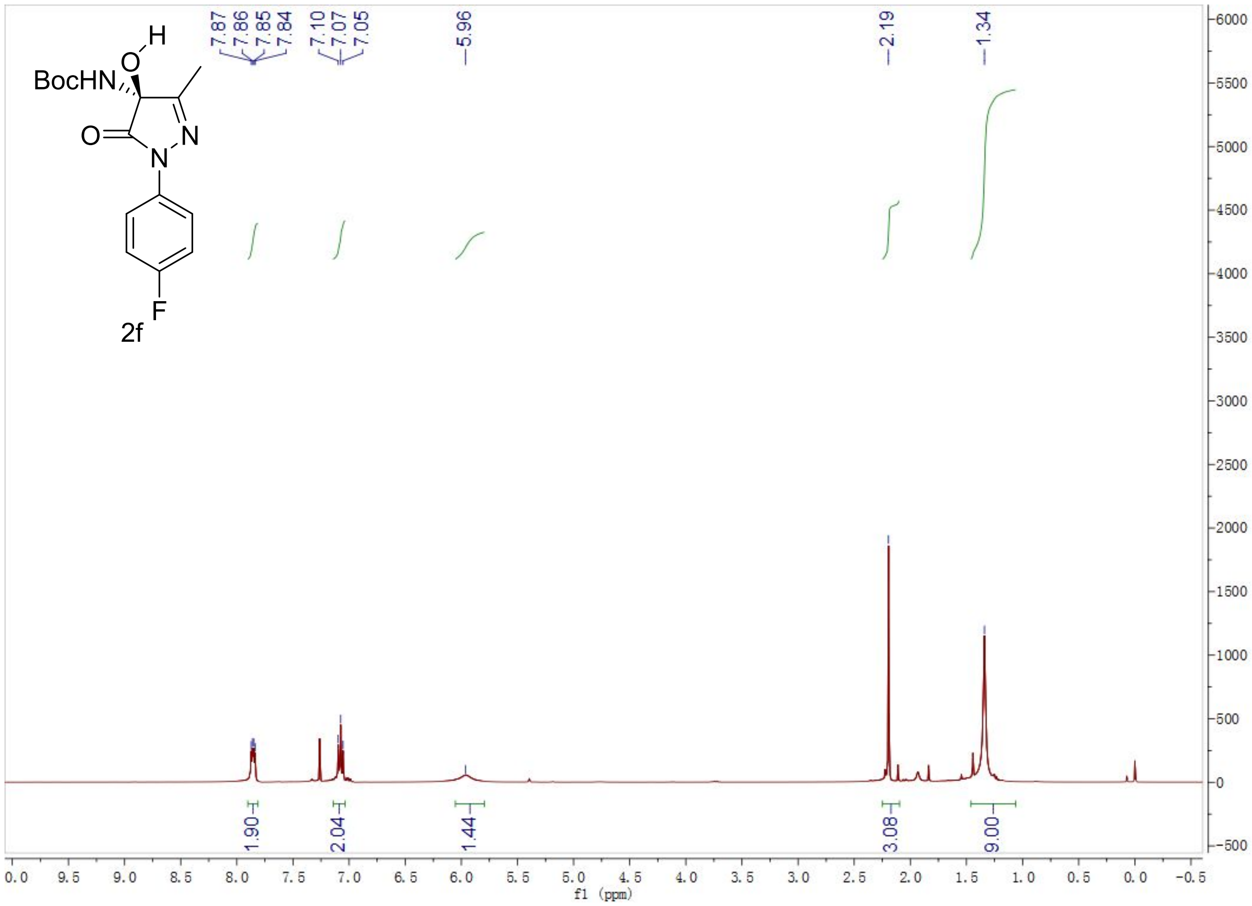
BocHN
O
H
O
N
N
F
2f
7.87
7.86
7.85
7.84
7.10
7.07
7.05
5.96
2.19
1.34
1.90
2.04
1.44
3.08
9.00
f1 (ppm)

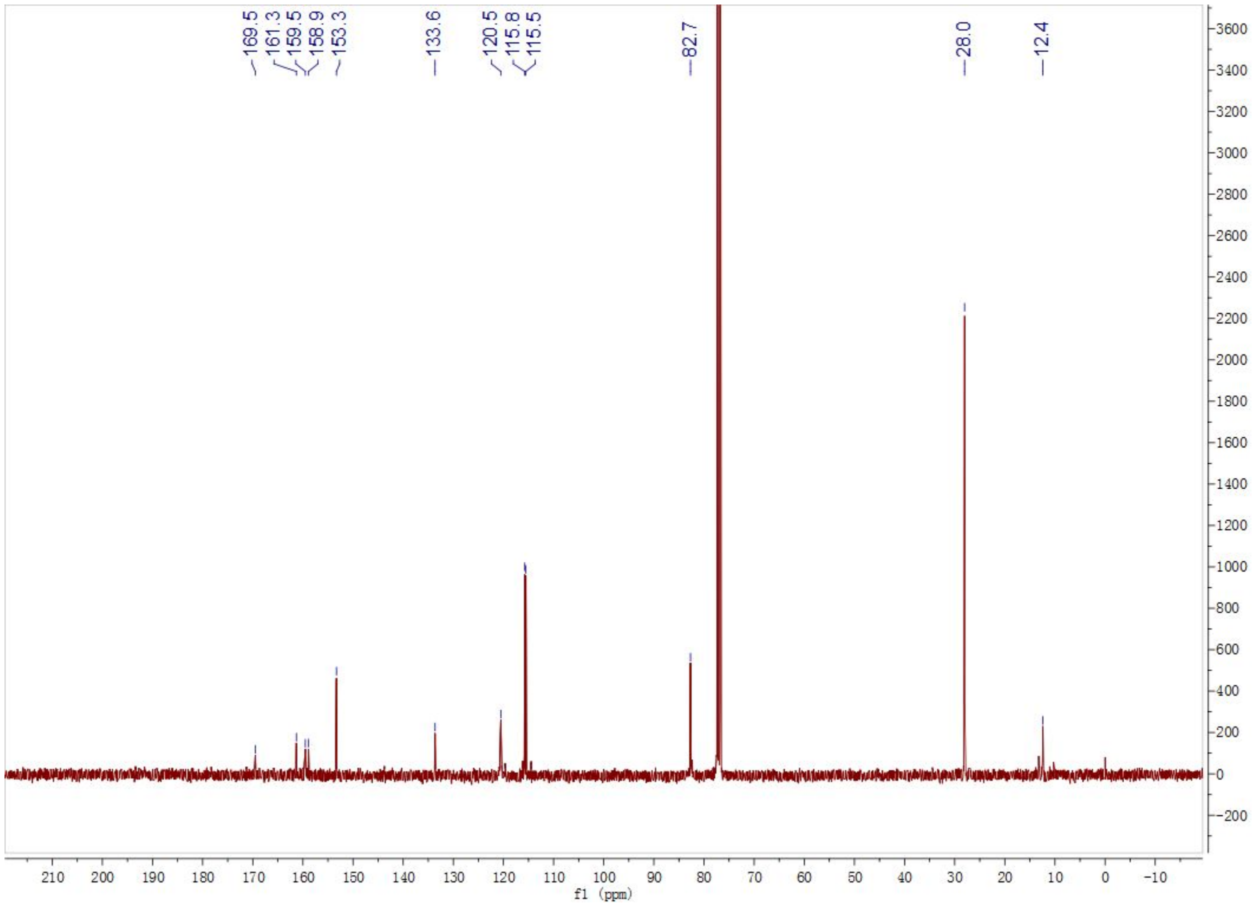
169.5
161.3
159.5
158.9
153.3
133.6
120.5
115.8
115.5
82.7
28.0
12.4
f1 (ppm)

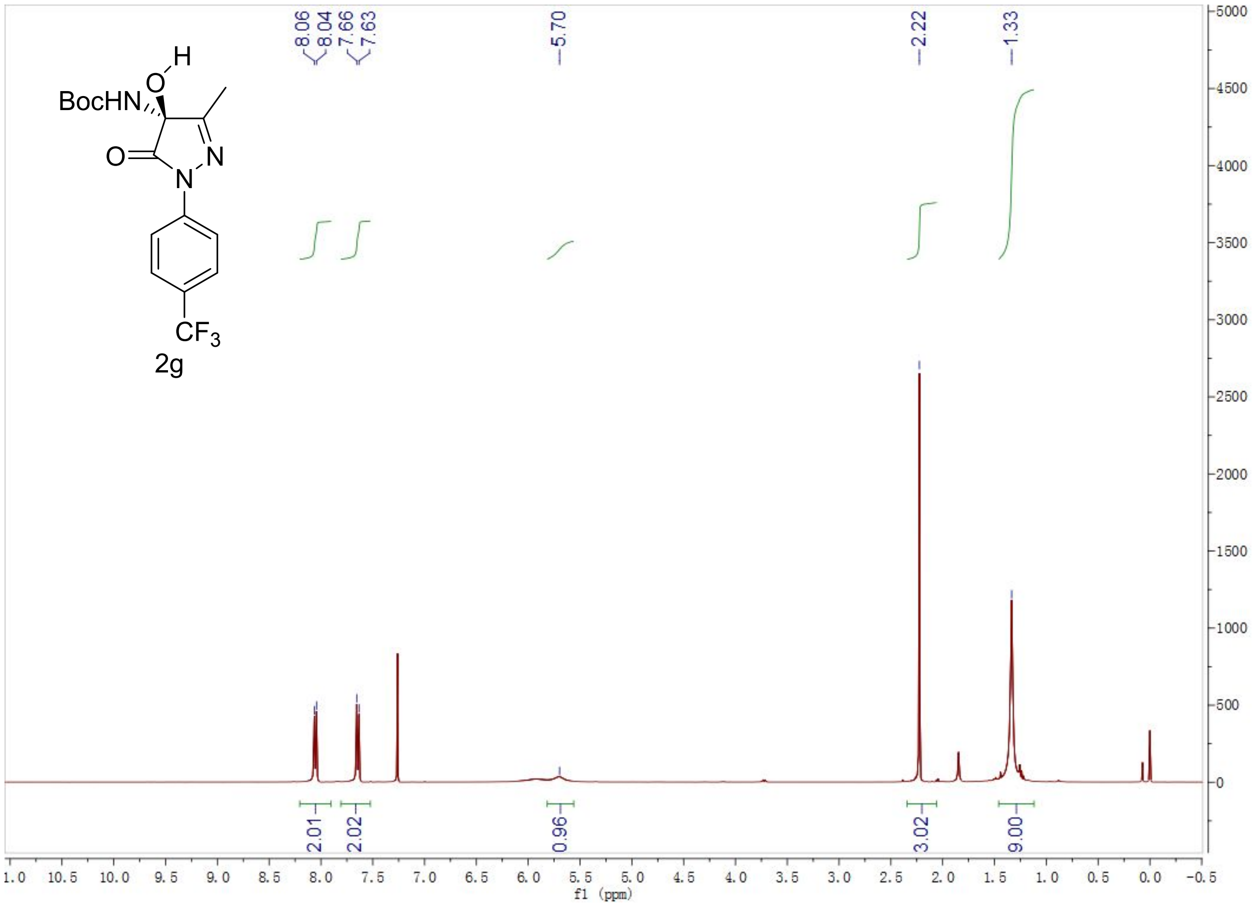

BocHN
O
H
N
N
O
CF3
2g
8.06
8.04
7.66
7.63
5.70
2.22
1.33
2.01
2.02
0.96
3.02
9.00
f1 (ppm)

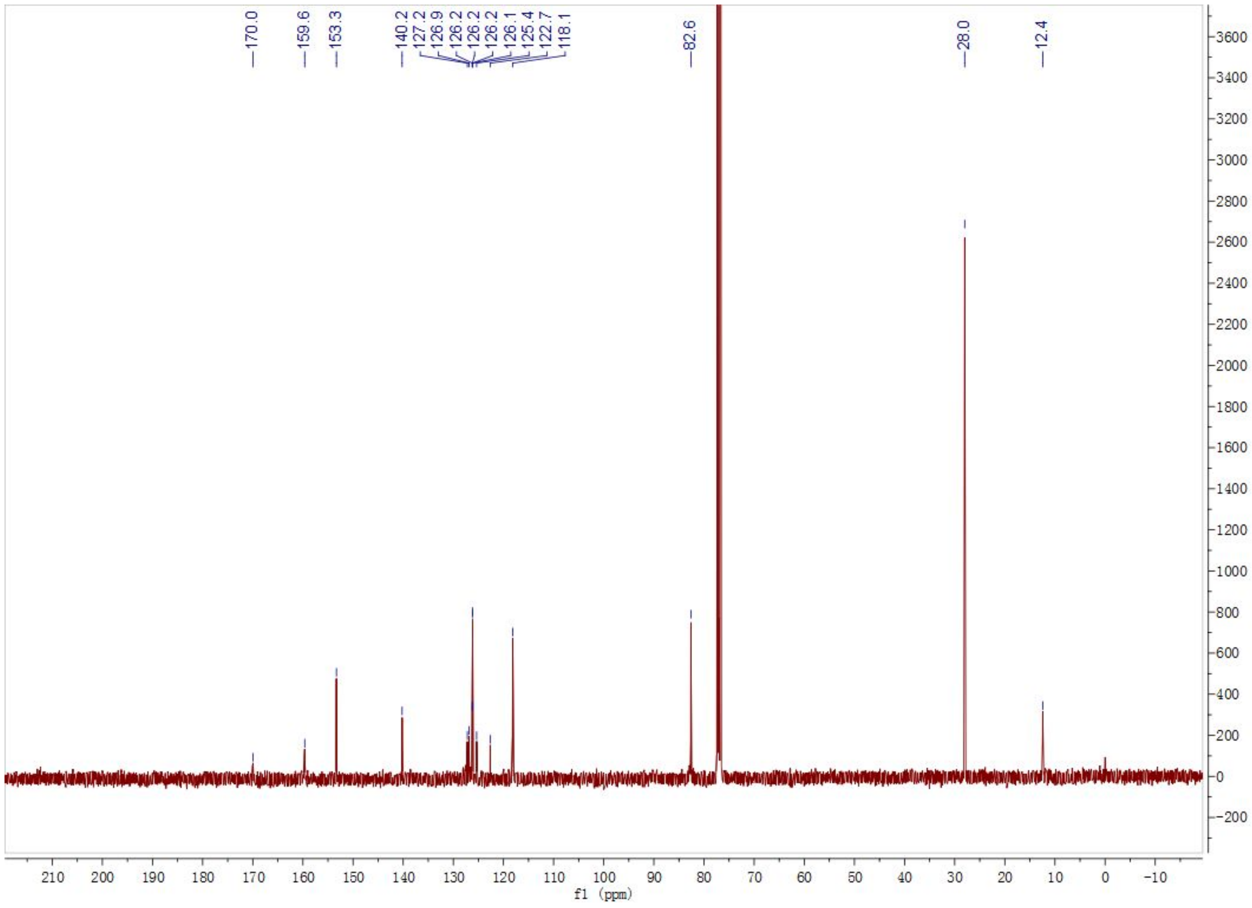

170.0
159.6
153.3
140.2
127.2
126.9
126.2
126.2
126.2
126.1
125.4
122.7
118.1
82.6
28.0
12.4
f1 (ppm)

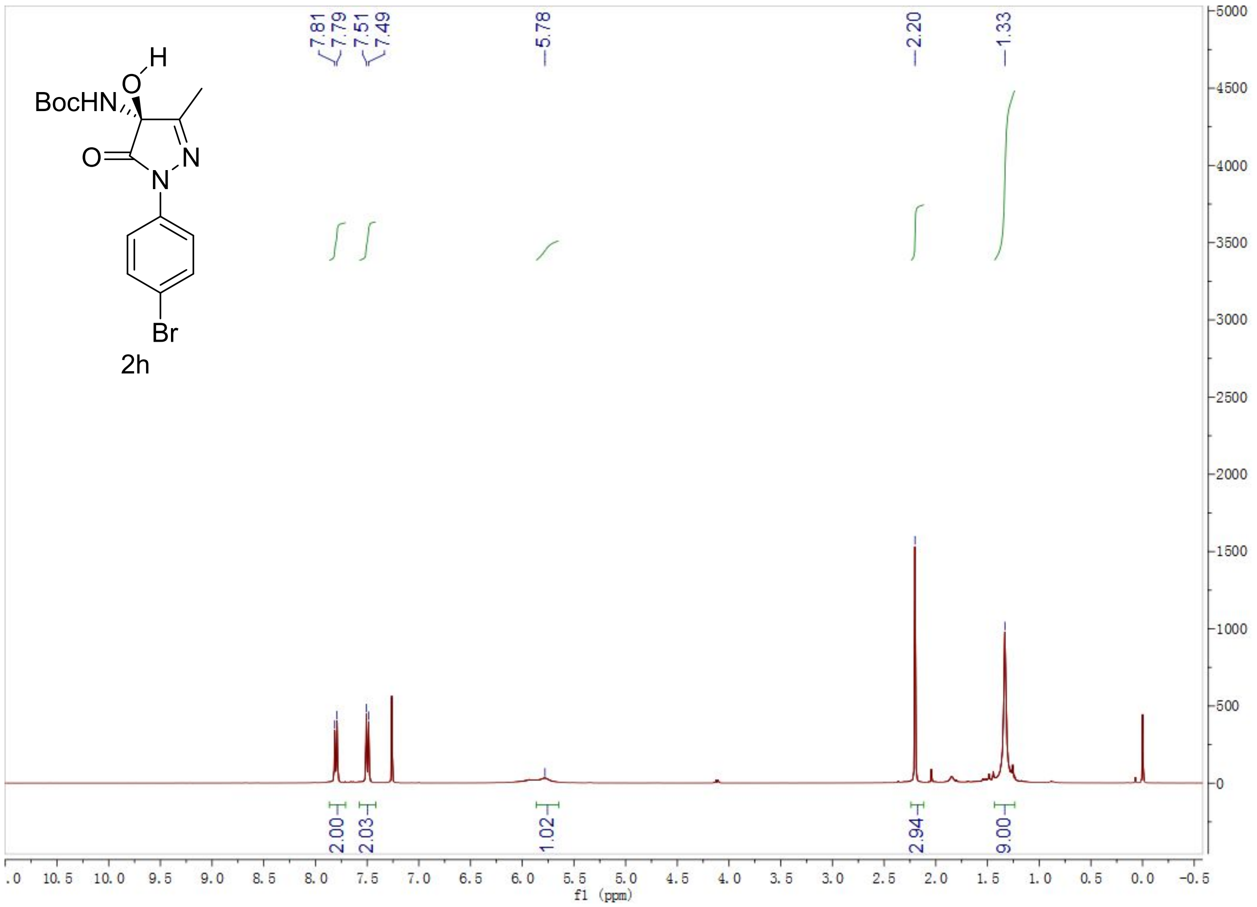
7.81
7.79
7.51
7.49
5.78
2.20
1.33
BocHN
O
H
O
N
N
Br
2h
2.00
2.03
1.02
2.94
9.00
f1 (ppm)

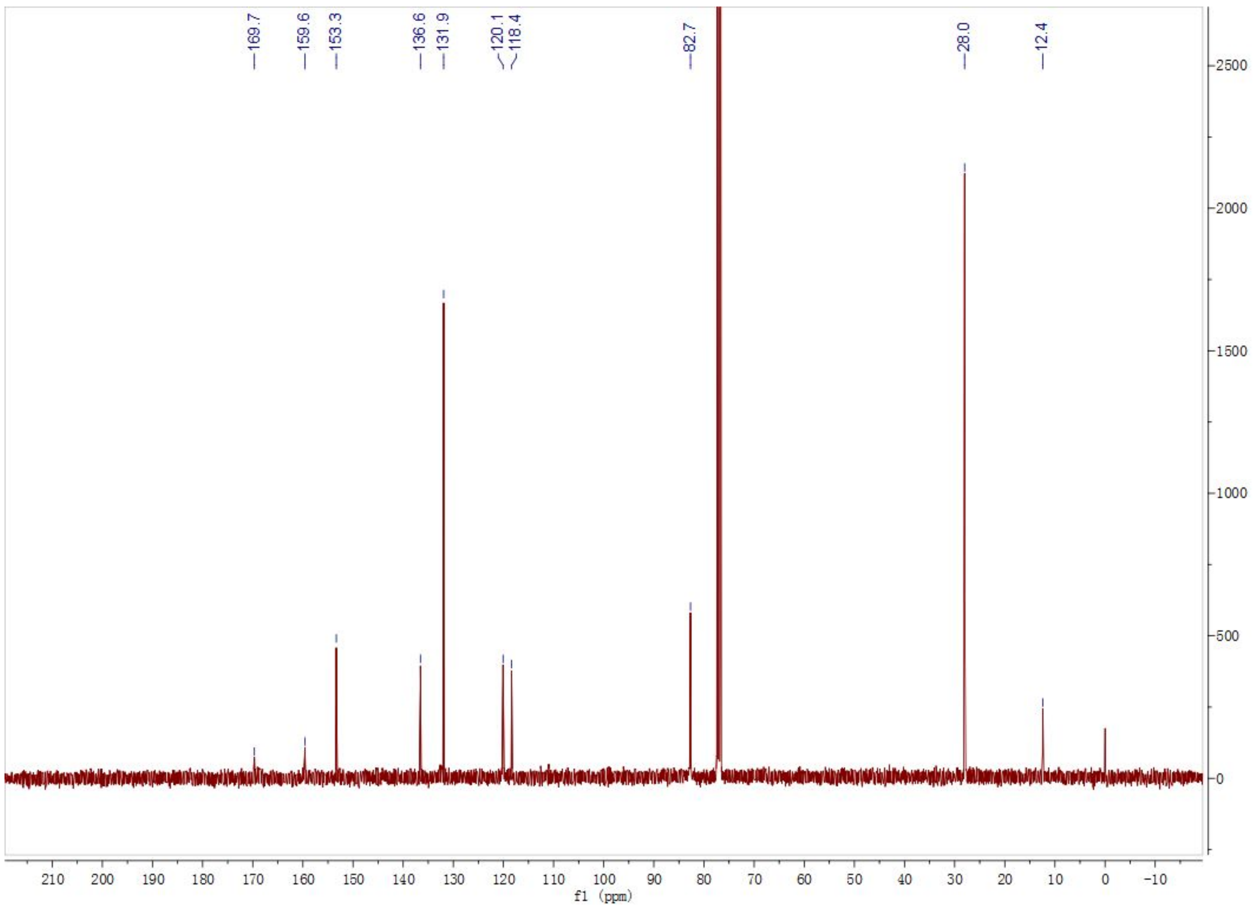
169.7
159.6
153.3
136.6
131.9
120.1
118.4
82.7
28.0
12.4
f1 (ppm)

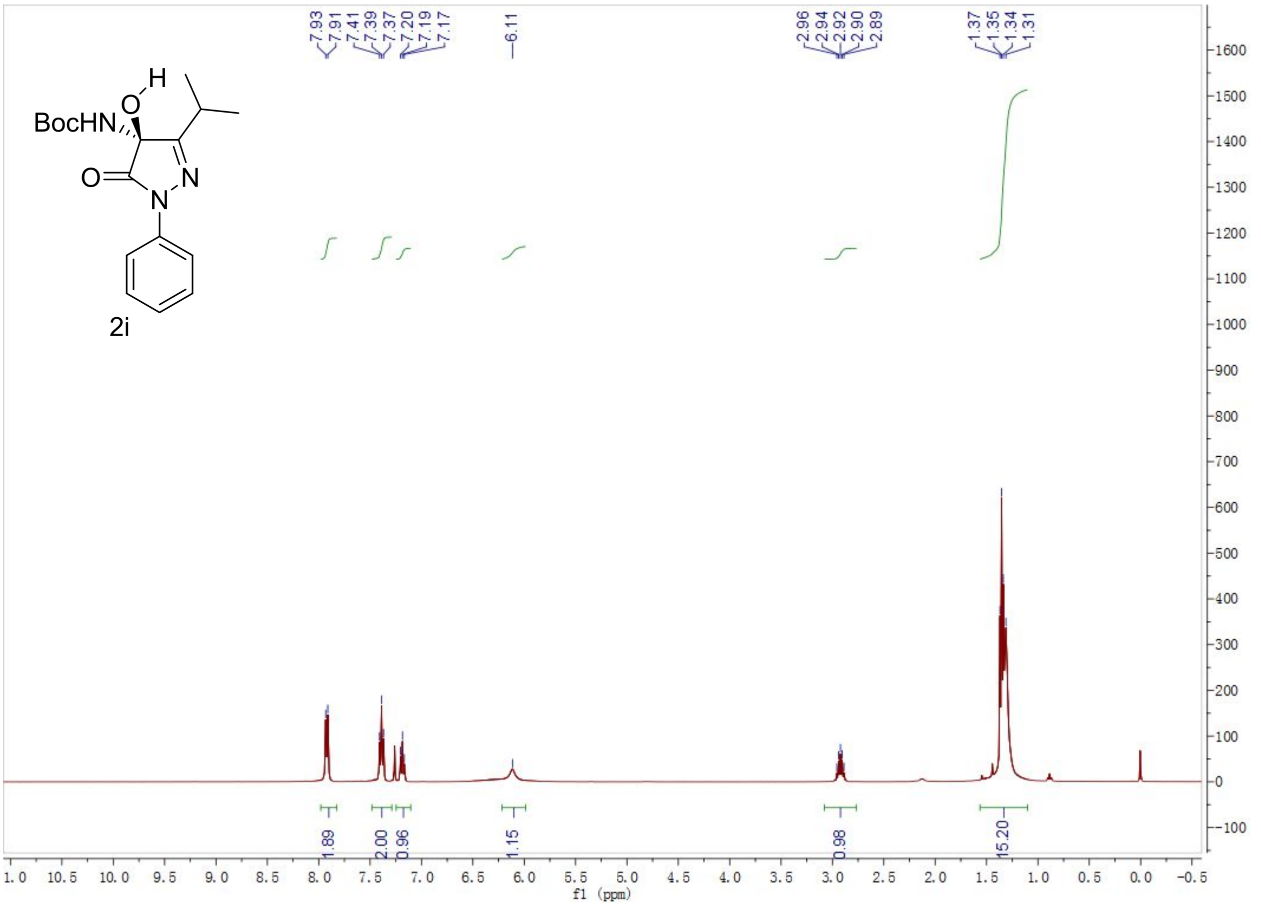
BocHN
O
H
O
N
N
2i
7.93
7.91
7.41
7.39
7.37
7.20
7.19
7.17
6.11
2.96
2.94
2.92
2.90
2.89
1.37
1.35
1.34
1.31
1.89
2.00
0.96
1.15
0.98
15.20
f1 (ppm)

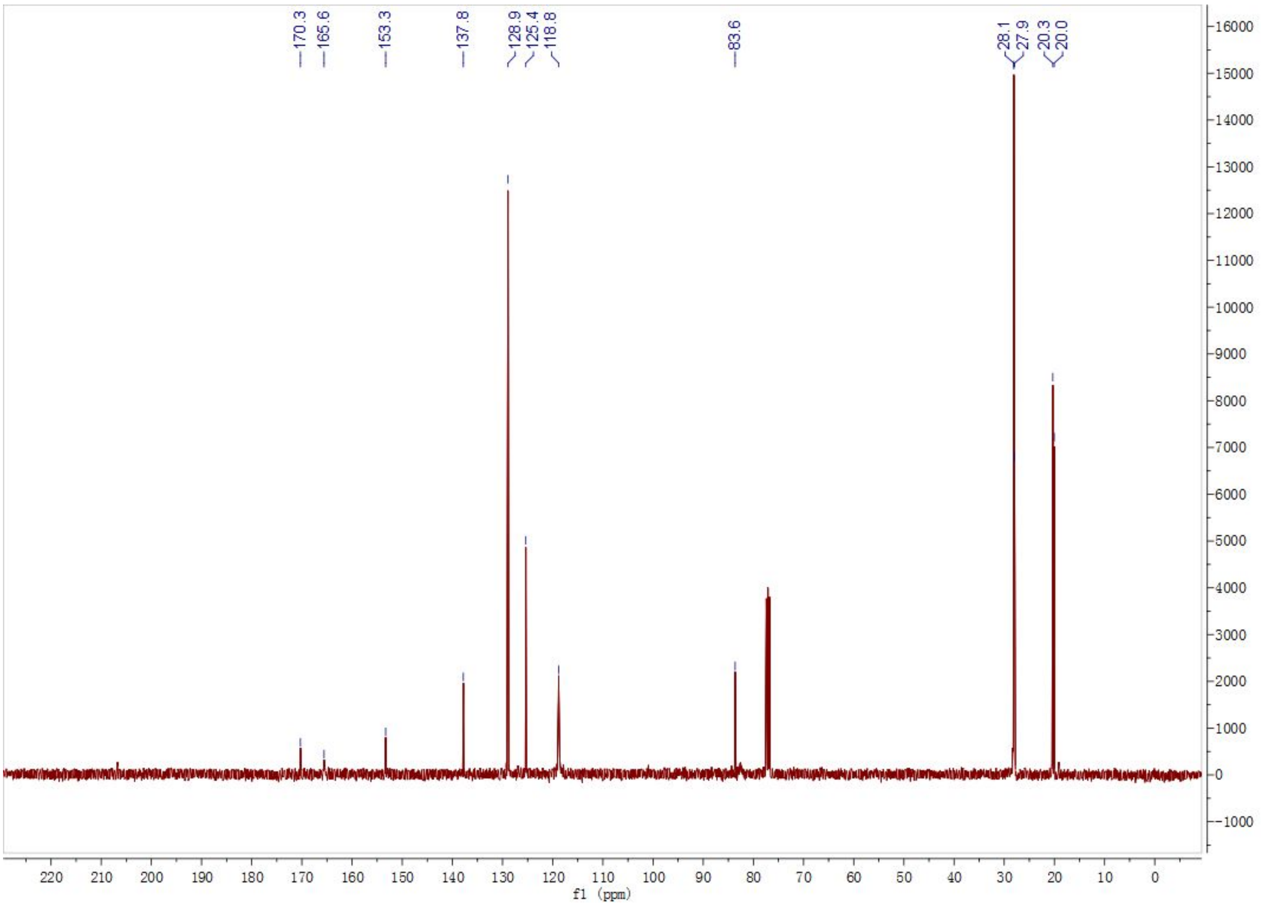
170.3
165.6
153.3
137.8
128.9
125.4
118.8
83.6
28.1
27.9
20.3
20.0
f1 (ppm)

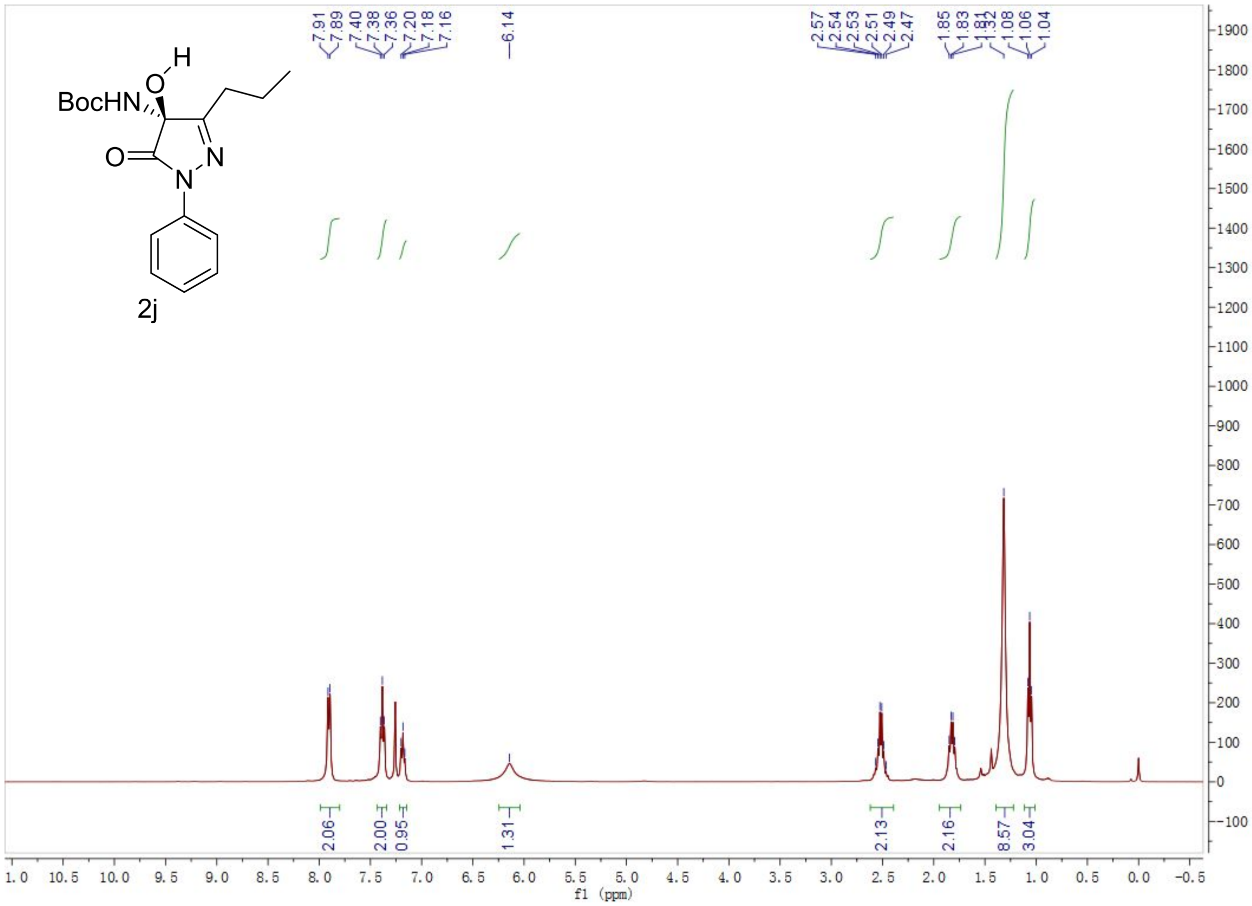
7.91
7.89
7.40
7.38
7.36
7.20
7.18
7.16
6.14
2.57
2.54
2.53
2.51
2.49
2.47
1.85
1.83
1.81
1.32
1.08
1.06
1.04
BocHN
O
H
O
N
N
2j
2.06
2.00
0.95
1.31
2.13
2.16
8.57
3.04
f1 (ppm)

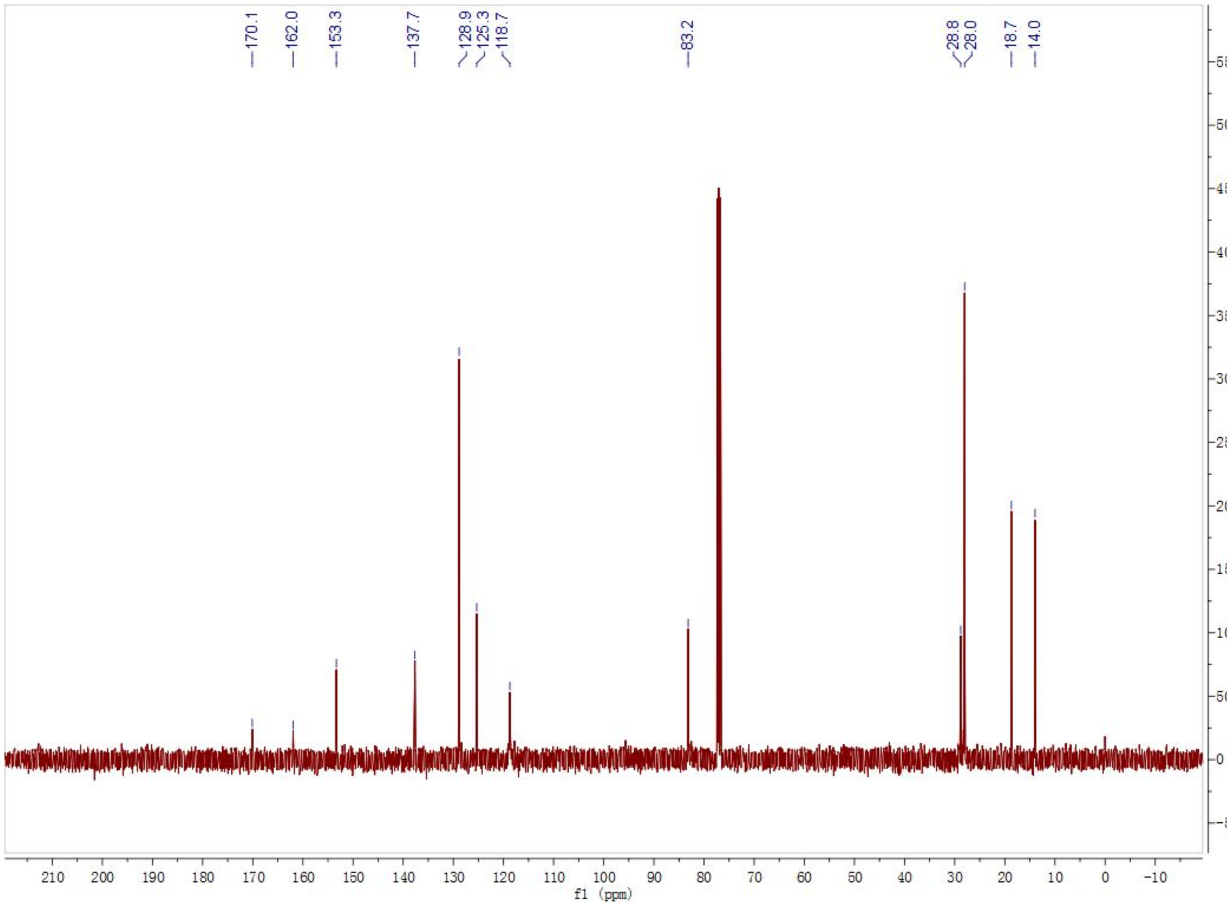
170.1
162.0
153.3
137.7
128.9
125.3
118.7
83.2
28.8
28.0
18.7
14.0
f1 (ppm)

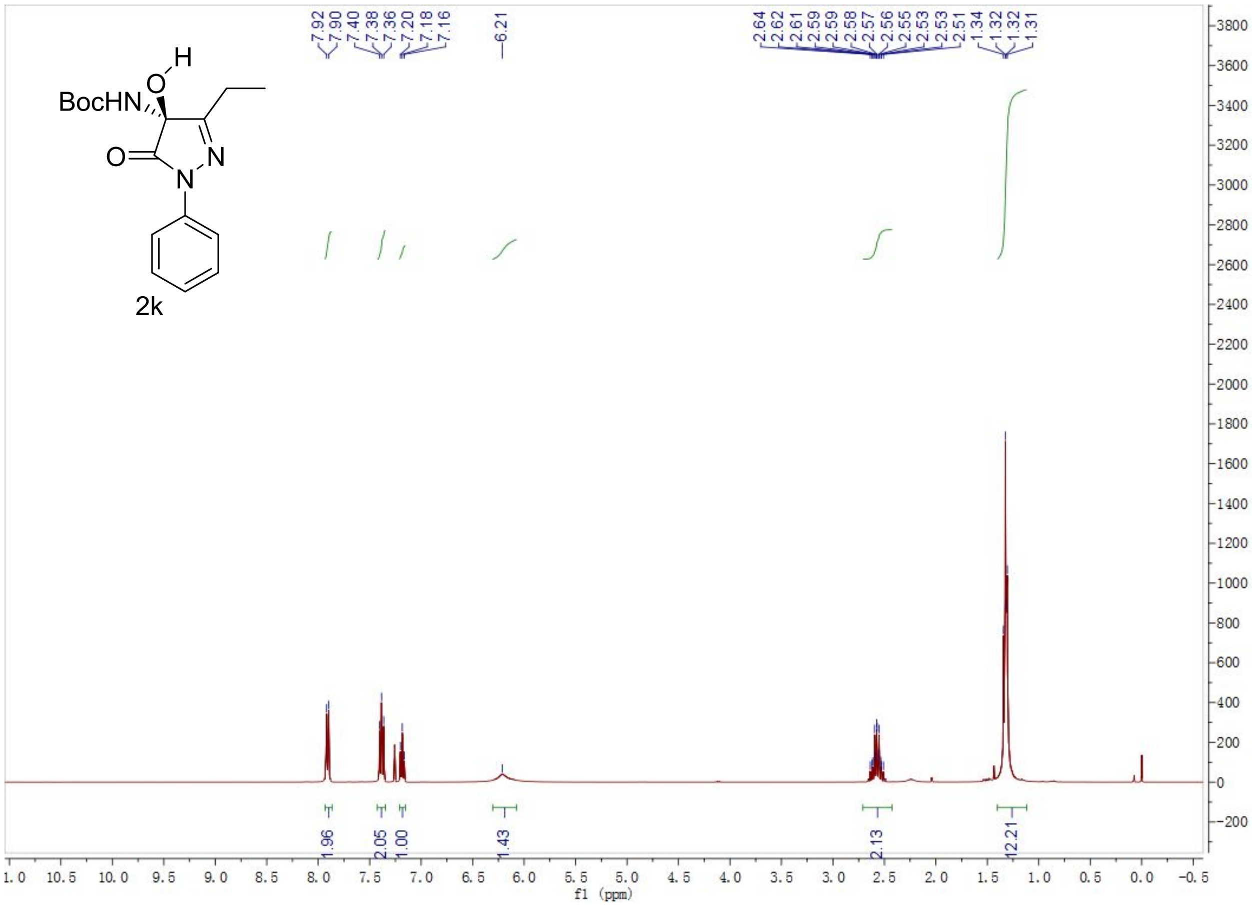
BocHN
O
H
O
N
N
2k
7.92
7.90
7.40
7.38
7.36
7.20
7.18
7.16
6.21
2.64
2.62
2.61
2.59
2.59
2.58
2.57
2.56
2.55
2.53
2.53
2.51
1.34
1.32
1.32
1.31
1.96
2.05
1.00
1.43
2.13
12.21
f1 (ppm)

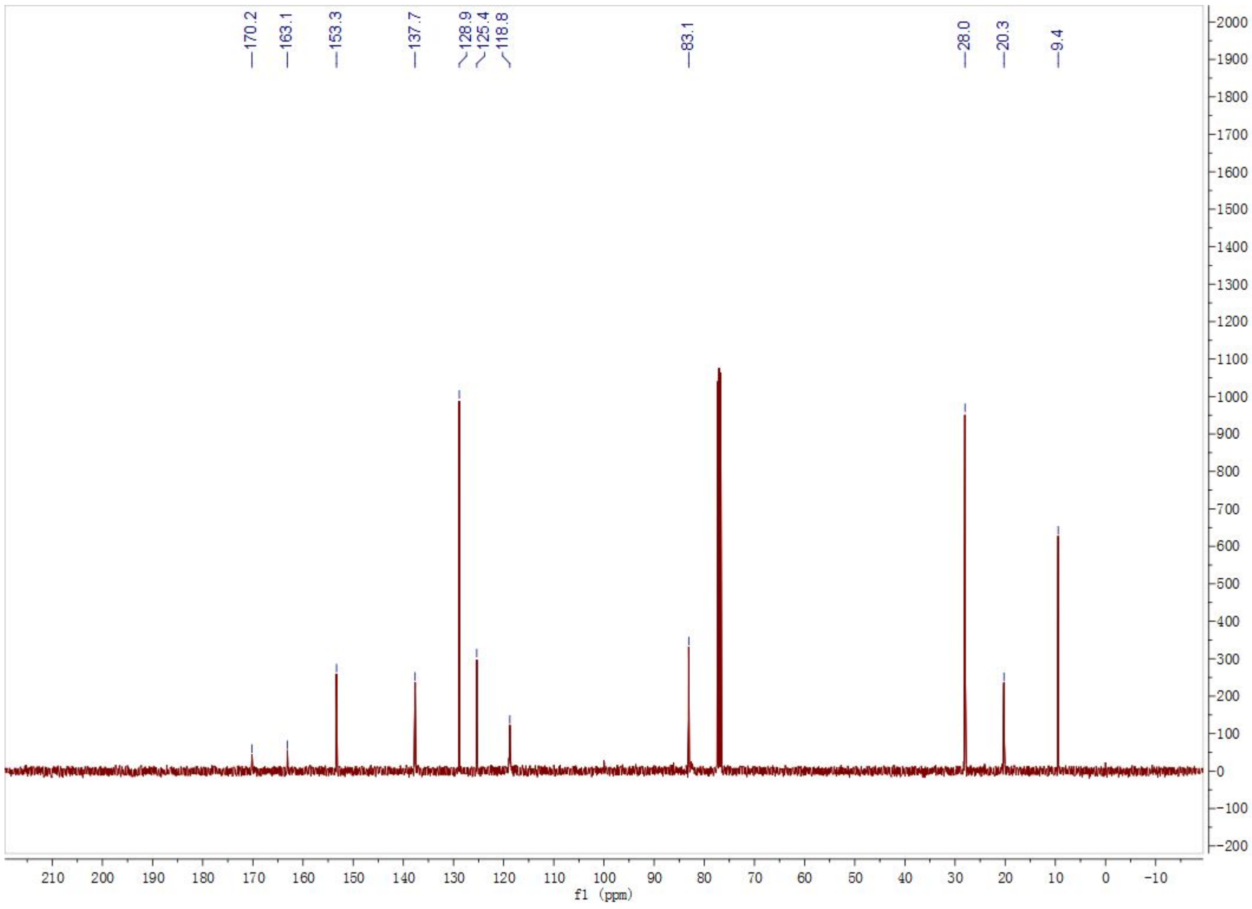
170.2
163.1
153.3
137.7
128.9
125.4
118.8
83.1
28.0
20.3
9.4
f1 (ppm)

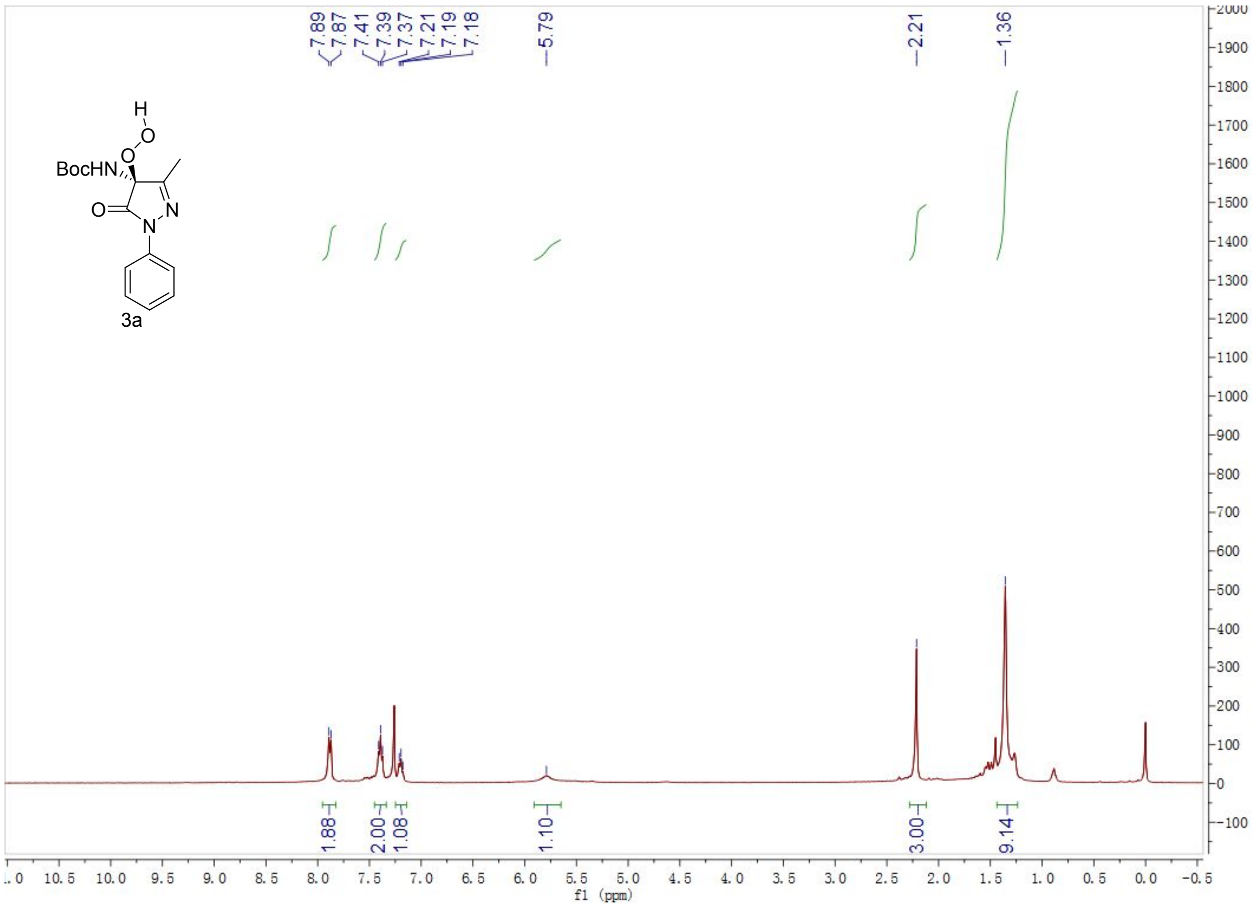
7.89
7.87
7.41
7.39
7.37
7.21
7.19
7.18
5.79
2.21
1.36
BocHN
O
H
O
N
N
3a
1.88
2.00
1.08
1.10
3.00
9.14
f1 (ppm)

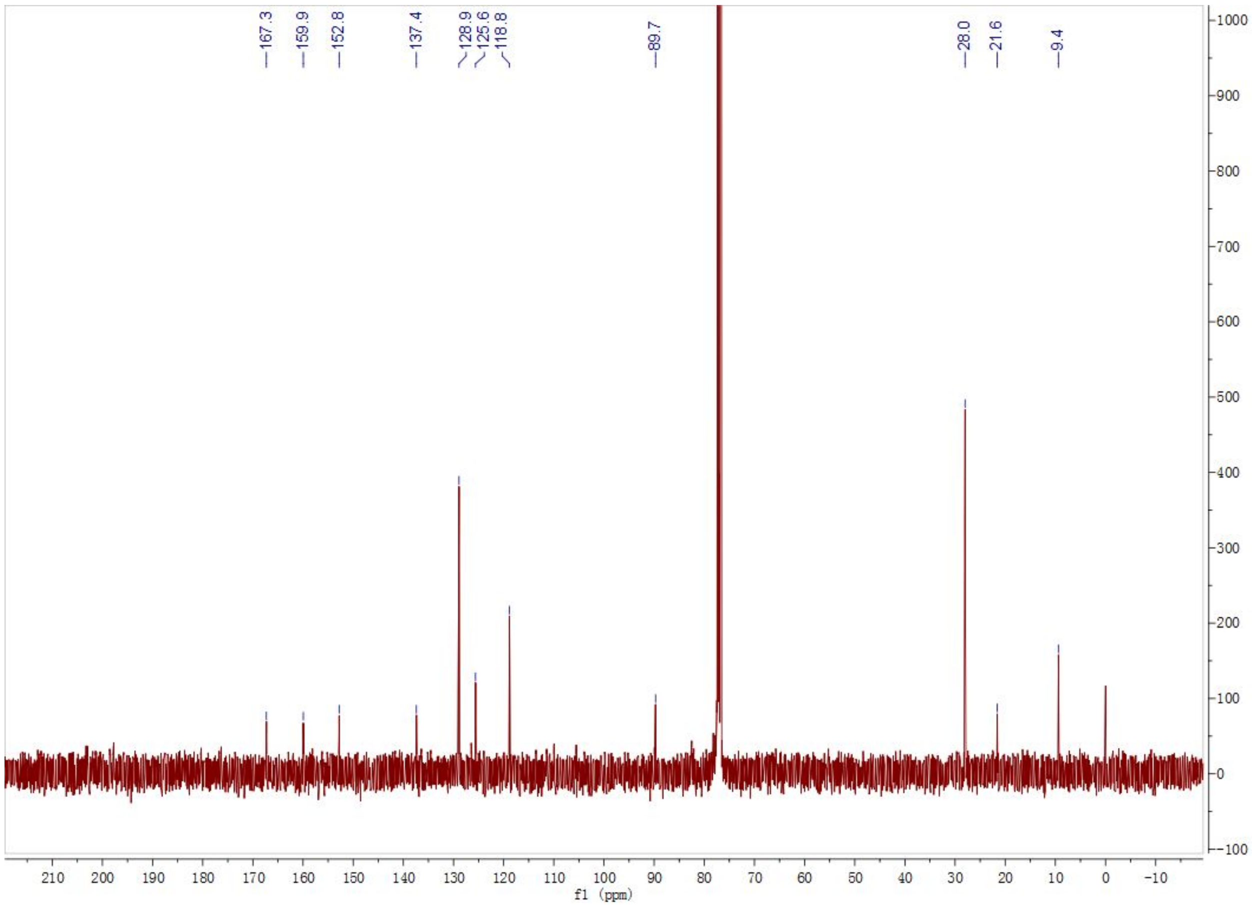
167.3
159.9
152.8
137.4
128.9
125.6
118.8
89.7
28.0
21.6
9.4
f1 (ppm)

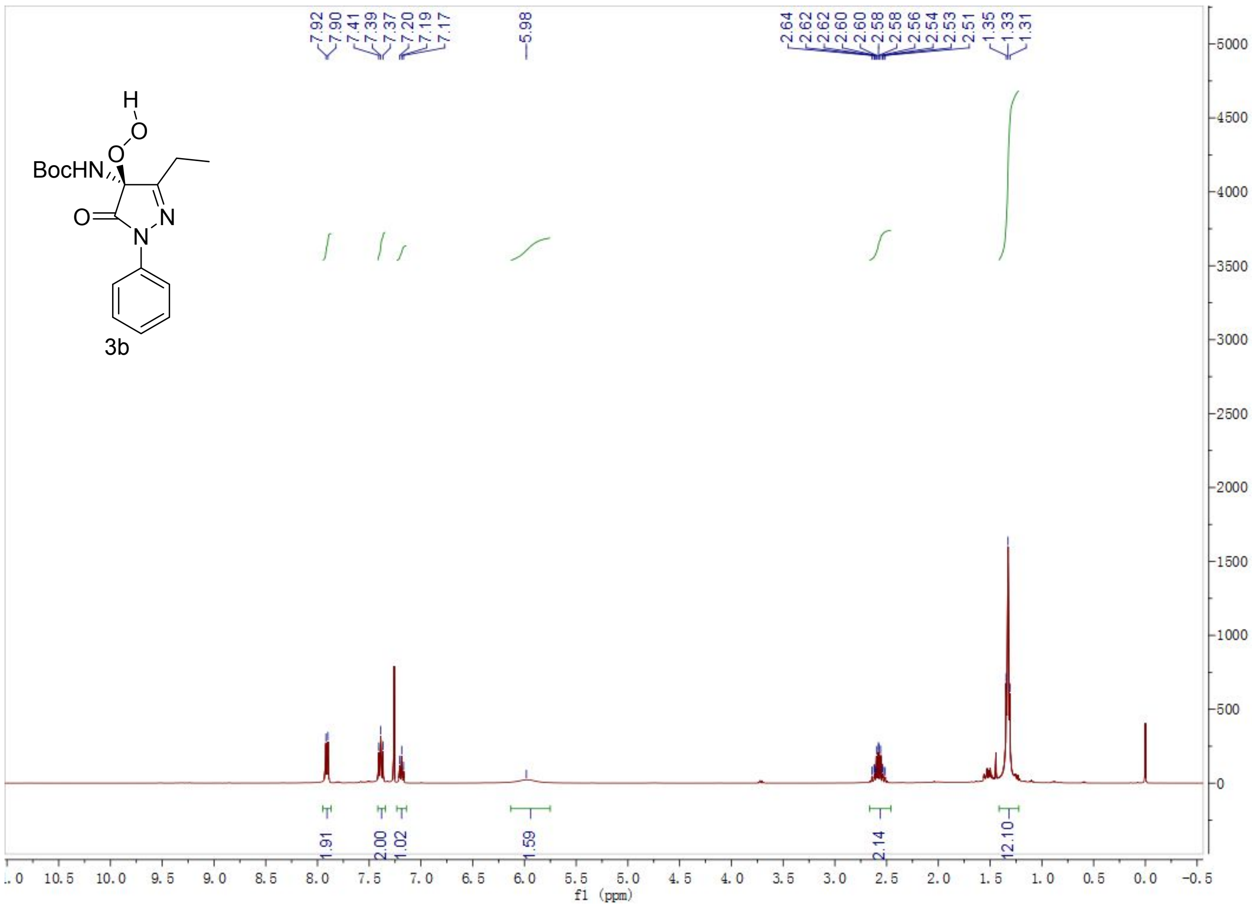

7.92
7.90
7.41
7.39
7.37
7.20
7.19
7.17
5.98
2.64
2.62
2.62
2.60
2.60
2.58
2.58
2.56
2.54
2.53
2.51
1.35
1.33
1.31
BocHN
H
O
O
N
N
3b
1.91
2.00
1.02
1.59
2.14
12.10
f1 (ppm)

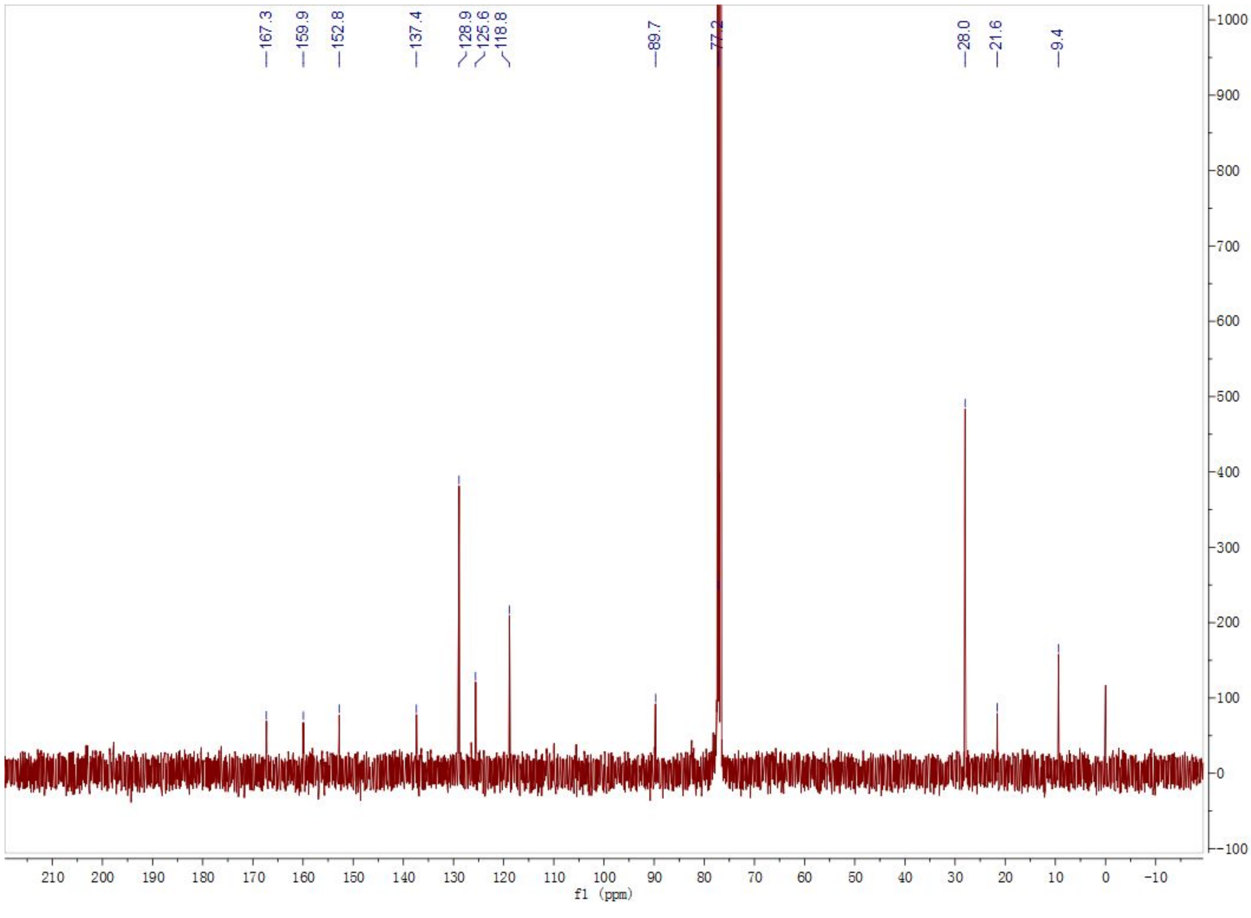

167.3
159.9
152.8
137.4
128.9
125.6
118.8
89.7
77.2
28.0
21.6
9.4
f1 (ppm)

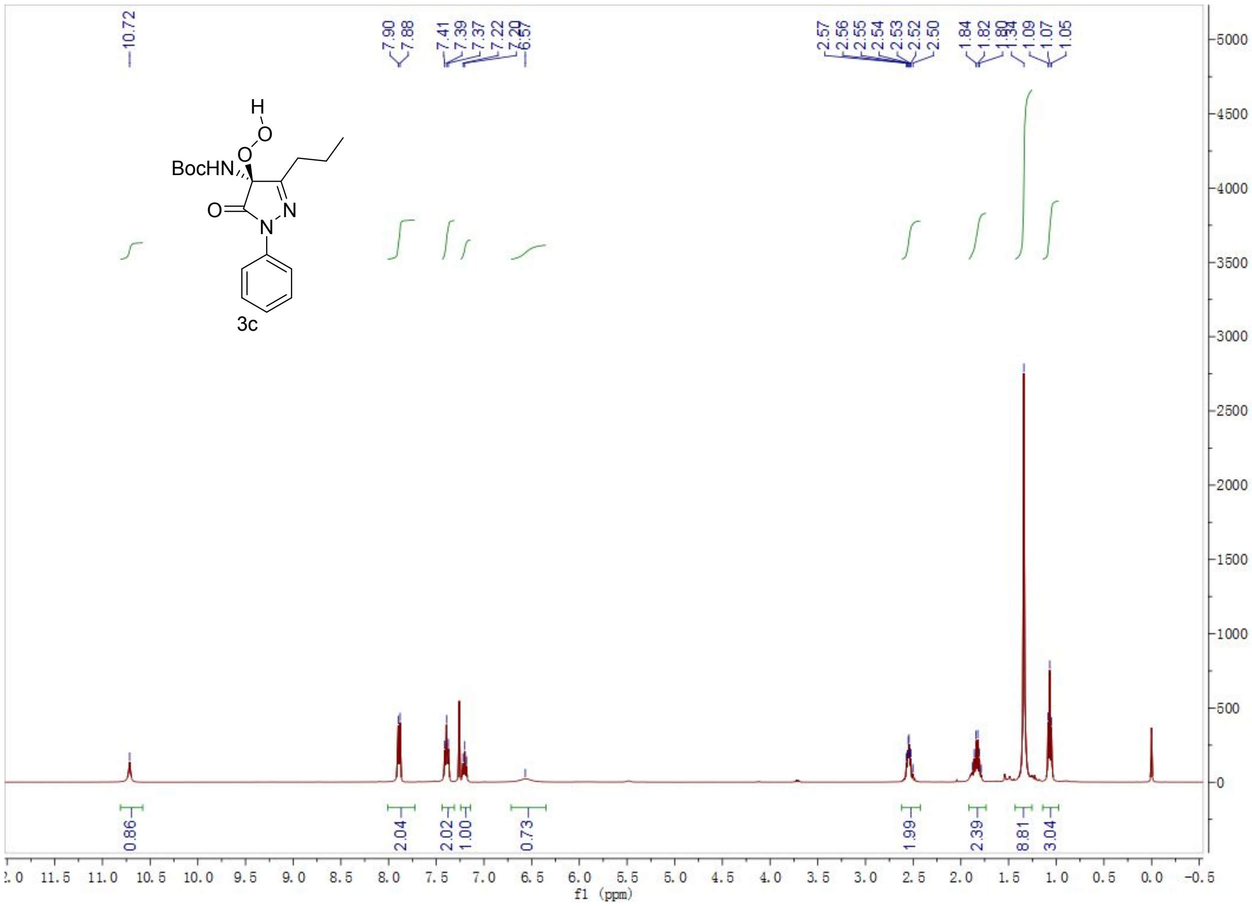

BocHN
3c
f1 (ppm)

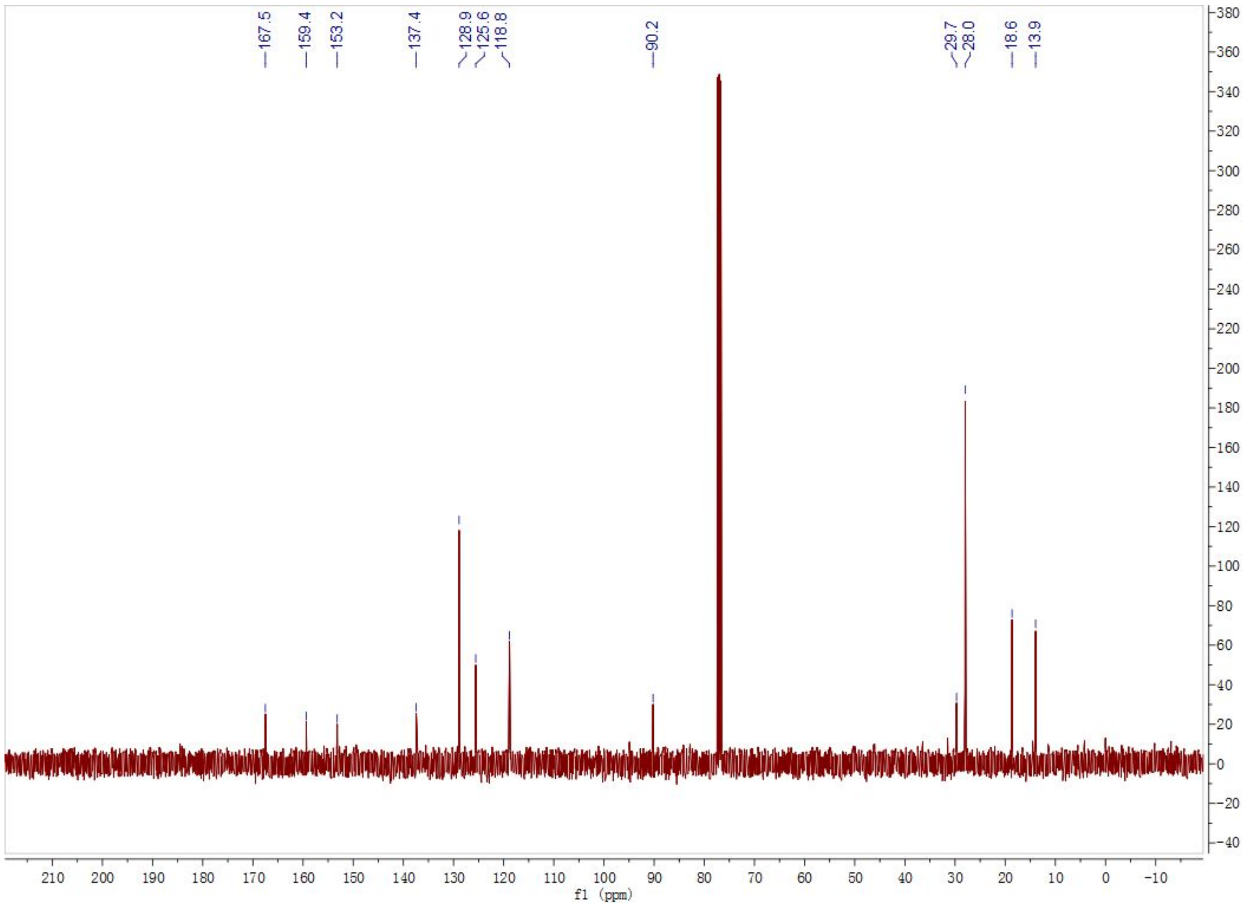

f1 (ppm)

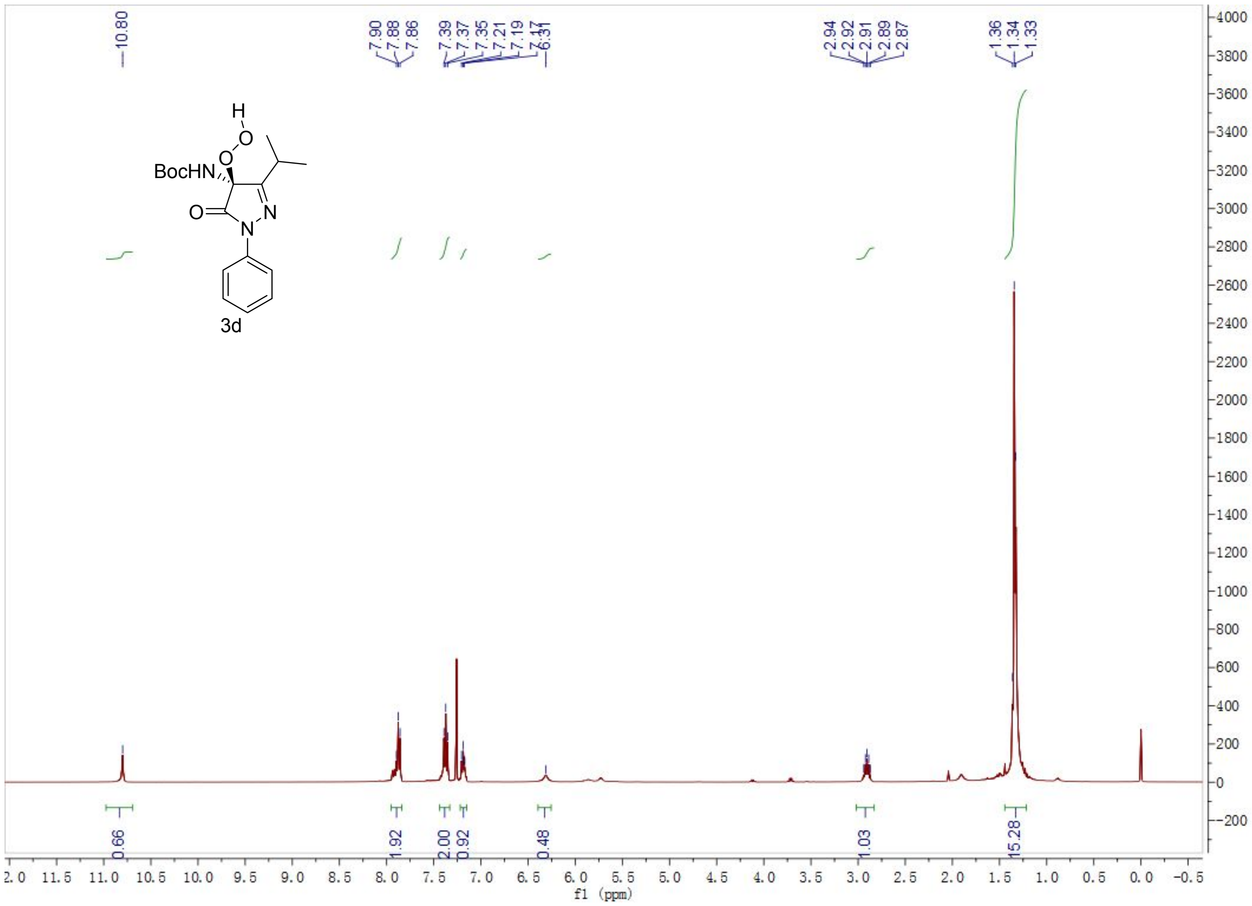
10.80
7.90
7.88
7.86
7.39
7.37
7.35
7.21
7.19
7.17
6.31
2.94
2.92
2.91
2.89
2.87
1.36
1.34
1.33
BocHN
H
O
O
N
N
3d
0.66
1.92
2.00
0.92
0.48
1.03
15.28
f1 (ppm)

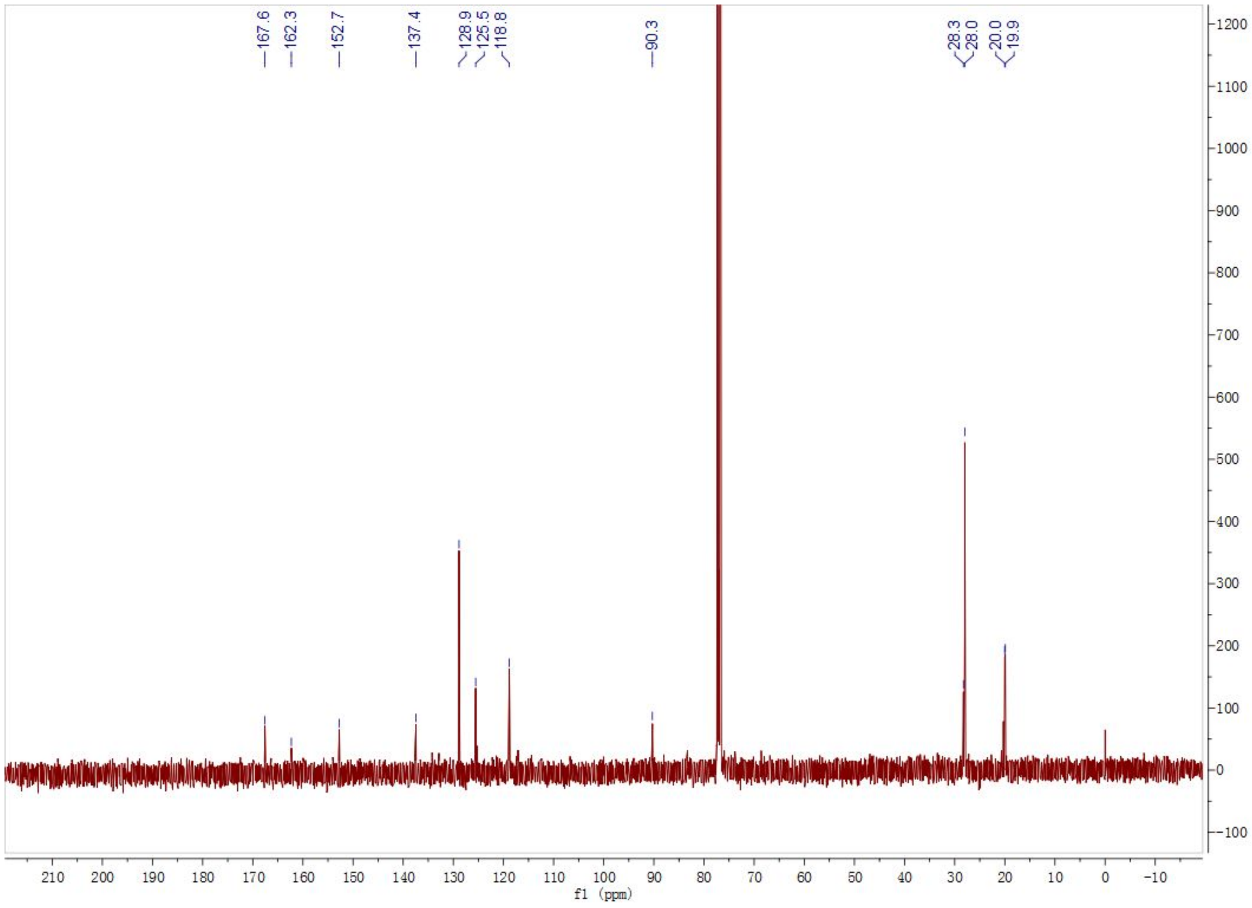
167.6
162.3
152.7
137.4
128.9
125.5
118.8
90.3
28.3
28.0
20.0
19.9
f1 (ppm)

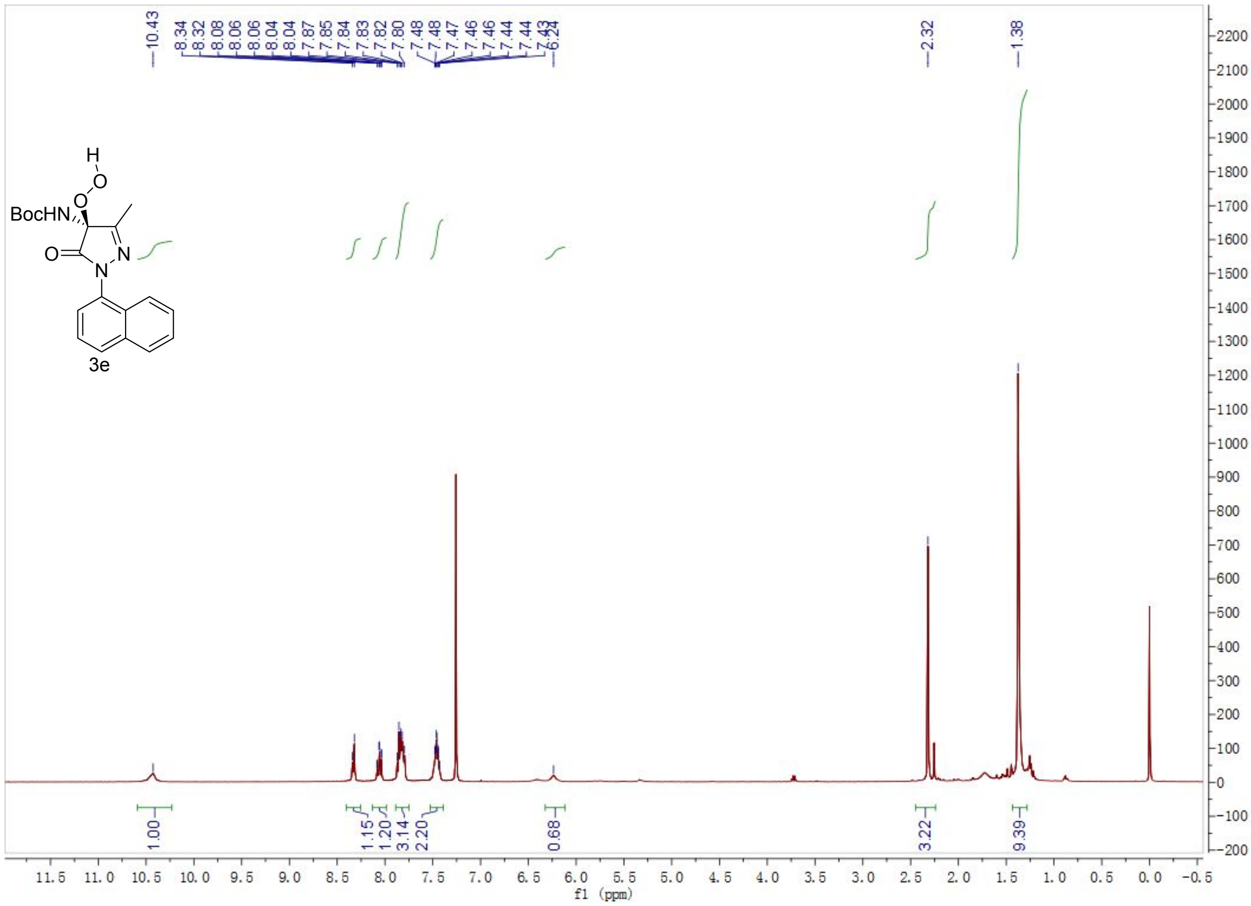
10.43
8.34
8.32
8.08
8.06
8.06
8.04
8.04
7.87
7.85
7.84
7.83
7.82
7.80
7.48
7.48
7.47
7.46
7.46
7.44
7.44
7.43
6.24
2.32
1.38
BocHN
3e
1.00
1.15
1.20
3.14
2.20
0.68
3.22
9.39
f1 (ppm)

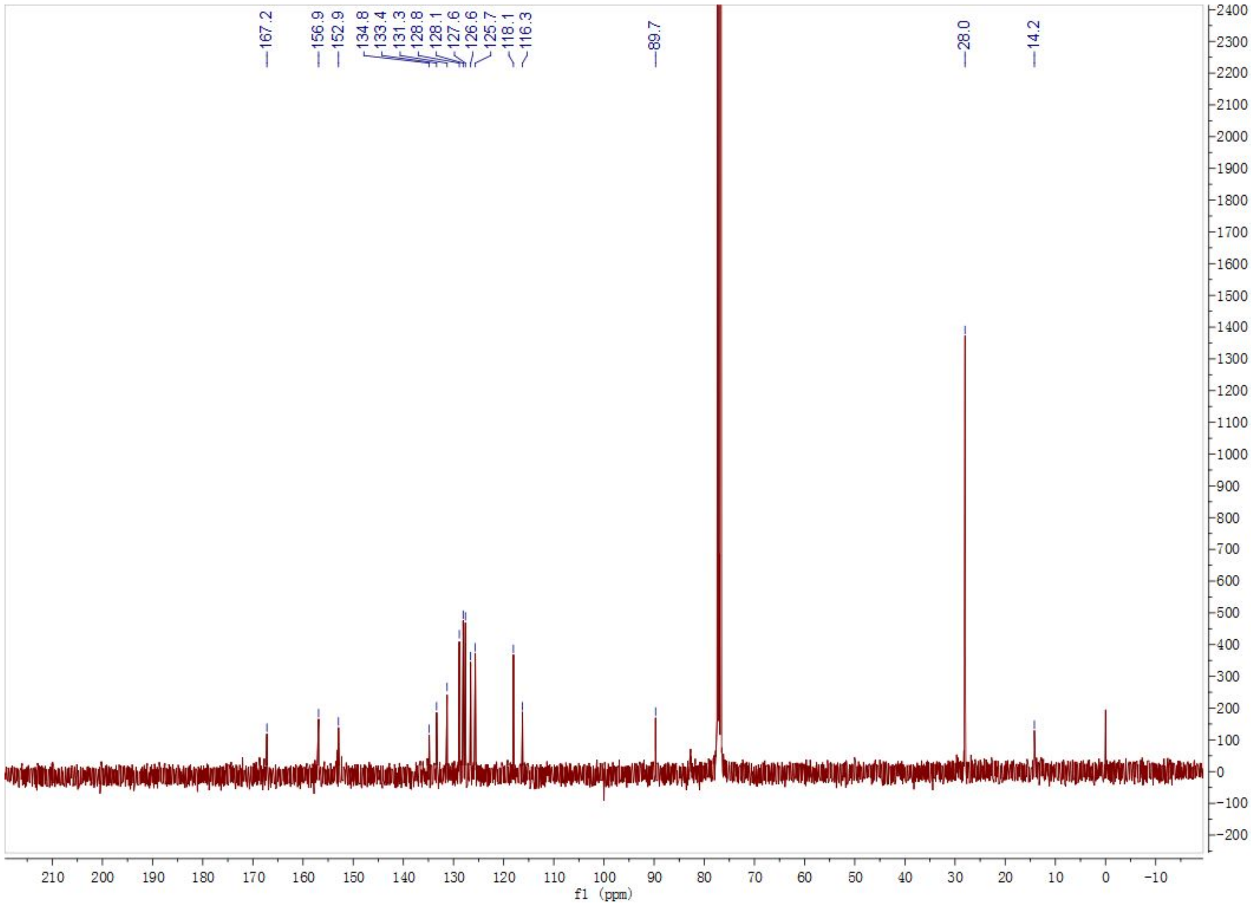
167.2
156.9
152.9
134.8
133.4
131.3
128.8
128.1
127.6
126.6
125.7
118.1
116.3
89.7
28.0
14.2
f1 (ppm)

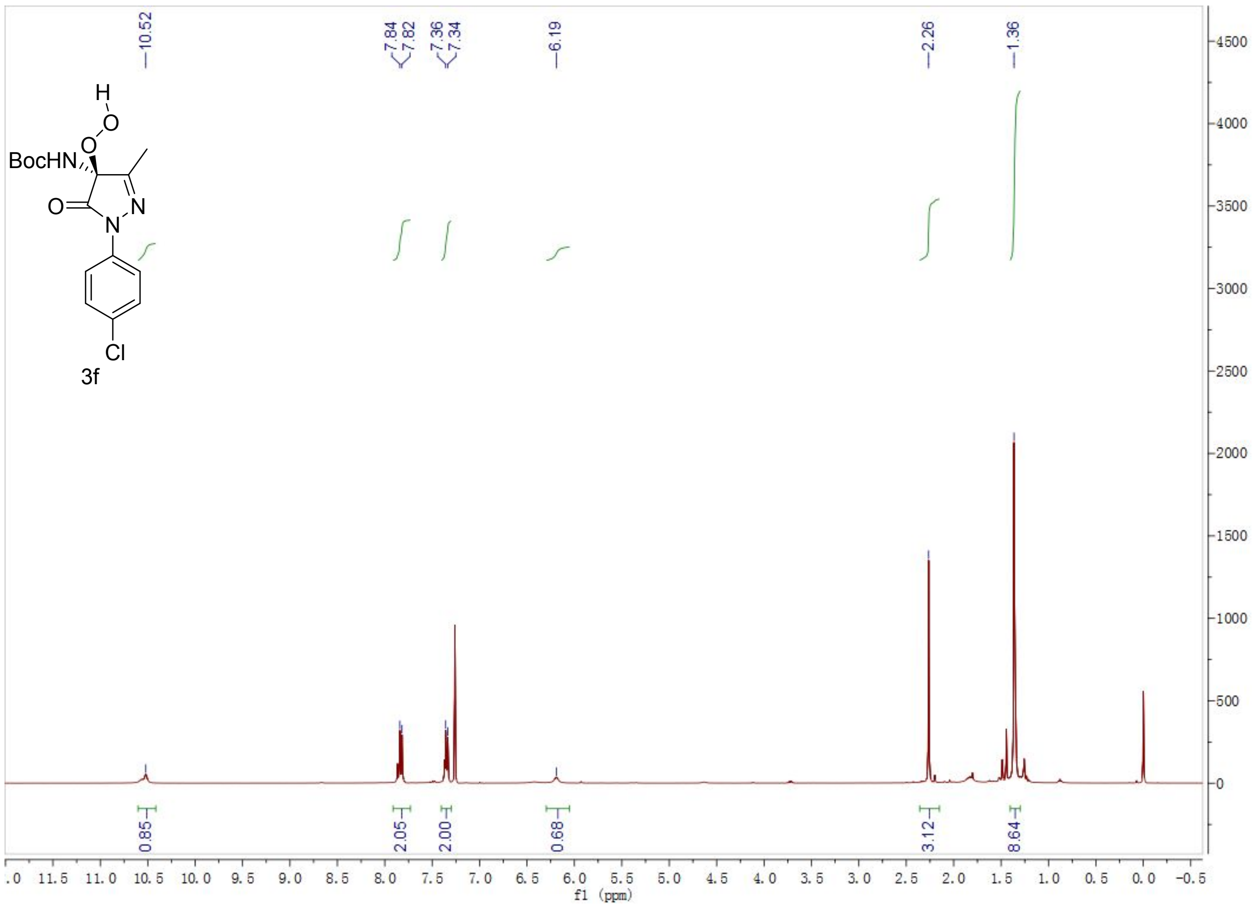
10.52
7.84
7.82
7.36
7.34
6.19
2.26
1.36
BocHN
3f
0.85
2.05
2.00
0.68
3.12
8.64
f1 (ppm)

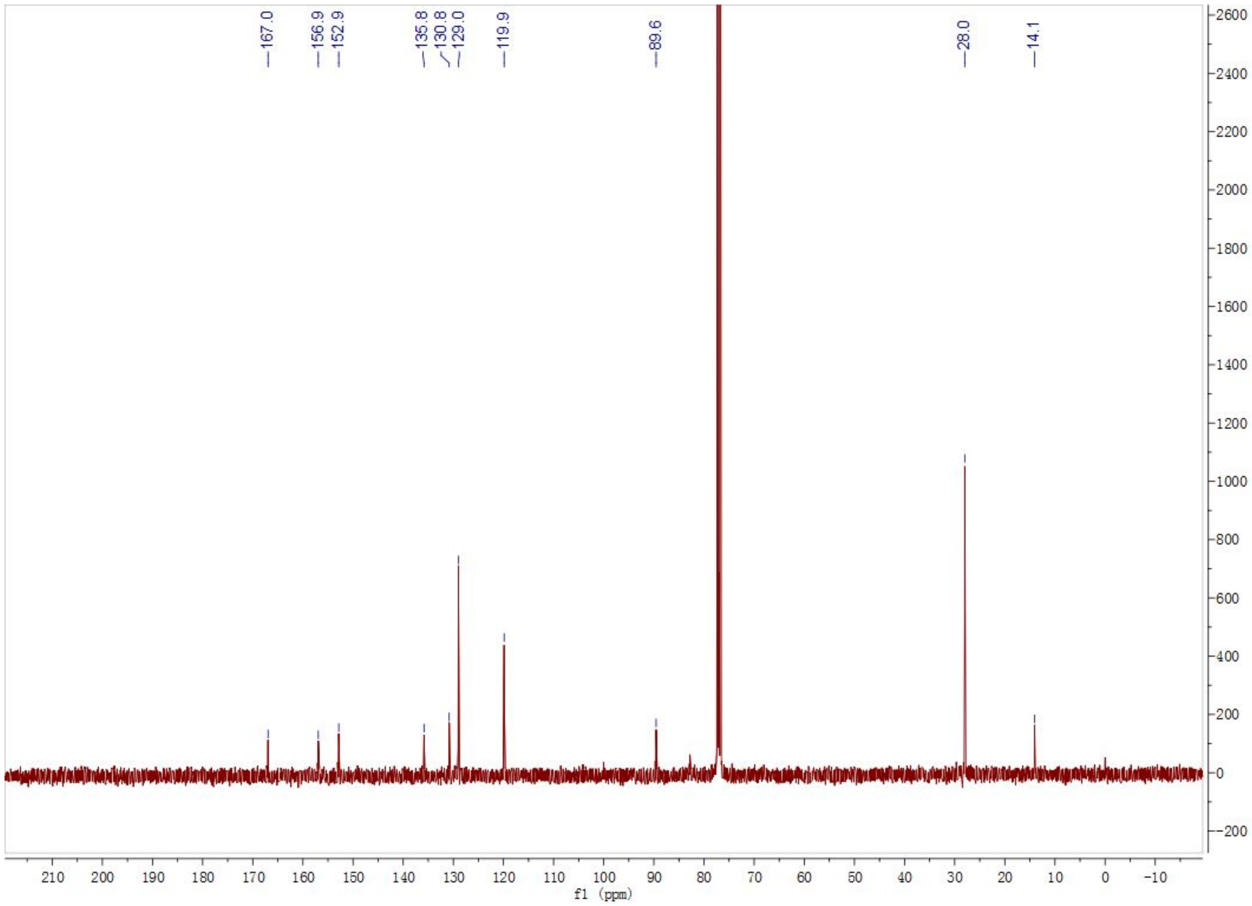
167.0
156.9
152.9
135.8
130.8
129.0
119.9
89.6
28.0
14.1
f1 (ppm)

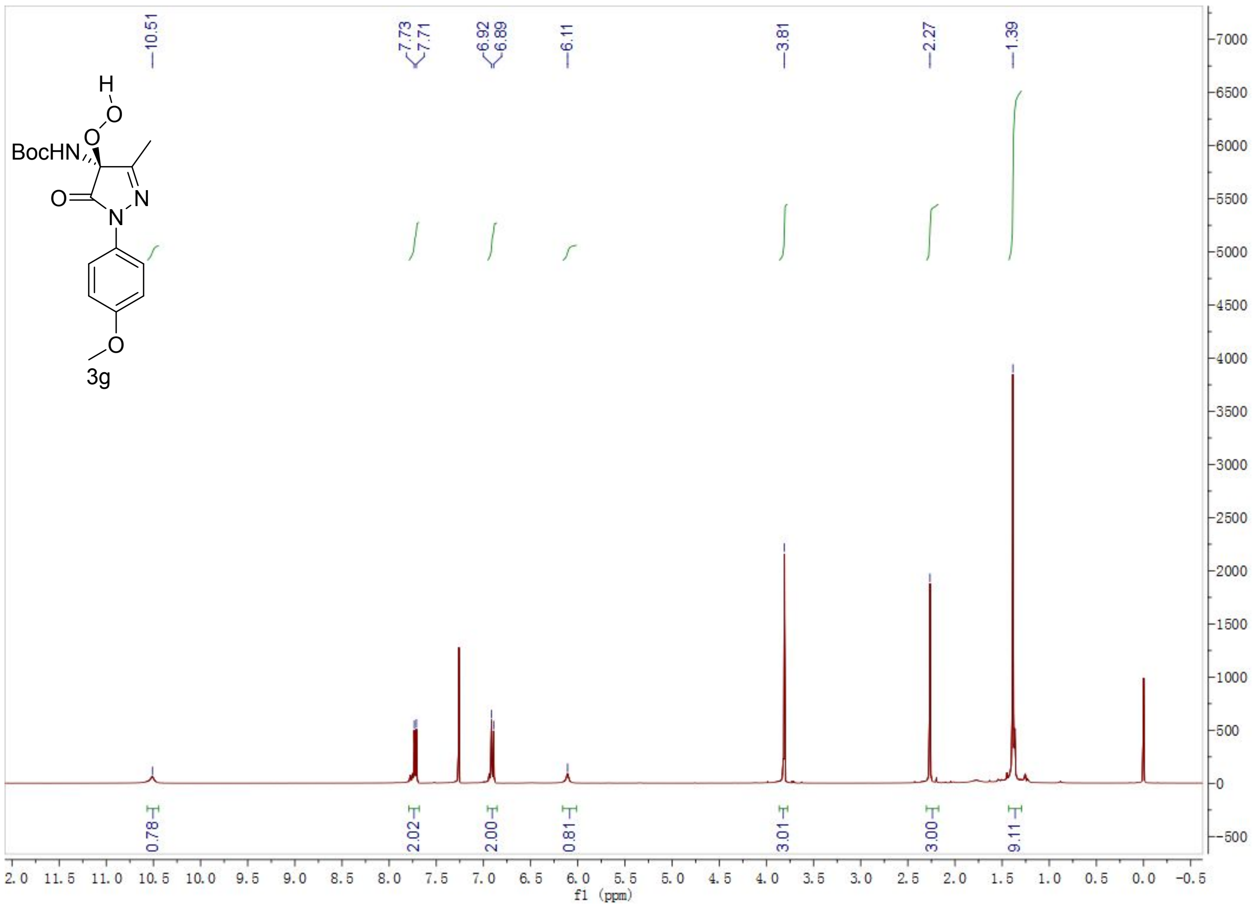

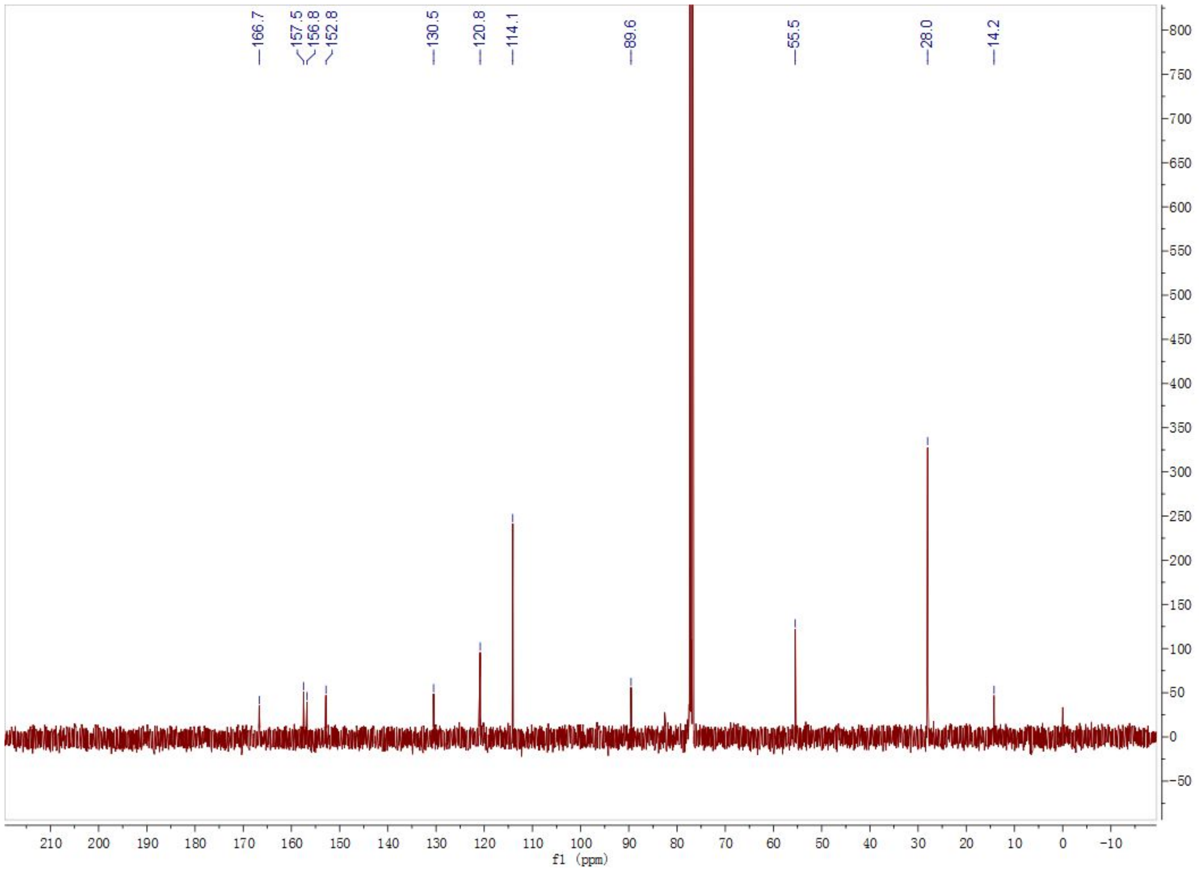

Figure S3. ^{1}H NMR and ^{13}C NMR spectra

5. HPLC spectra

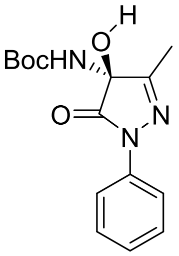

2a

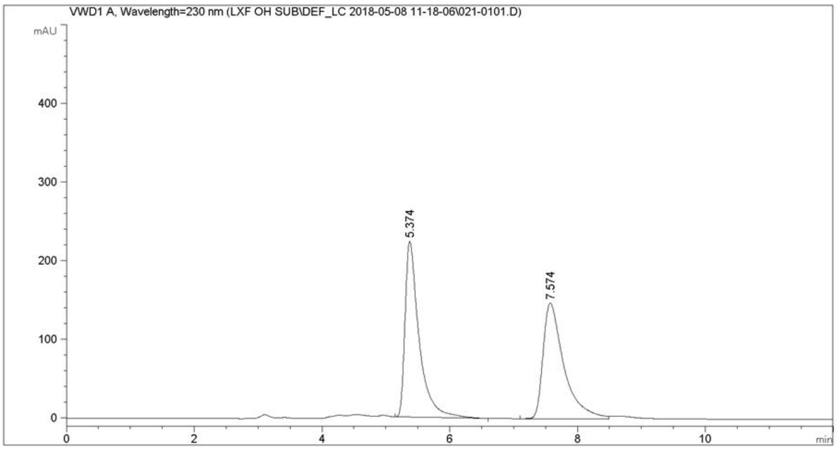

Peak #	RetTime [min]	Type	Width [min]	Area mAU *s	Height [mAU]	Area %
1	5.374	BB	0.2235	3412.47900	223.21648	50.6066
2	7.574	BV	0.3361	3330.66528	146.82703	49.3934

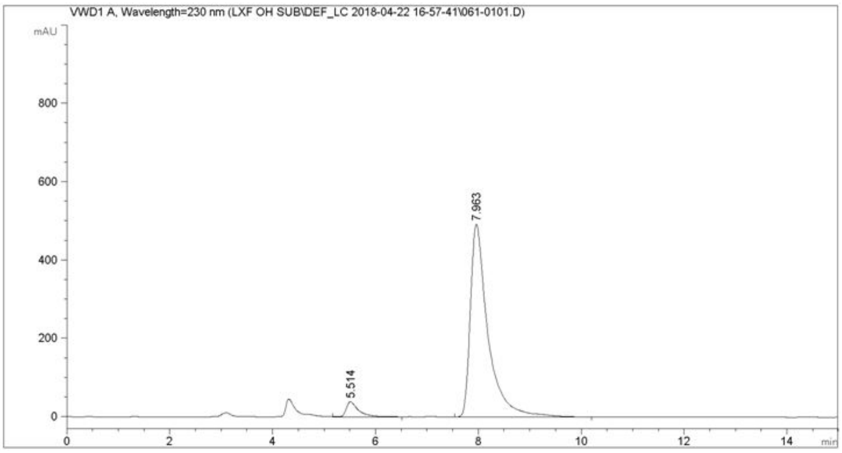

Peak #	RetTime [min]	Type	Width [min]	Area mAU *s	Height [mAU]	Area %
1	5.514	VB	0.2388	633.60126	38.47632	5.1530
2	7.963	BB	0.3440	1.16621e4	491.23462	94.8470

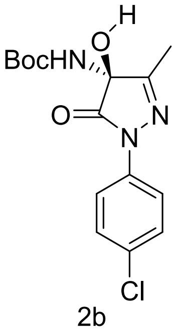

2b

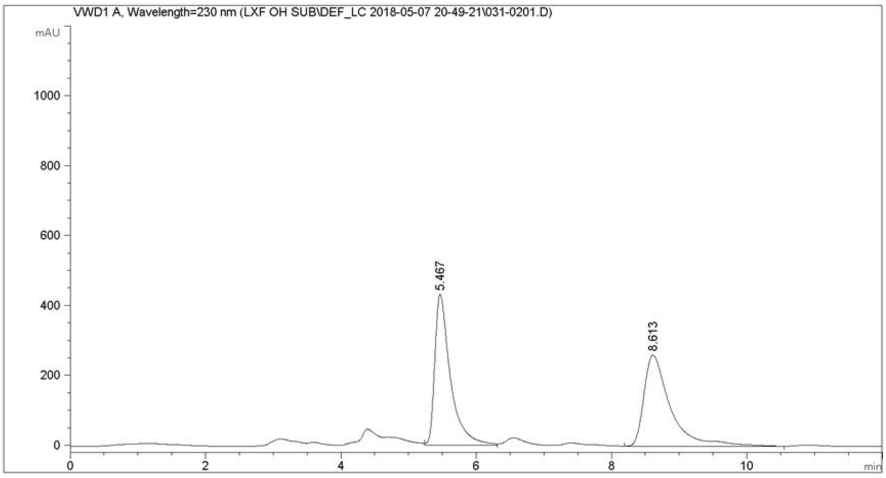

Peak #	RetTime [min]	Type	Width [min]	Area mAU *s	Height [mAU]	Area %
1	5.467	VV	0.2362	6976.53760	432.65619	49.4778
2	8.613	BV	0.3989	7123.80664	260.79605	50.5222

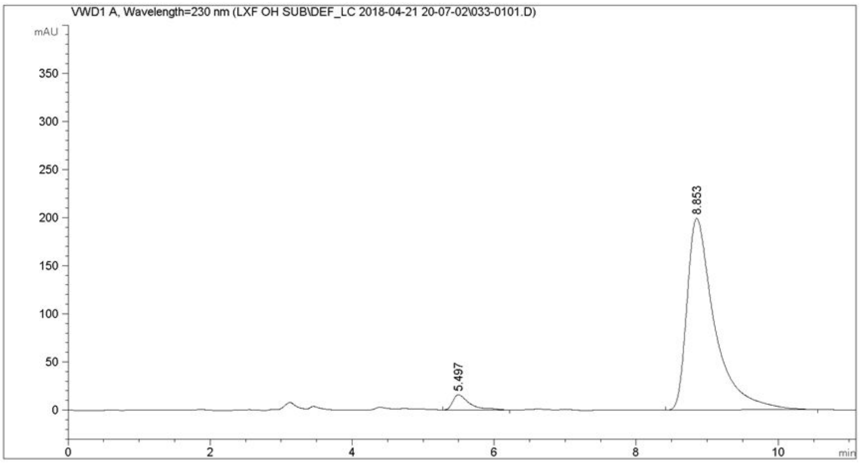

Peak #	RetTime [min]	Type	Width [min]	Area mAU *s	Height [mAU]	Area %
1	5.497	VB	0.2376	258.08078	15.89121	4.5674
2	8.853	BB	0.3975	5392.44189	199.14734	95.4326

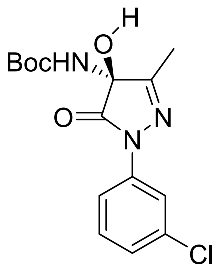

2c

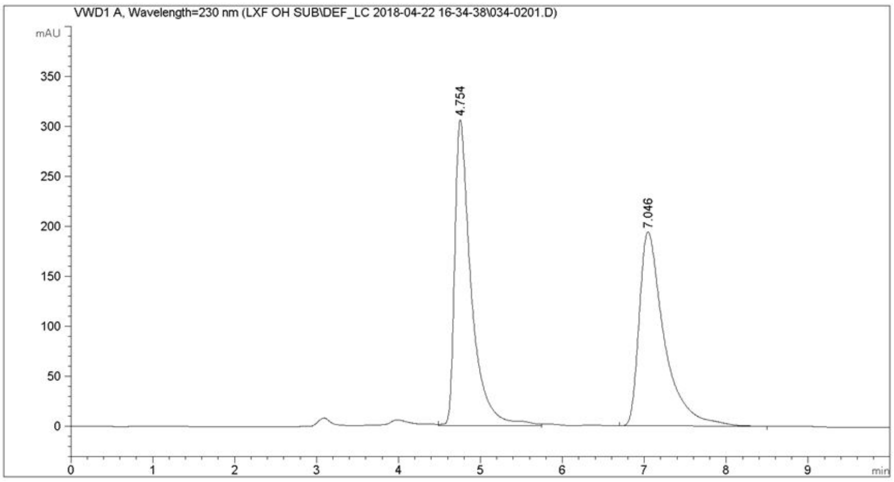

Peak #	RetTime [min]	Type	Width [min]	Area mAU *s	Height [mAU]	Area %
1	4.754	VV	0.2040	4281.76709	306.09811	50.9190
2	7.046	VB	0.3137	4127.21826	194.05269	49.0810

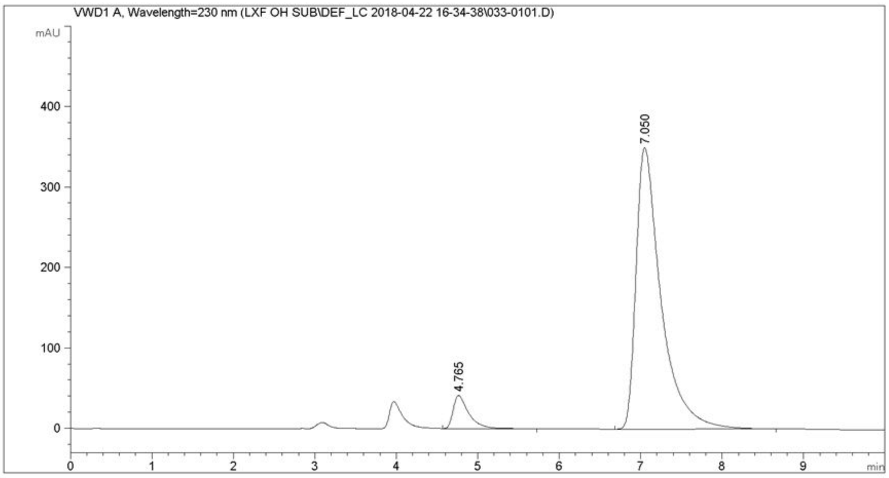

Peak #	RetTime [min]	Type	Width [min]	Area mAU *s	Height [mAU]	Area %
1	4.765	VB	0.2001	566.28235	41.47591	7.0509
2	7.050	BB	0.3145	7465.08545	349.83722	92.9491

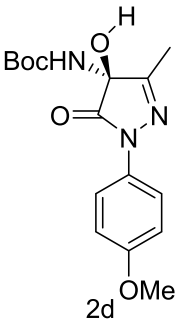

2d

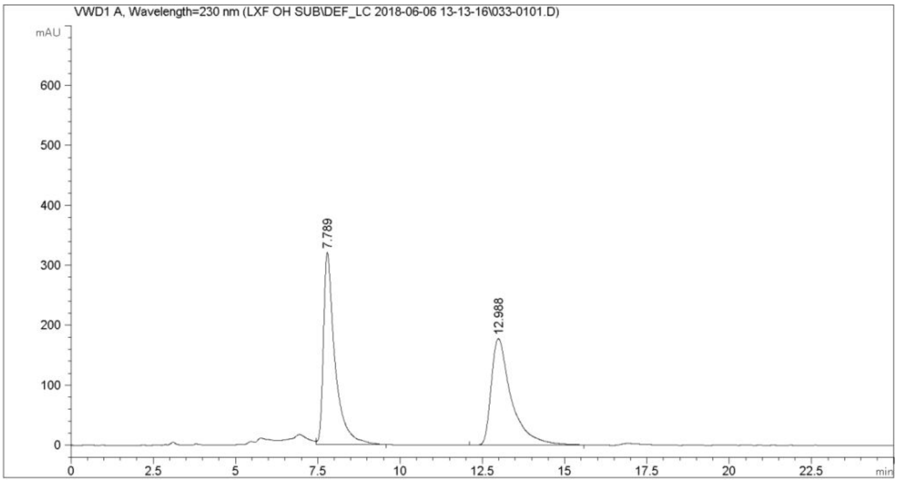

Peak #	RetTime [min]	Type	Width [min]	Area mAU *s	Height [mAU]	Area %
1	7.789	VB	0.3490	7783.23340	320.36191	50.8849
2	12.988	BB	0.6191	7512.53467	177.53372	49.1151

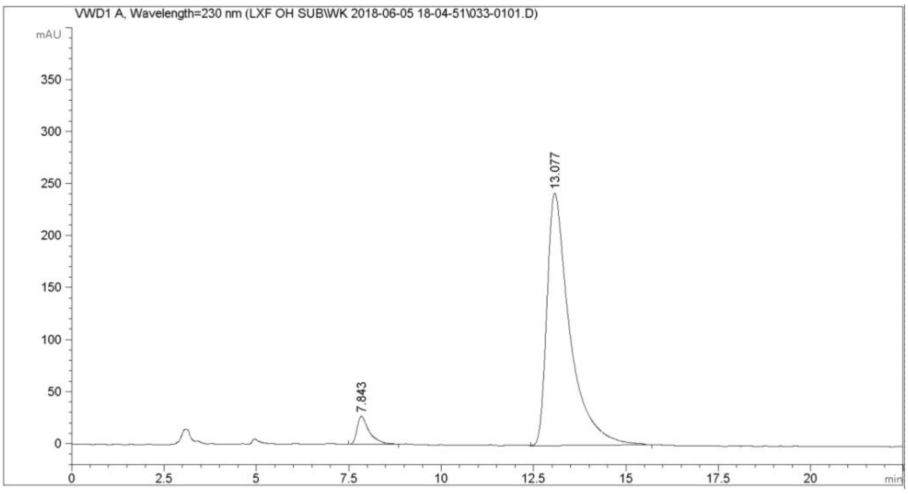

Peak #	RetTime [min]	Type	Width [min]	Area mAU *s	Height [mAU]	Area %
1	7.843	BB	0.3337	608.67871	26.77687	5.5145
2	13.077	BB	0.6239	1.04291e4	242.62405	94.4855

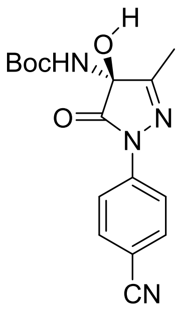

2e

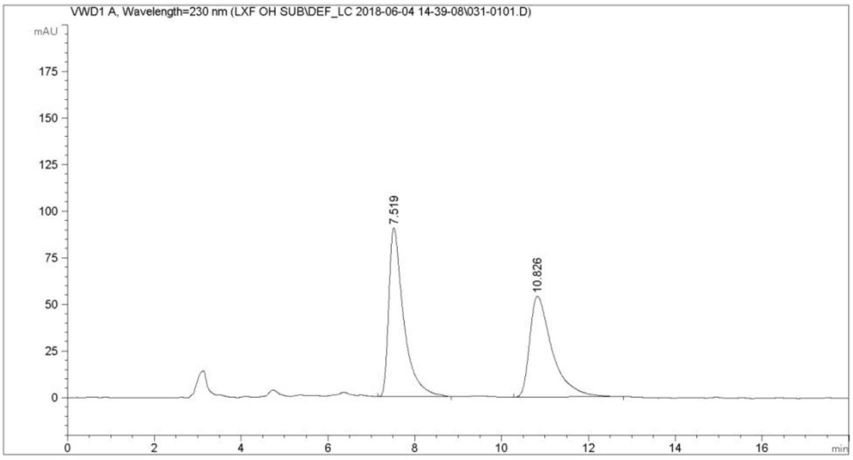

Peak #	RetTime [min]	Type	Width [min]	Area mAU *s	Height [mAU]	Area %
1	7.519	BB	0.3501	2163.82349	90.59423	52.6250
2	10.826	BB	0.5384	1947.95251	54.07396	47.3750

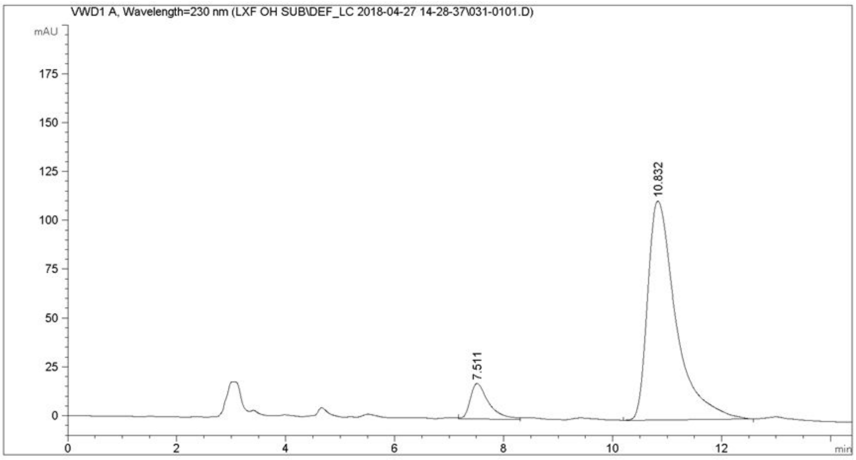

Peak #	RetTime [min]	Type	Width [min]	Area mAU *s	Height [mAU]	Area %
1	7.511	VV	0.3430	422.16791	18.13344	9.4687
2	10.832	BB	0.5400	4036.40430	111.99023	90.5313

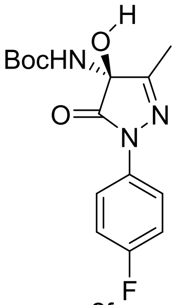

2f

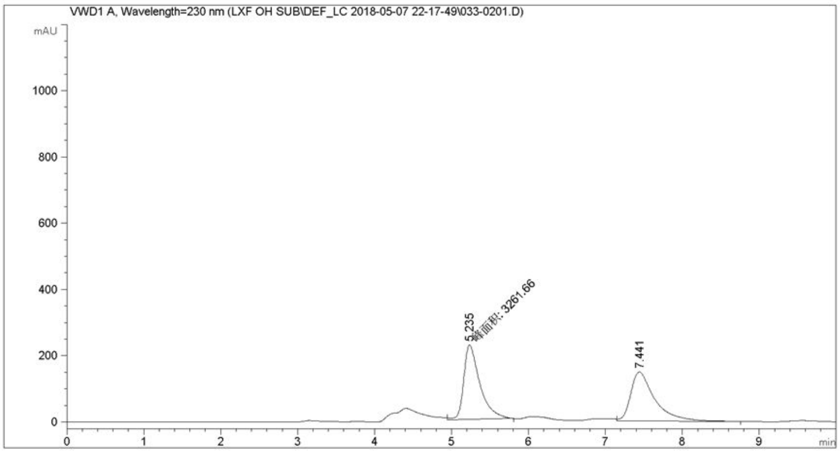

Peak #	RetTime [min]	Type	Width [min]	Area mAU *s	Height [mAU]	Area %
1	5.235	MM	0.2422	3261.65649	224.46219	50.5093
2	7.441	VB	0.3181	3195.87427	147.63475	49.4907

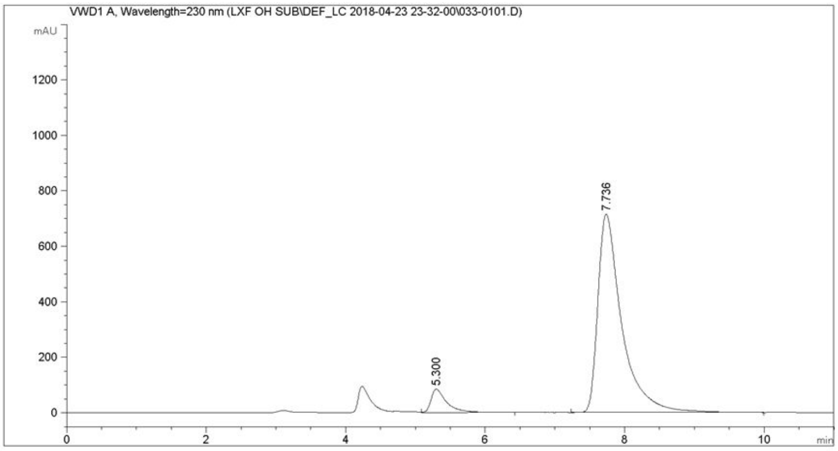

Peak #	RetTime [min]	Type	Width [min]	Area mAU *s	Height [mAU]	Area %
1	5.300	VB	0.2253	1310.27991	84.18790	7.1440
2	7.736	BB	0.3494	1.70307e4	714.71545	92.8560

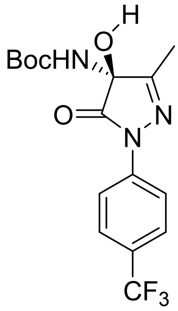

2g

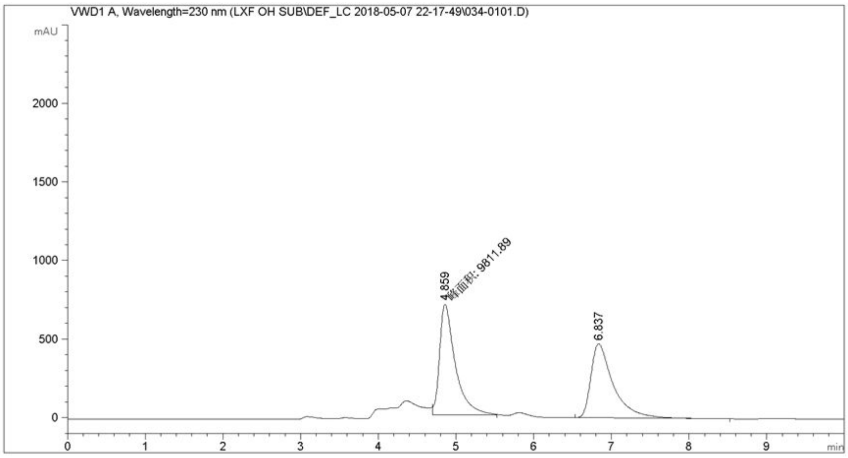

Peak #	RetTime [min]	Type	Width [min]	Area mAU *s	Height [mAU]	Area %
1	4.859	MM	0.2327	9811.88770	702.73907	50.9713
2	6.837	VB	0.2934	9437.94922	471.22244	49.0287

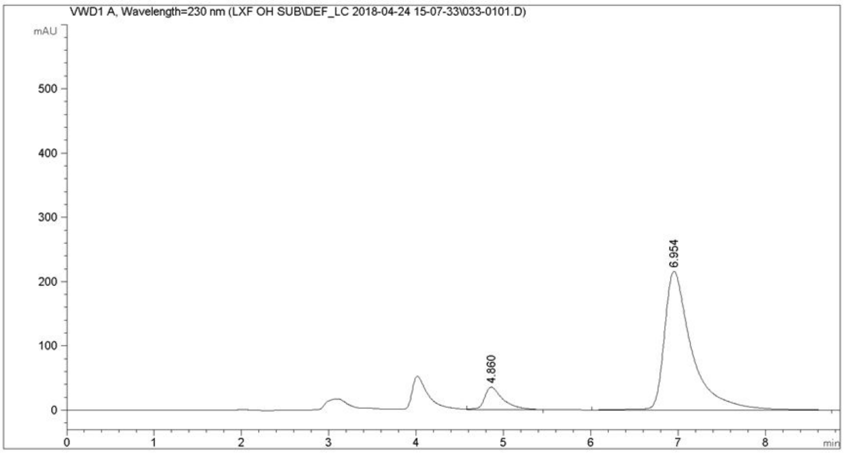

Peak #	RetTime [min]	Type	Width [min]	Area mAU *s	Height [mAU]	Area %
1	4.860	VB	0.2101	500.35754	34.78450	9.6570
2	6.954	BB	0.3159	4680.90967	215.63264	90.3430

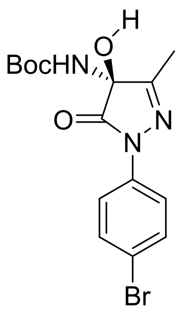

2h

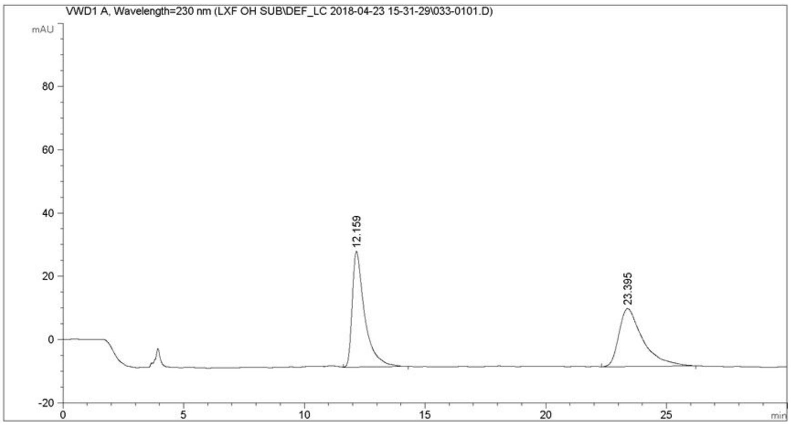

Peak #	RetTime [min]	Type	Width [min]	Area mAU *s	Height [mAU]	Area %
1	12.159	BB	0.5202	1303.90820	36.50495	51.0196
2	23.395	BB	0.9756	1251.79199	18.30656	48.9804

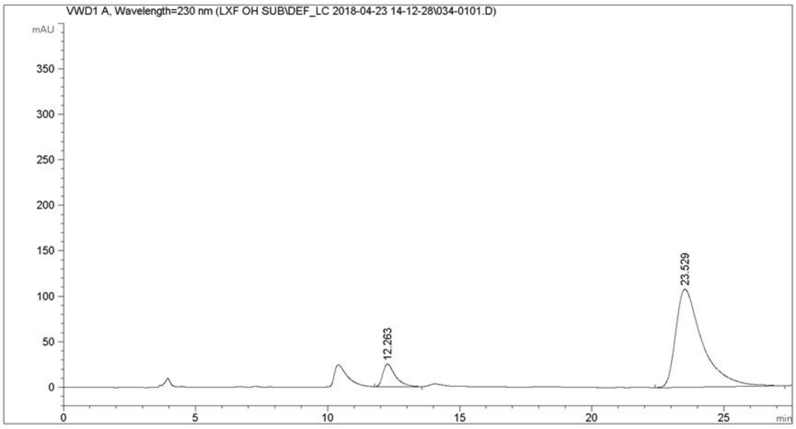

Peak #	RetTime [min]	Type	Width [min]	Area mAU *s	Height [mAU]	Area %
1	12.263	VB	0.4924	831.80676	25.04964	9.9705
2	23.529	BB	1.0217	7510.87012	107.74813	90.0295

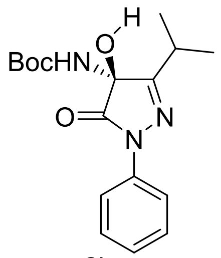

2i

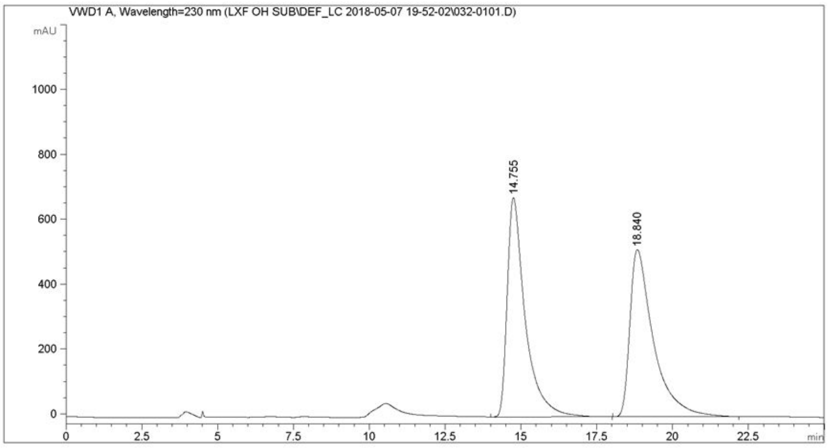

Peak #	RetTime [min]	Type	Width [min]	Area mAU *s	Height [mAU]	Area %
1	14.755	BB	0.6022	2.78543e4	675.30762	50.1719
2	18.840	BB	0.7767	2.76634e4	515.13165	49.8281

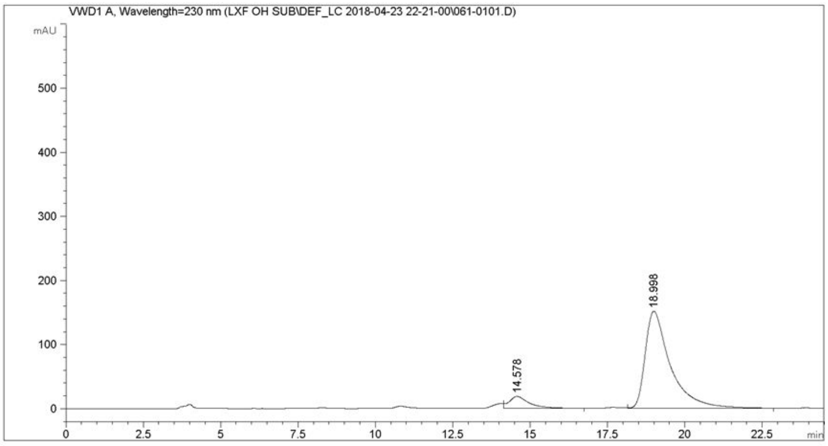

Peak #	RetTime [min]	Type	Width [min]	Area mAU *s	Height [mAU]	Area %
1	14.578	VB	0.6454	841.87512	18.62437	8.9292
2	18.998	VB	0.8316	8586.48242	151.54340	91.0708

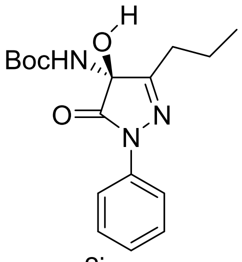

2j

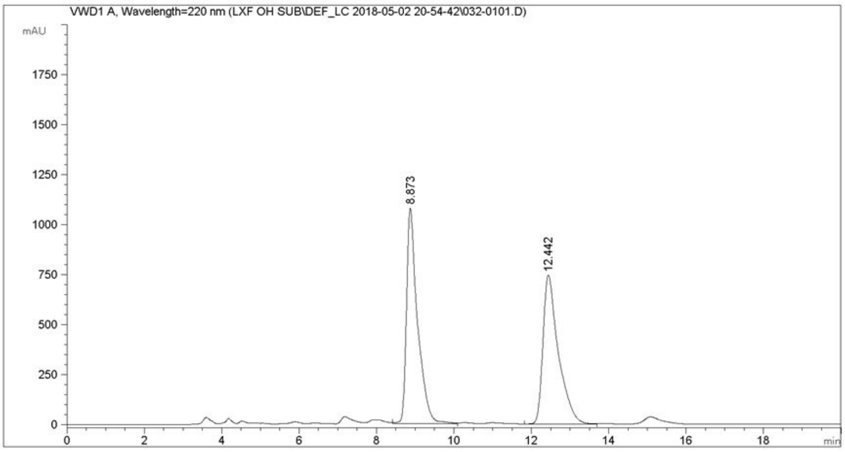

Peak #	RetTime [min]	Type	Width [min]	Area mAU *s	Height [mAU]	Area %
1	8.873	VV	0.2834	2.13418e4	1077.97815	51.0259
2	12.442	BV	0.3986	2.04836e4	743.45978	48.9741

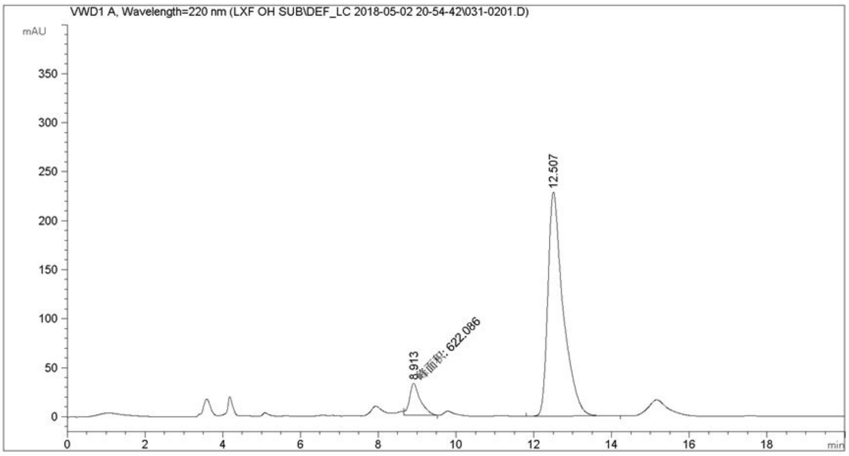

Peak #	RetTime [min]	Type	Width [min]	Area mAU *s	Height [mAU]	Area %
1	8.913	MM	0.3202	622.08649	32.38004	9.0842
2	12.507	BB	0.3948	6225.90625	228.68906	90.9158

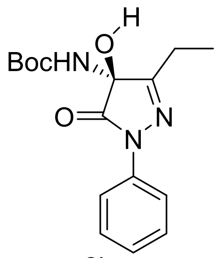

2k

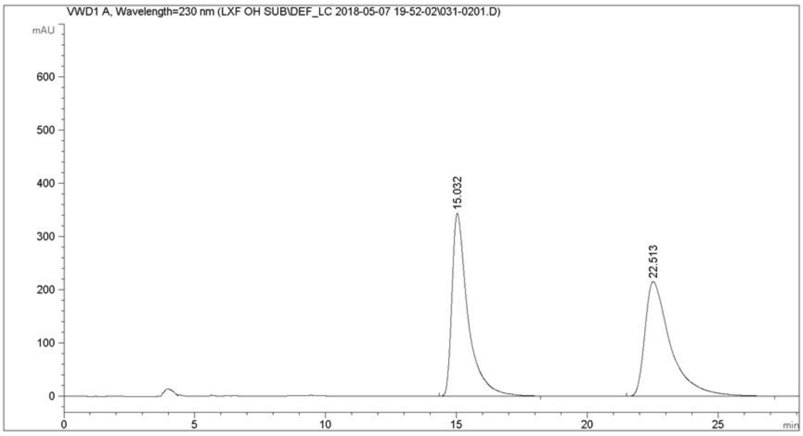

Peak #	RetTime [min]	Type	Width [min]	Area mAU *s	Height [mAU]	Area %
1	15.032	BB	0.6159	1.45425e4	343.80026	49.8969
2	22.513	BB	1.0127	1.46026e4	215.74944	50.1031

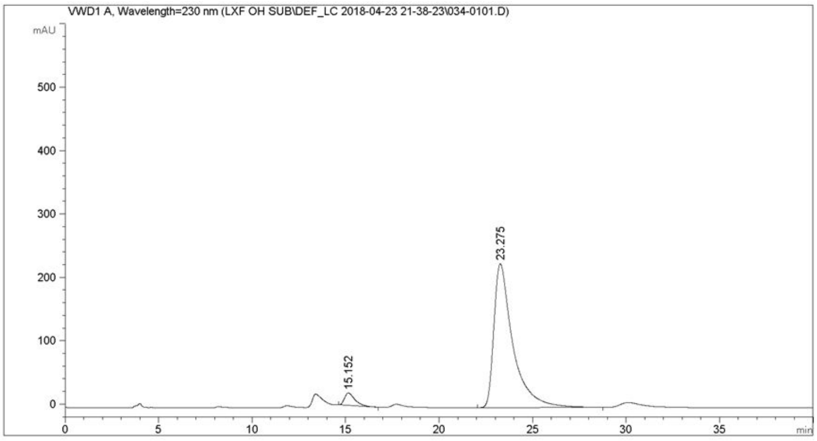

Peak #	RetTime [min]	Type	Width [min]	Area mAU *s	Height [mAU]	Area %
1	15.152	BB	0.5732	744.03198	19.44721	4.4388
2	23.275	BB	1.0265	1.60181e4	226.83171	95.5612

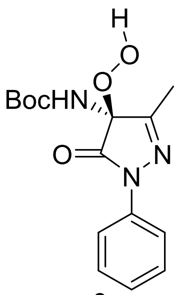

3a

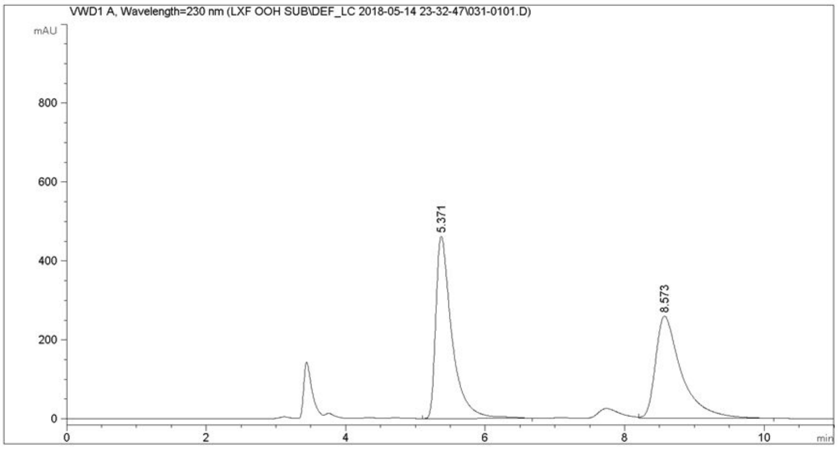

Peak #	RetTime [min]	Type	Width [min]	Area mAU *s	Height [mAU]	Area %
1	5.371	BB	0.2358	7369.57764	461.76117	52.5612
2	8.573	VB	0.3772	6651.37012	258.71564	47.4388

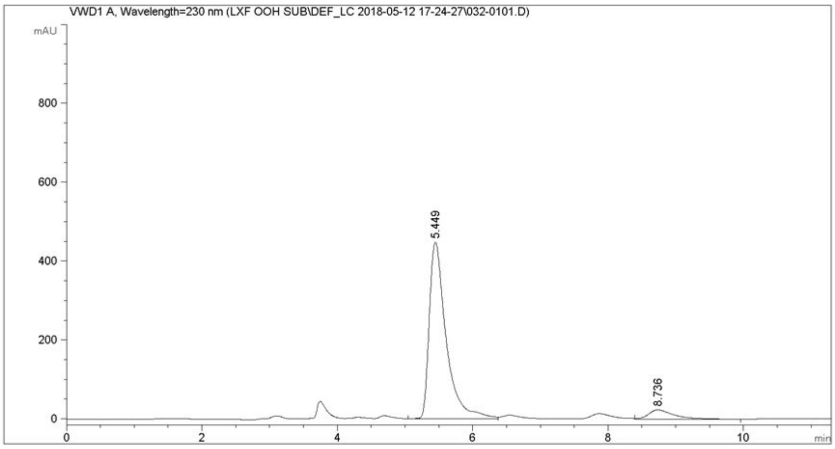

Peak #	RetTime [min]	Type	Width [min]	Area mAU *s	Height [mAU]	Area %
1	5.449	BV	0.2569	7742.97119	448.33997	92.7617
2	8.736	VB	0.3786	604.19629	23.27809	7.2383

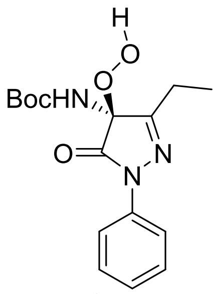

3b

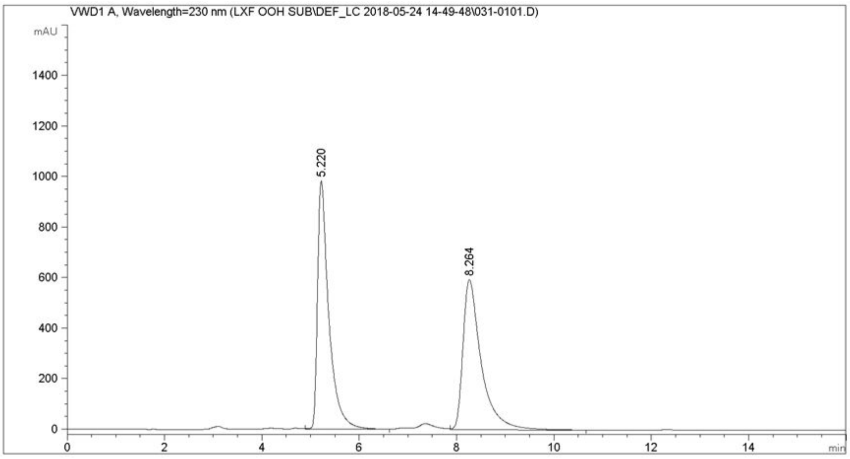

Peak #	RetTime [min]	Type	Width [min]	Area mAU *s	Height [mAU]	Area %
1	5.220	VB	0.2322	1.57790e4	984.39716	50.5986
2	8.264	VB	0.3816	1.54057e4	593.37866	49.4014

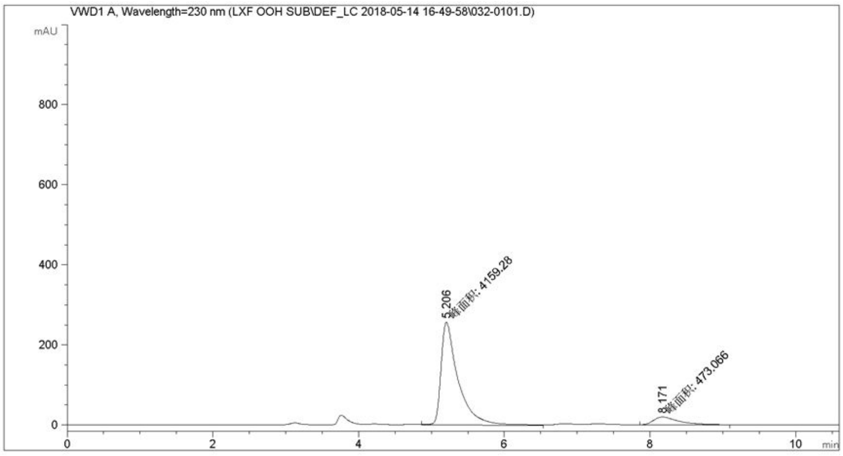

Peak #	RetTime [min]	Type	Width [min]	Area mAU *s	Height [mAU]	Area %
1	5.206	MM	0.2700	4159.28125	256.72037	89.7878
2	8.171	MM	0.4025	473.06619	19.58757	10.2122

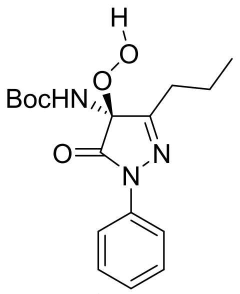

3c

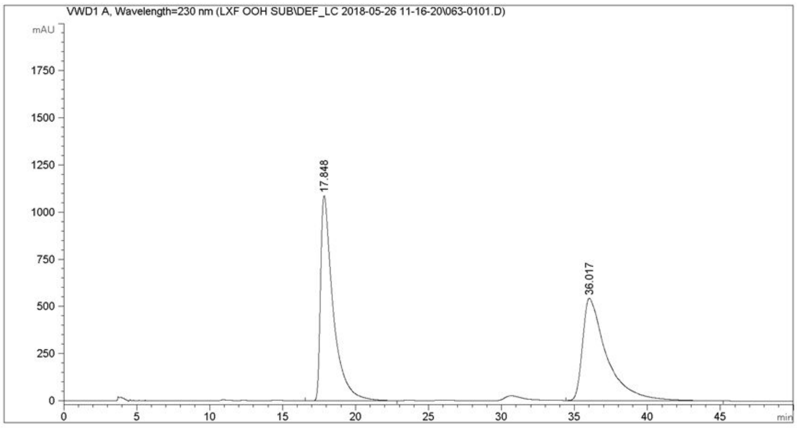

Peak #	RetTime [min]	Type	Width [min]	Area mAU *s	Height [mAU]	Area %
1	17.848	BB	0.8513	6.46933e4	1087.77356	51.0331
2	36.017	BB	1.7012	6.20742e4	542.93823	48.9669

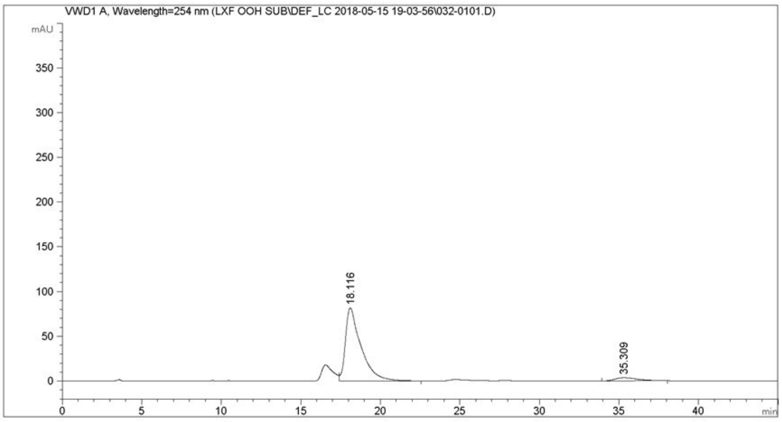

Peak #	RetTime [min]	Type	Width [min]	Area mAU *s	Height [mAU]	Area %
1	18.116	VB	0.9528	5550.43945	81.71638	93.9180
2	35.309	BB	1.2075	359.44180	3.54112	6.0820

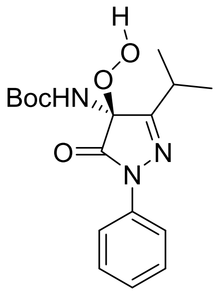

3d

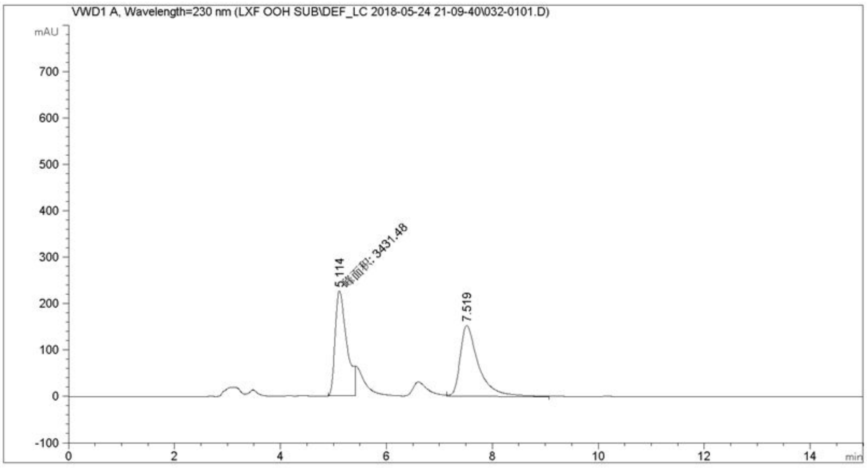

Peak #	RetTime [min]	Type	Width [min]	Area mAU *s	Height [mAU]	Area %
1	5.114	MM	0.2530	3431.48486	226.02600	48.4203
2	7.519	VV	0.3544	3655.38892	152.25816	51.5797

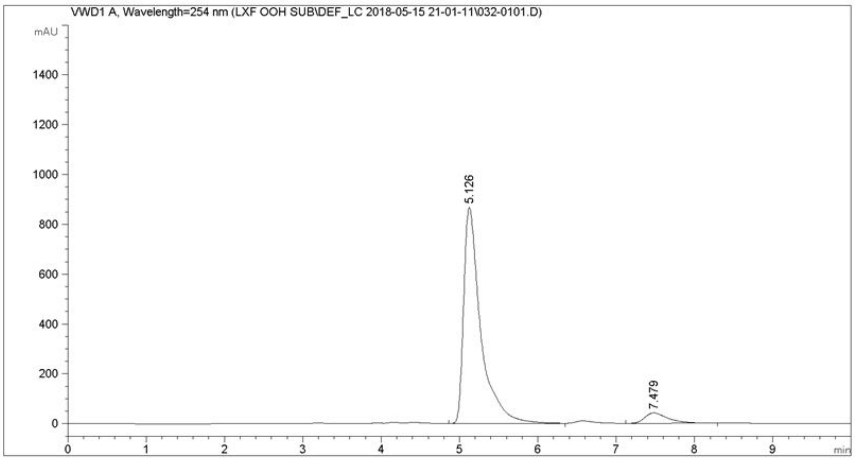

Peak #	RetTime [min]	Type	Width [min]	Area mAU *s	Height [mAU]	Area %
1	5.126	BV	0.2211	1.30706e4	866.76947	93.8393
2	7.479	BB	0.3082	858.10645	41.77145	6.1607

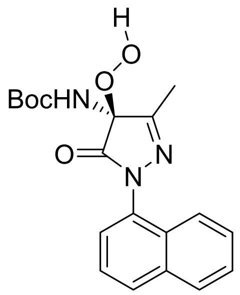

3e

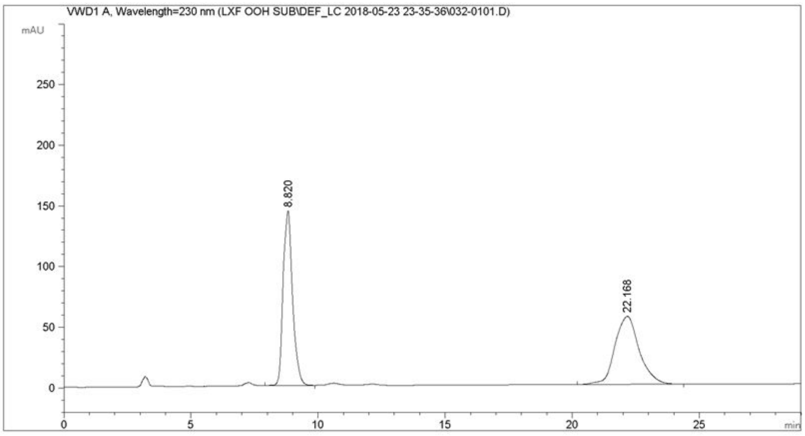

Peak #	RetTime [min]	Type	Width [min]	Area mAU *s	Height [mAU]	Area %
1	8.820	BB	0.4038	3712.46338	144.16393	49.9546
2	22.168	BB	1.0350	3719.21069	56.03141	50.0454

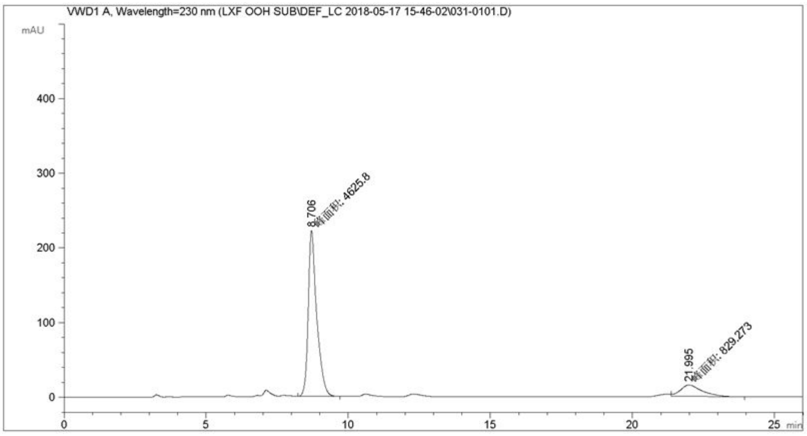

Peak #	RetTime [min]	Type	Width [min]	Area mAU *s	Height [mAU]	Area %
1	8.706	MM	0.3470	4625.80322	222.18033	84.7981
2	21.995	MM	0.8977	829.27319	15.39617	15.2019

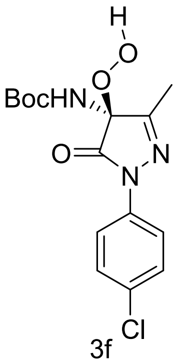

3f

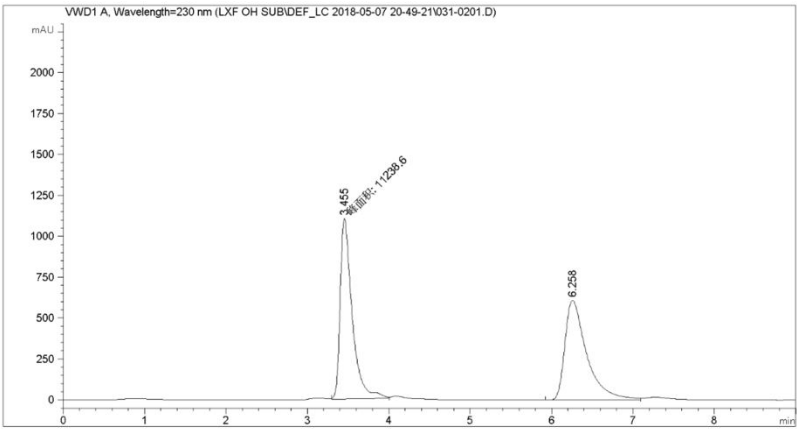

Peak #	RetTime [min]	Type	Width [min]	Area mAU *s	Height [mAU]	Area %
1	3.455	MM	0.1699	1.12386e4	1102.60815	49.8354
2	6.258	BV	0.2734	1.13128e4	609.56586	50.1646

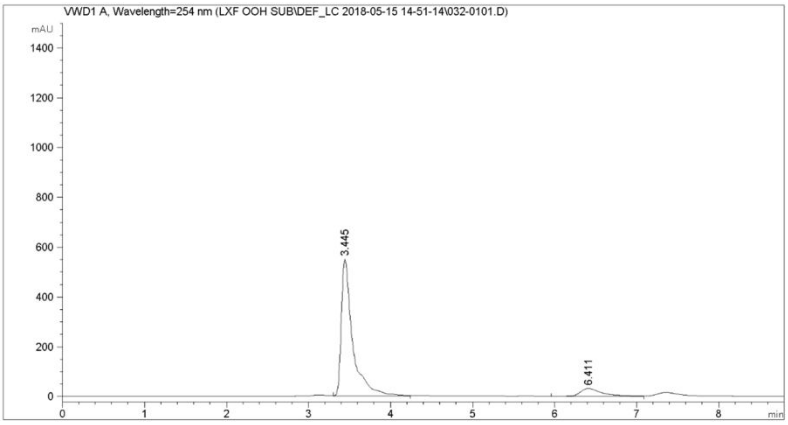

Peak #	RetTime [min]	Type	Width [min]	Area mAU *s	Height [mAU]	Area %
1	3.445	VV	0.1400	5361.60254	550.06622	90.0120
2	6.411	BV	0.2733	594.93811	31.64065	9.9880

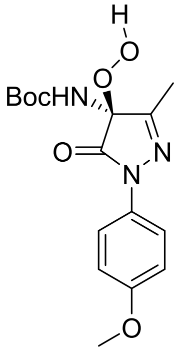

3g

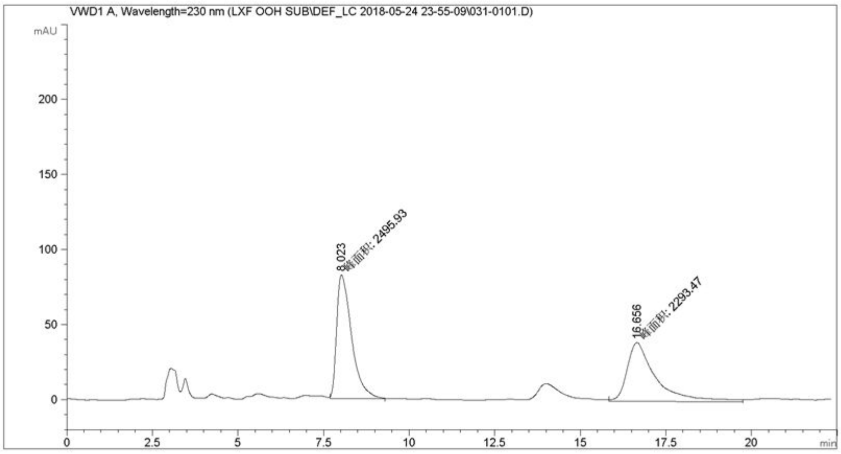

Peak #	RetTime [min]	Type	Width [min]	Area mAU *s	Height [mAU]	Area %
1	8.023	MM	0.5047	2495.93384	82.42673	52.1137
2	16.656	MM	0.9746	2293.46802	39.22021	47.8863

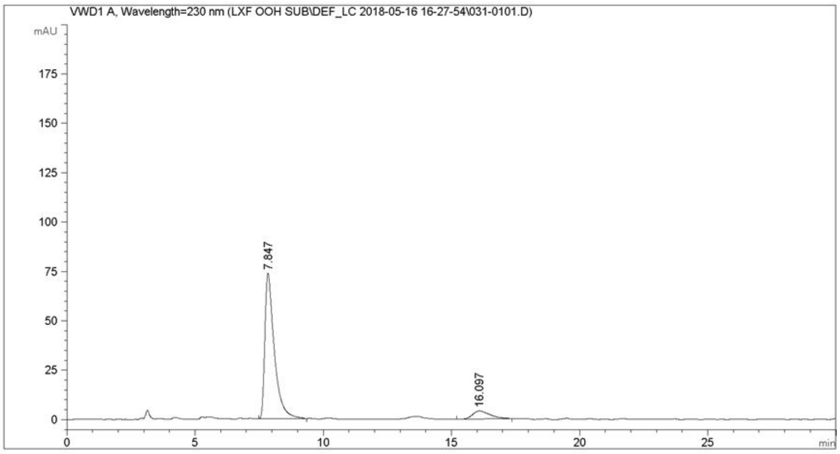

Peak #	RetTime [min]	Type	Width [min]	Area mAU *s	Height [mAU]	Area %
1	7.847	BB	0.3691	1868.29944	73.58878	90.3437
2	16.097	BB	0.6368	199.69153	4.13148	9.6563

Figure S4. HPLC spectra

6. LC-MS of [m+16], [m+32], [m+48]

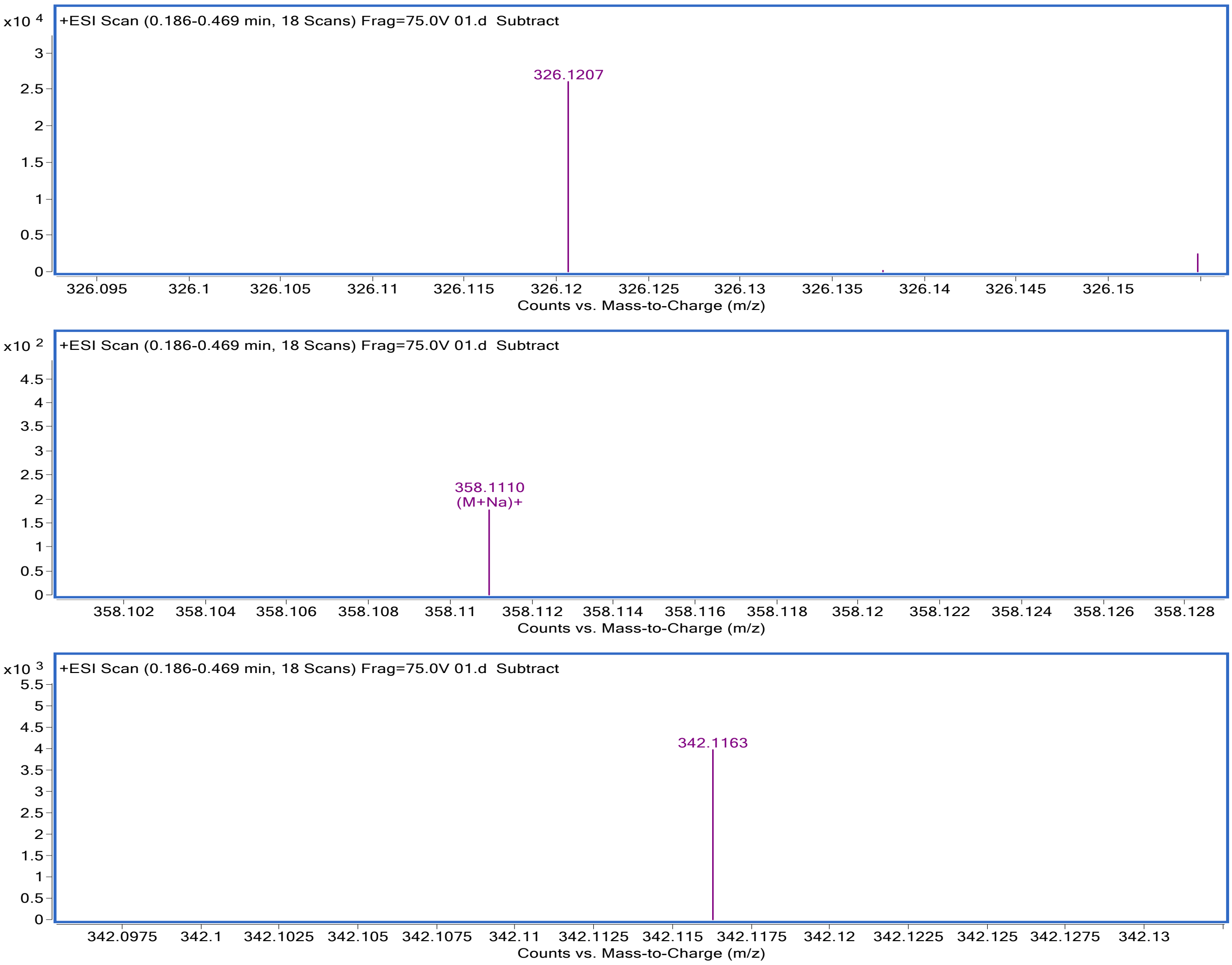

Figure S5. LC-MS of [m+16], [m+32], [m+48]
